# Sanglifehrin A mitigates multiorgan fibrosis by targeting the collagen chaperone cyclophilin B

**DOI:** 10.1172/jci.insight.171162

**Published:** 2024-06-20

**Authors:** Hope A. Flaxman, Maria-Anna Chrysovergi, Hongwei Han, Farah Kabir, Rachael T. Lister, Chia-Fu Chang, Robert Yvon, Katharine E. Black, Andreas Weigert, Rajkumar Savai, Alejandro Egea-Zorrilla, Ana Pardo-Saganta, David Lagares, Christina M. Woo

**Affiliations:** 1 Department of Chemistry and Chemical Biology, Harvard University, Cambridge, Massachusetts, USA.; 2 Fibrosis Research Center, Center for Immunology and Inflammatory Diseases, Division of Pulmonary and Critical Care Medicine, Massachusetts General Hospital, Harvard Medical School, Boston, Massachusetts, USA.; 3 Goethe-University Frankfurt, Faculty of Medicine, Institute of Biochemistry I, Germany.; 4 Frankfurt Cancer Institute (FCI), Goethe University, and German Cancer Consortium (DKTK), Germany.; 5 Max Planck Institute for Heart and Lung Research, Member of the German Center for Lung Research (DZL), Bad Nauheim, Germany.; 6 Institute for Lung Health (ILH), Department of Internal Medicine, Justus-Liebig University, Universities of Giessen and Marburg Lung Center (UGMLC), DZL, Giessen, Germany.; 7 Cardio-Pulmonary Institute (CPI), Department of Internal Medicine, Justus Liebig University, Giessen, Germany.

**Keywords:** Inflammation, Therapeutics, Collagens, Drug therapy, Fibrosis

## Abstract

Pathological deposition and crosslinking of collagen type I by activated myofibroblasts drives progressive tissue fibrosis. Therapies that inhibit collagen synthesis have potential as antifibrotic agents. We identify the collagen chaperone cyclophilin B as a major cellular target of the natural product sanglifehrin A (SfA) using photoaffinity labeling and chemical proteomics. Mechanistically, SfA inhibits and induces the secretion of cyclophilin B from the endoplasmic reticulum (ER) and prevents TGF-β1–activated myofibroblasts from synthesizing and secreting collagen type I in vitro, without inducing ER stress or affecting collagen type I mRNA transcription, myofibroblast migration, contractility, or TGF-β1 signaling. In vivo, SfA induced cyclophilin B secretion in preclinical models of fibrosis, thereby inhibiting collagen synthesis from fibrotic fibroblasts and mitigating the development of lung and skin fibrosis in mice. Ex vivo, SfA induces cyclophilin B secretion and inhibits collagen type I secretion from fibrotic human lung fibroblasts and samples from patients with idiopathic pulmonary fibrosis (IPF). Taken together, we provide chemical, molecular, functional, and translational evidence for demonstrating direct antifibrotic activities of SfA in preclinical and human ex vivo fibrotic models. Our results identify the cellular target of SfA, the collagen chaperone cyclophilin B, as a mechanistic target for the treatment of organ fibrosis.

## Abstract 2

Sanglifehrin A targets cyclophiln B to reduce collagen synthesis, supporting further evaluation of this natural product as a potential treatment for lung and skin fibrosis.

## Graphical abstract

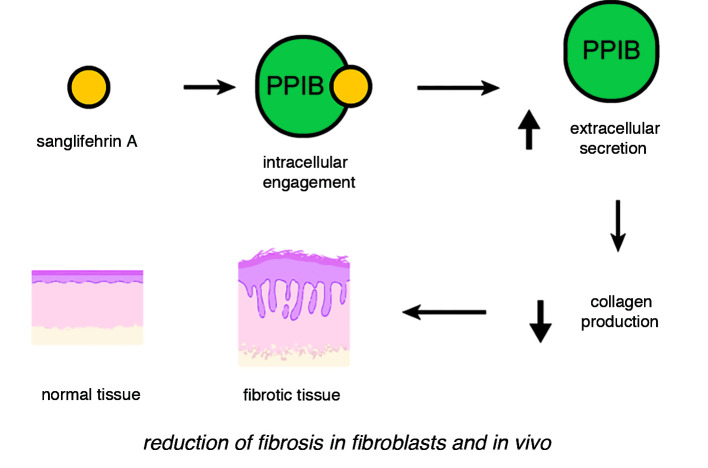

## Introduction

Fibrosis is a pathological process characterized by excessive deposition of collagen-rich extracellular matrix in response to chronic or overwhelming tissue injury, ultimately leading to the development of fibrotic diseases that can affect nearly every organ including the skin, lungs, liver, and kidneys (1–6). Skin and lung fibrosis are hallmarks of fatal fibrotic diseases such as systemic sclerosis (SSc), an autoimmune multiorgan fibrotic disease (7, 8), and idiopathic pulmonary fibrosis (IPF), an age-related interstitial lung disease (9). In these diseases, tissue fibrogenesis is driven by chronic epithelial and vascular damage, type 2 inflammation, and activation of scar-forming cells known as myofibroblasts (10, 11). Efforts to understand the biology of myofibroblasts have led to the identification of 2 molecules, pirfenidone and nintedanib, which prevent collagen synthesis induced by the profibrotic cytokine TGF-β1 (12, 13). Despite being approved for clinical use, the mechanisms of action of pirfenidone and nintedanib remain incompletely understood. Given the modest efficacy and low tolerability of the current generation of antifibrotic therapies, there is a continuing need to characterize antifibrotic agents targeting myofibroblast activation and collagen synthesis with greater selectivity and improved efficacy (1).

Drug repurposing is an attractive alternative to de novo development of antifibrotic therapies due to the use of derisked compounds, some of which are already tested in clinical trials, potentially shortening development timelines, although one of the challenges related to this strategy is the identification of specific targets blocked by agents with antifibrotic properties. Chemical proteomics methods like photoaffinity labeling (PAL) enable unbiased target identification studies inside live cells. In a PAL experiment, a small molecule probe functionalized with a photoactivatable group is added to cells and covalently conjugated to interacting protein targets upon UV irradiation for enrichment and identification by mass spectrometry (MS) (14, 15).

Here, we apply chemical proteomics to the natural product sanglifehrin A (SfA). SfA is a 22-membered macrocycle decorated with a unique spirolactam that was identified in 1999 by Novartis in a screen for bacterially produced compounds that bind to the peptidyl-prolyl *cis*-*trans* isomerase cyclophilin A (PPIA) (16, 17). PPIA is also the target of the immunosuppressive drug cyclosporin A (CsA), and the PPIA:CsA complex binds to and inhibits the phosphatase calcineurin, ultimately blocking the proliferation of activated T cells (18–21). Prior investigations have shown that SfA has immunosuppressant properties that are mechanistically distinct from other immunosuppressants, including CsA and rapamycin (22), and that SfA blocks cell proliferation at the G1-S phase transition by induction of p53 expression via NF-κB signaling (23, 24). SfA additionally forms a ternary complex with PPIA and the cystathionine β synthase domain of inosine monophosphate dehydrogenase 2 (IMPDH2) (25), and our structure-activity relationship studies suggest that SfA may exert its effects through additional targets (26). While SfA engages several cyclophilins by in vitro biochemical assays (17), an accurate profile of the interactions of SfA across all cyclophilins in live cells is challenging, where contributions from binding affinities, the subcellular localization, and protein levels contribute to the interaction landscape. We therefore used a chemical proteomics approach to discover that SfA primarily interacts with the endoplasmic reticulum–resident (ER-resident) peptidyl-prolyl *cis-trans* isomerase cyclophilin B (*PPIB* gene and PPIB protein) in live cells and shows subsequent secretion of PPIB from the ER to the extracellular space, which may play a role in the inhibition of collagen folding by SfA in myofibroblasts in vitro, in vivo, and in lung fibrotic tissue from patients with IPF.

## Results

### Identification of cyclophilin B as a target of SfA in live cells.

**Figure 1:**
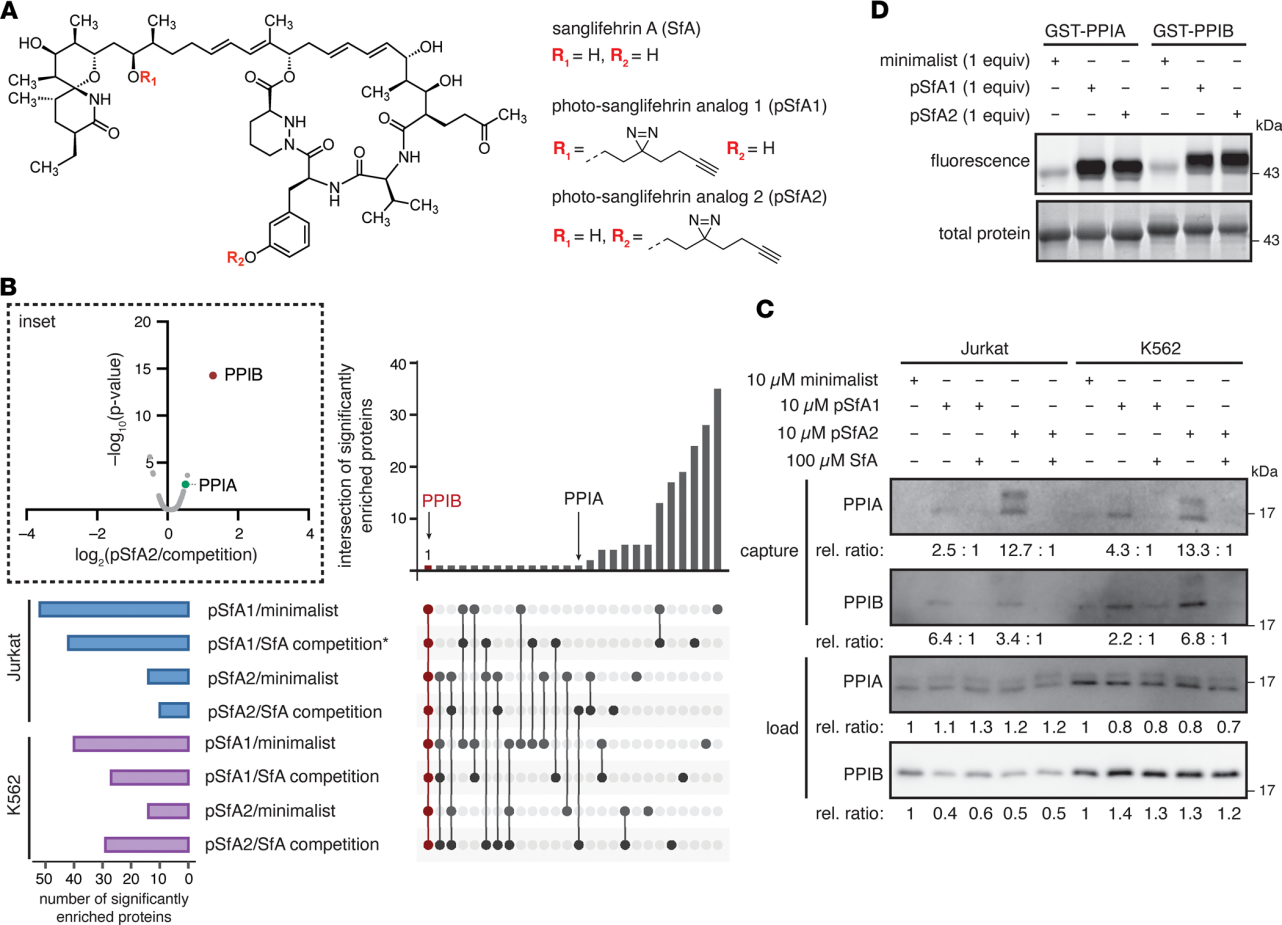
Development of pSfA probes for target identification from live cells. (**A**) Structures of SfA, pSfA1, and pSfA2. (**B**) Summary of significantly enriched (fold change > 1, *P* < 0.05) proteins identified by proteomics following treatment with 10 μM pSfA1 or pSfA2, with and without competition with 10× SfA (100 μM), or the minimalist tag alone (10 μM), in Jurkat or K562 cells treated for 30 minutes prior to photoaffinity labeling (*n* = 3). *n = 2, due to loss of 1 sample in the comparison of pSfA1/competition. Inset: Example volcano plot showing significant and competitive enrichment of PPIB by pSfA2. (**C**) Western blot and relative quantification for PPIA and PPIB after enrichment of pSfA1- or pSfA2-labeled proteins from Jurkat or K562 cells. (**D**) In vitro labeling of recombinant GST-PPIA and GST-PPIB with pSfA probes visualized by attachment of Alexa Fluor 488 azide and in-gel fluorescence.

To investigate the targets of SfA by chemical proteomics, we synthesized 2 photosanglifehrin probes, pSfA1 and pSfA2, by functionalization of SfA at different positions with the minimalist tag (Figure 1A) (27). We designed 2 pSfA probes to ensure that at least 1 probe retained activity and to potentially capture a wider range of protein targets (Supplemental Figure 1, A–C; supplemental material available online with this article; https://doi.org/10.1172/jci.insight.171162DS1). Cell viability profiles of the probes in Jurkat and K562 cells were assessed as a proxy for immunosuppressive activity in T and B cells, respectively, and had mild activity similar to SfA (25) (Table 1 and Supplemental Figure 2, A and B). No antiproliferative activity was observed in A549 cells, indicating that pSfA probes, like SfA (25), are not broadly cytotoxic (IC_50_ > 10 μM; Supplemental Figure 2C). Using a time-resolved Förster resonance energy transfer (TR-FRET) binding assay, the observed dissociation constants for SfA and the pSfA probes to PPIA and PPIB are generally comparable, although pSfA2 has reduced engagement of PPIA (Table 1 and Supplemental Figure 3). SfA binds at the highly conserved cyclophilin active site (28), which inhibits the enzymatic peptidyl-prolyl *cis-trans* isomerase (PPI) activity of these enzymes (29).

We next performed chemical proteomics using the pSfA probes in Jurkat and K562 cells to identify target proteins of SfA in live cells in an unbiased manner. Proteins enriched with pSfA1 or pSfA2 from each cell line were considered targets for SfA if they were selectively competed with a 10-fold excess of SfA or were enriched relative to nonspecific labeling with the minimalist tag. Among the biological targets of SfA identified by chemical proteomics in live cells (Supplemental Table 1), only PPIB was enriched significantly (log_2_[fold change] > 1, *P* < 0.05])across all 8 ratios, which represents comparison with SfA competition and background from the minimalist tag alone across 2 cell lines and 2 probes (Figure 1B; Supplemental Figure 4, A–C; Supplemental Figure 5; and Supplemental Table 1). By contrast, PPIA was significantly enriched by pSfA2 versus competition in Jurkat and K562 cells in 2 of the 8 ratios (Figure 1B; Supplemental Figure 4, A–C; and Supplemental Table 1). Other PPIases, including mitochondrial PPIF, were observed but not significantly enriched (Supplemental Table 1). The observed cyclophilin interactions were validated by Western blot, which showed that indeed PPIB is labeled to a greater degree than the minimalist tag (30) and is enriched and competed in both cell lines by pSfA1 and pSfA2 (Figure 1C). PPIA is labeled and enriched to a greater extent with pSfA2 after competition with SfA, in alignment with the MS results (Figure 1, B and C). The pSfA2-treated samples further show a higher molecular weight band for PPIA after enrichment, potentially representing an observable mass shift due to pSfA2 labeling. IMPDH2, a previously identified target of SfA (25), showed some labeling by the pSfA probes, although it did not meet significance thresholds in MS data (Supplemental Figure 4D and Supplemental Table 1). Despite the preference for PPIB observed in cells, both pSfA probes labeled recombinant PPIA and PPIB similarly by in-gel fluorescence (Figure 1D). Confocal imaging of pSfA2 with the ER marker calnexin demonstrates the localization of SfA within the ER (Supplemental Figure 6). These results indicate that the pSfA probes and SfA interact to a greater extent with PPIB than any other cyclophilins in the cell.

### SfA induces secretion of cyclophilin B into the extracellular space.

**Figure 2:**
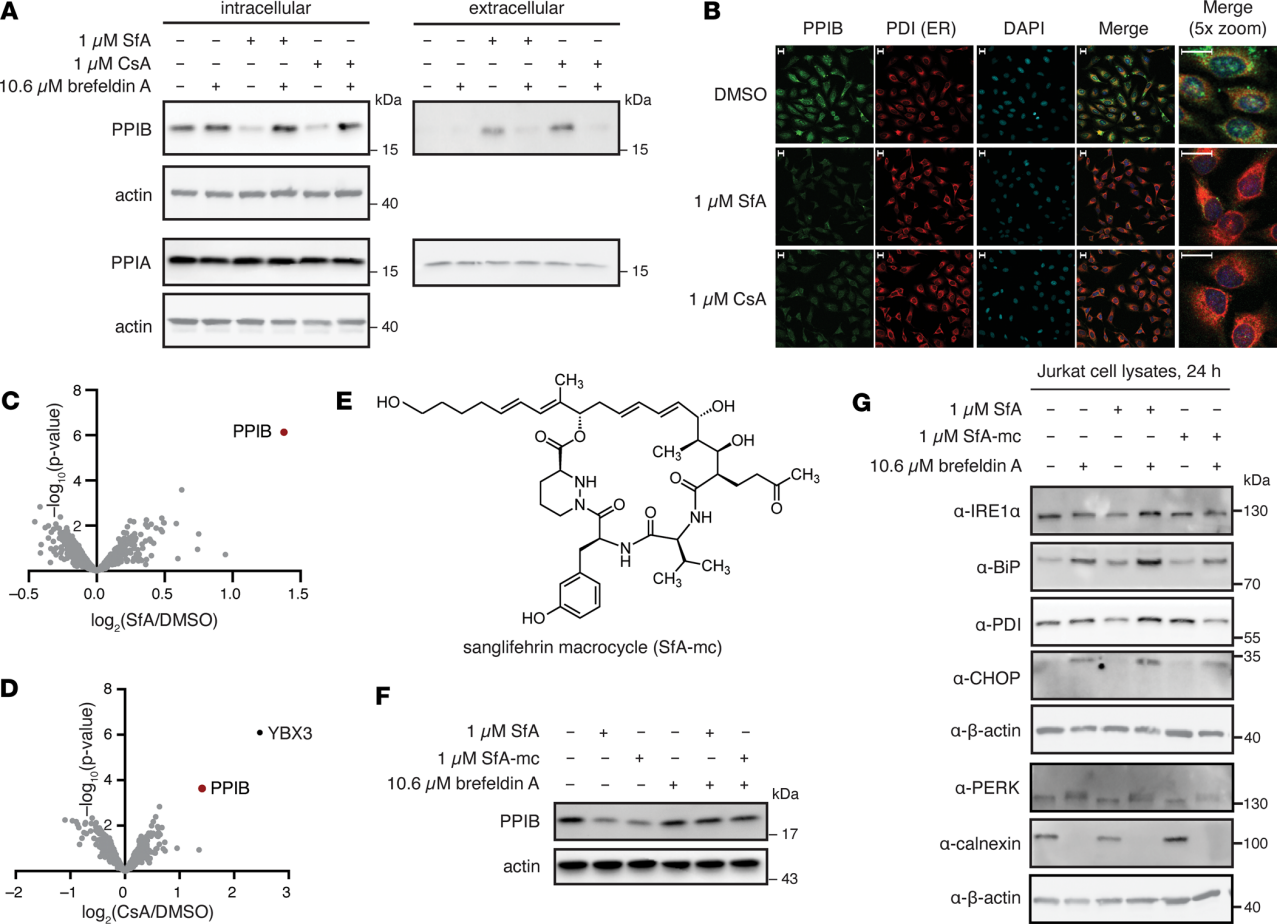
SfA induces secretion of PPIB from cells. (**A**) Western blot of intracellular and extracellular PPIB in Jurkat cells treated with 1 μM SfA or 1 μM CsA for 4 hours ± 10.6 μM brefeldin A. (**B**) Immunofluorescence imaging of PPIB and PDI in HeLa cells treated with 1 μM SfA or 1 μM CsA for 4 hours. Scale bars: 20 μm. (**C**) Volcano plot of proteins secreted by Jurkat cells following treatment with 1 μM SfA for 4 hours relative to vehicle (*n* = 3). (**D**) Volcano plot of proteins secreted by Jurkat cells following treatment with 1 μM CsA for 4 hours relative to vehicle (*n* = 3). (**E**) Structure of sanglifehrin macrocycle (SfA-mc). (**F**) Western blot of intracellular PPIB in Jurkat cells treated with 1 μM SfA or 1 μM SfA-mc for 4 hours ± 10.6 μM brefeldin A. (**G**) Western blot of Jurkat cell lysates for ER stress markers after treatment with 1 μM SfA or 1 μM SfA-mc with or without brefeldin A for 24 hours (*n* = 3).

PPIB is secreted upon CsA binding to its catalytic domain (31). Since SfA similarly binds to PPIB in live cells, we next sought to assess the effects of SfA on PPIB secretion. Notably, treatment of Jurkat cells with 1 μM SfA produced a decrease in intracellular PPIB levels, but not to PPIA, and an associated increase in extracellular PPIB in the conditioned media within 4 hours (Figure 2A and Supplemental Figure 7A). CsA treatment had a comparable effect on PPIB secretion under these conditions. SfA-induced PPIB secretion was inhibited by ER transport inhibitors such as brefeldin A but not by inhibitors of other pathways, such as MLN4924 for inhibition of neddylation, MG132 for inhibition of proteasomal degradation, E64d or pepstatin for inhibition of lysosomal degradation, or cycloheximide for inhibition of protein synthesis (Figure 2A and Supplemental Figure 7B). These effects were confirmed by fluorescence microscopy for PPIB and the ER marker protein disulfide isomerase (PDI) in HeLa cells, analogous to previous studies with CsA (Figure 2B) (31). MS analysis of secreted proteins validated the increase in extracellular PPIB levels upon SfA or CsA treatment (log_2_[fold change] > 1, *P* < 0.05; Figure 2, C and D, and Supplemental Table 2). These data indicate that the secretion of PPIB upon treatment with cyclophilin binders may result from disruption of native interactions in the ER.

To investigate the role of SfA on PPIB in mediating the immunosuppressive effects of SfA, we developed a SfA macrocycle (SfA-mc) functionalized with a primary alcohol (Figure 2E) for comparison with SfA. We hypothesized that SfA-mc would retain cyclophilin binding and be associated with induction of PPIB secretion but would differentiate from immunosuppressive activity, similar to prior efforts that have found that oxidative cleavage of SfA yields an SfA derivative that retains cyclophilin binding but has reduced immunosuppressive activity (25, 32). As expected, SfA-mc had a minimal effect on Jurkat and K562 viability but maintained similar binding affinity for PPIB with some loss of affinity for PPIB, relative to SfA (Table 1, Supplemental Figure 3, and Supplemental Figure 7, C and D). Excitingly, SfA and SfA-mc induced similar changes in intracellular PPIB levels following the treatment of Jurkat cells with 1 μM compound for 4 hours (Figure 2F). SfA and SfA-mc did not upregulate markers of ER stress after 24 hours, indicating that SfA activity on export of PPIB does not stimulate a broader stress-response signal in cells (Figure 2G). By contrast, brefeldin A treatment promoted strong upregulation of BiP, CHOP, and calnexin markers for ER stress. These data suggest that SfA activity through PPIB is independent of the immunosuppressive effects exerted by SfA on T cells and B cells and that these effects can be differentiated by SfA analogs.

### SfA inhibits collagen secretion induced by TGF-β1 in human lung fibroblasts.

**Figure 3:**
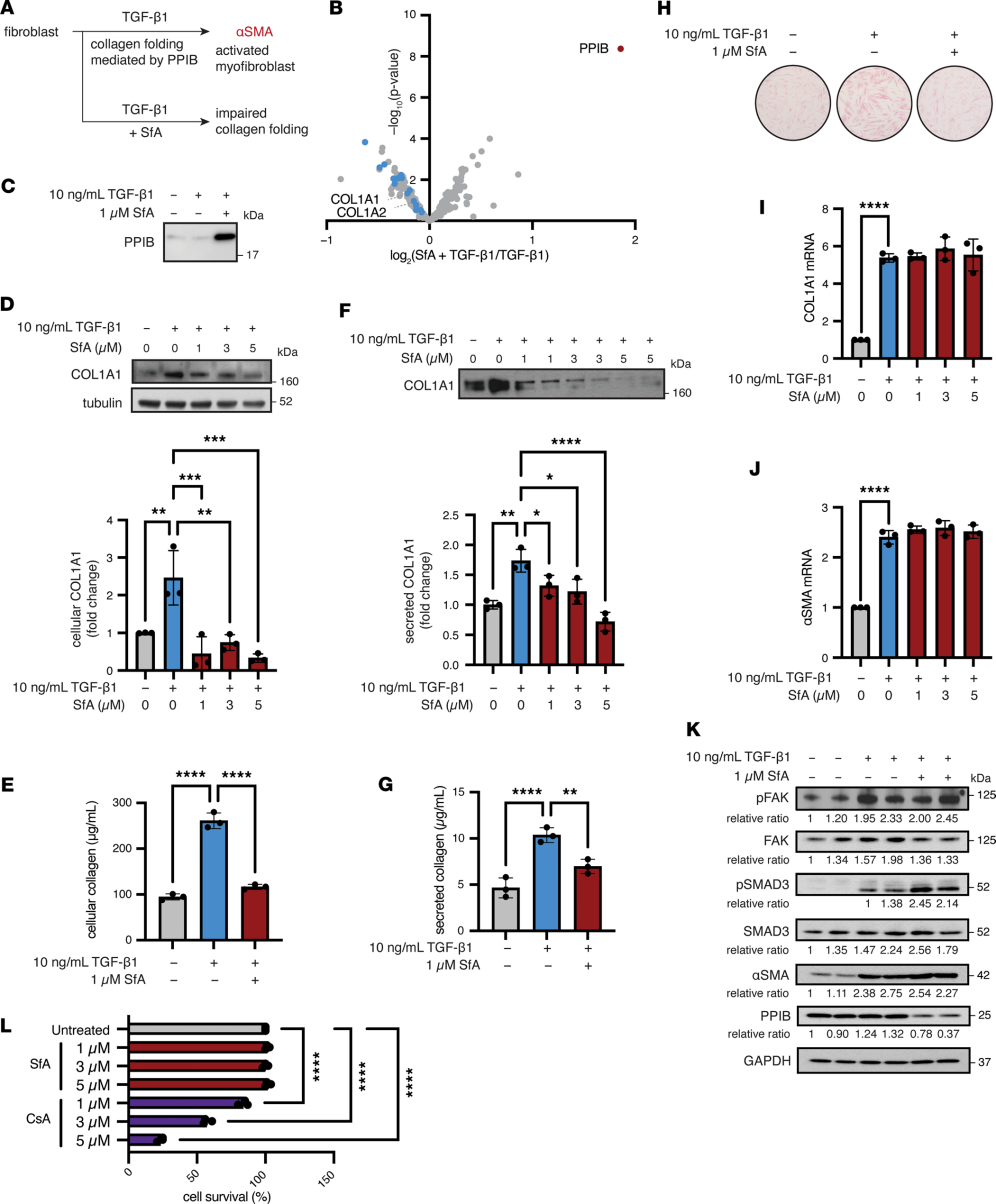
SfA reduces collagen production in an IMR-90 fibroblast model of fibrosis. (**A**) Schematic of proposed effects of SfA in myofibroblasts after TGF-β1 activation. (**B**) Secretomics of myofibroblasts treated with SfA. Collagens are highlighted in blue; PPIB is highlighted in red (*n* = 3). (**C**) Western blot for PPIB in conditioned media analyzed in **B**. (**D**) Western blot for intracellular COL1A1 following stimulation. (**E**) Sircol assay for intracellular collagen following stimulation. (**F**) Western blot for extracellular COL1A1 following stimulation. Conditioned media were derived from samples in **D**. (**G**) Sircol assay measuring extracellular collagen following stimulation. (**H**) Intracellular collagen visualized by sircol staining following stimulation. (**I**) Analysis of COL1A1 gene expression following stimulation. (**J**) Analysis of αSMA gene expression following stimulation. (**K**) Western blot and relative quantification for cellular proteins associated with myofibroblast activation following stimulation. (**L**) Survival as determined by trypan blue staining following treatment with the indicated compounds for 96 hours in serum-free media. Stimulation conditions: 10 ng/mL TGF-β1 ± 1 μM SfA for 96 hours (*n* = 3). Fold change was calculated by densitometry. All graphed data represent mean ± SD. Significance was determined by 1-way ANOVA followed by pairwise comparisons corrected for multiple comparisons using the Šidák correction. *P < 0.05, **P < 0.01, ***P < 0.001, ****P < 0.0001.

SfA-induced inhibition and secretion of PPIB is analogous to knockdown of PPIB. Given that PPIB is critical for collagen folding and its knockdown results in loss of collagen (33, 34), we next evaluated the effect of SfA in fibrosis, which is characterized by excessive production of collagen type I. PPIB catalyzes the rate-limiting step of procollagen triple helix folding within the collagen prolyl 3-hydroxylation complex, which is comprised of prolyl 3-hydroxylase 1 (P3H1), cartilage-associated protein (CRTAP), and PPIB (33–35). We thus reasoned that SfA might interfere with collagen type I synthesis by activated myofibroblasts and evaluated this mechanism in TGF-β1–activated IMR-90 human lung fibroblasts (Figure 3A). TGF-β1 is a potent profibrotic factor that induces fibroblast-to-myofibroblast transdifferentiation during tissue repair and fibrosis, a phenotype characterized by increased synthesis and deposition of collagen type I and other ECM proteins (36, 37). Unlike quiescent fibroblasts, myofibroblasts express α-smooth muscle actin (αSMA), a protein that confers a hypercontractile phenotype and allows myofibroblasts to remodel and stiffen the ECM (1, 38). MS profiling of the secretome of IMR-90 human lung fibroblasts stimulated with TGF-β1 in the presence or absence of SfA showed that SfA treatment reduced protein levels of several collagens in the supernatant of TGF-β1–treated fibroblasts (Figure 3B and Supplemental Table 3). SfA treatment also increased PPIB levels in fibroblast supernatants, further validating the induction of PPIB secretion by SfA (Figure 3, B and C, and Supplemental Table 3).

We next assessed collagen levels in cell lysates and supernatants by sircol assay and Western blot. SfA treatment significantly reduced both intracellular and extracellular collagen type I α 1 (COL1A1) in TGF-β1–treated fibroblasts in a dose-dependent manner (Figure 3, D–H). SfA did not affect COL1A1 mRNA levels, consistent with the notion that production of mature, folded collagen type I is inhibited by SfA (Figure 3I). SfA also forms a PPIA:SfA binary complex that regulates the cell cycle via IMPDH2 (25). To rule out that inhibition of the PPIA/SfA/IMPDH2 pathway by SfA is contributing to the reduction of collagen synthesis in fibroblasts, we tested SfA-mc, which does not form a PPIA/SfA/IMPDH2 complex. SfA-mc induces secretion of PPIB and possesses the same potency as SfA at blocking collagen type I secretion by fibroblasts (Supplemental Figure 8A). SfA inhibits collagen synthesis and secretion without affecting αSMA protein or mRNA levels in TGF-β1–activated myofibroblasts, indicating that SfA does not affect the contractility of these cells (Figure 3, J and K). Furthermore, profibrotic signaling pathways including SMAD2/3 and FAK signaling, which are strongly activated by TGF-β1 in myofibroblasts (39, 40), are also unaffected by SfA (Figure 3K). SfA did not affect in vitro cellular responses in a wound-healing scratch assay (Supplemental Figure 8B). These results demonstrate the specific activity of SfA, which reduces collagen type I protein levels without affecting upstream profibrotic pathways. Notably, SfA treatment also reduced intracellular PPIB protein levels in TGF-β1–activated myofibroblasts (Figure 3K). Moreover, SfA or SfA-mc treatment did not affect myofibroblast survival, in contrast to the toxicity observed in response to CsA treatment (Figure 3L and Supplemental Figure 8C). Taken together, our data show that SfA induces PPIB secretion in myofibroblasts with an associated significant reduction in collagen type I levels in vitro.

### SfA reduces established skin fibrosis in the bleomycin mouse model.

**Figure 4:**
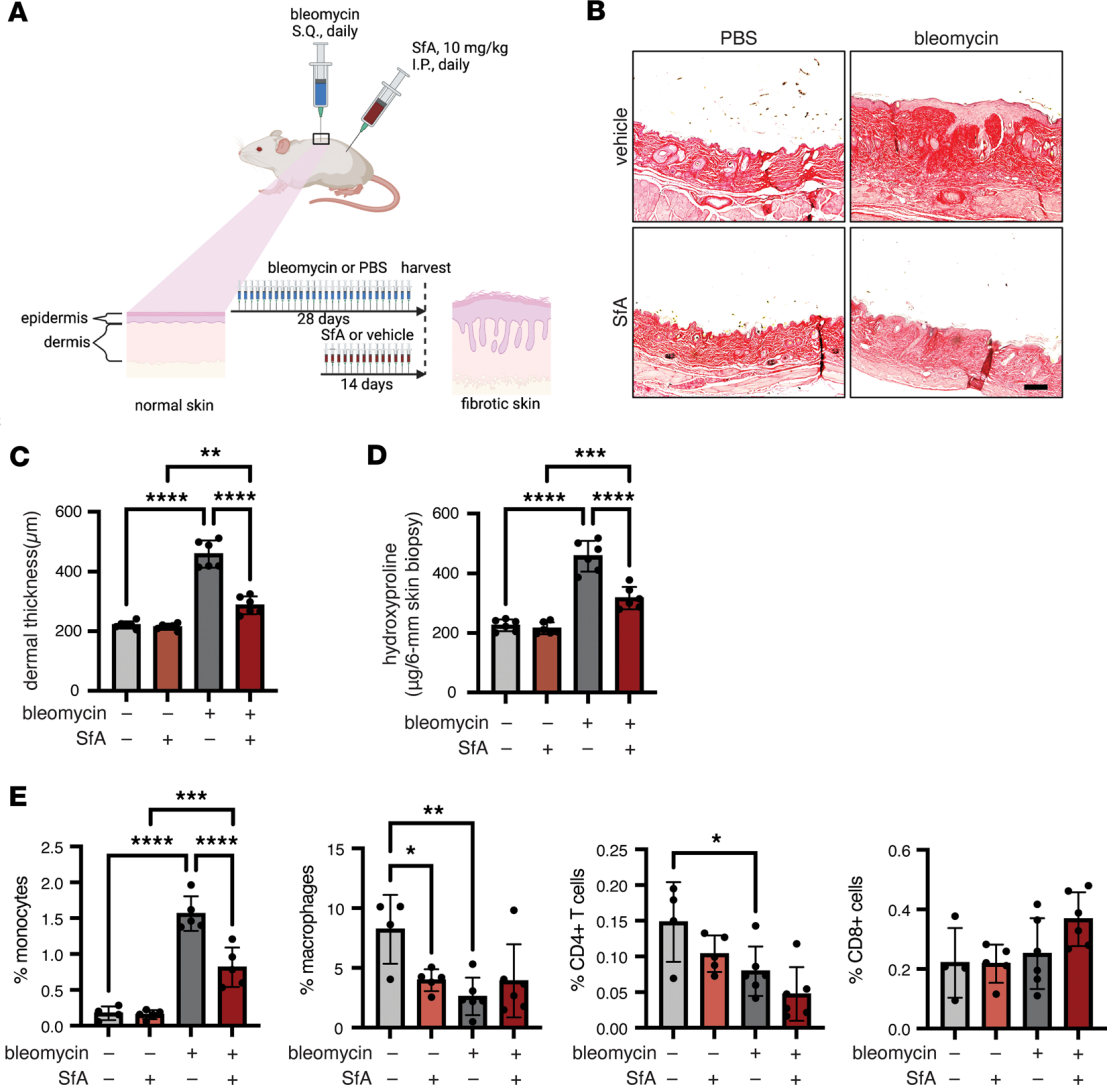
SfA reduces fibrosis and immune activation in a mouse model of bleomycin-induced skin fibrosis. (**A**) Schematic of experimental procedure. (**B**) Representative images of skin sections stained with Picrosirius red to visualize collagen. Scale bar: 100 μm. (**C**) Dermal thickness, as determined by measuring distance between the epidermal-dermal junction and the dermal-fat junction (*n* = 6). (**D**) Collagen content in skin-punch samples, as determined by hydroxyproline assay (*n* = 6). (**E**) Characterization of immune cells in skin biopsy samples. All graphed data represent mean ± SD (*n* = 4–6). **C** and **D** were analyzed using a 2-way ANOVA followed by pairwise comparisons corrected for multiple comparisons using the Tukey correction. **E** was analyzed using 1-way ANOVA followed by pairwise comparisons corrected for multiple comparisons using the Šidák correction. *P < 0.05, **P < 0.01, ***P < 0.001, ****P < 0.0001.

Our in vitro studies show that SfA inhibited and induced secretion of PPIB, which is associated with inhibition of collagen folding and synthesis in myofibroblasts, providing a strong rationale for testing the antifibrotic effects of SfA in vivo. We opted for the well-established bleomycin-induced skin and lung fibrosis mouse model, which is widely used to study the biology of myofibroblasts and to test the efficacy of antifibrotic drugs in vivo (41, 42). Using this model, we examined the therapeutic potential of SfA to treat established skin and lung fibrosis when administered therapeutically from day 14 to 28 after the onset of daily bleomycin challenges (Figure 4A). Therapeutic administration of SfA (10 mg/kg daily) significantly reduced bleomycin-induced skin fibrosis when compared with vehicle control at day 28 after bleomycin challenge, as assessed by Picrosirius red staining of the skin for collagen, measurement of skin dermal thickness, and skin hydroxyproline content, a biochemical proxy for collagen deposition (Figure 4, B–D). The bleomycin-induced increase in dermal thickness was reduced by 78% in bleomycin-challenged, SfA-treated mice compared with bleomycin-challenged, vehicle-treated mice. Additionally, the bleomycin-induced increase in skin hydroxyproline was reduced by 58% with SfA treatment, demonstrating potent antifibrotic effects of SfA in vivo. Immune cells may also promote fibrosis in this model by stimulating myofibroblast activation via secretion of profibrotic mediators including TGF-β1 (41, 43). To investigate the antiinflammatory effects of SfA, we performed flow cytometry analysis of skin tissue from mice treated with or without SfA after saline or bleomycin challenge (Figure 4E and Supplemental Figure 9A). Our results demonstrate that SfA treatment significantly reduced the percentage of inflammatory monocytes in the skin of bleomycin-challenged SfA-treated mice compared with bleomycin-challenged vehicle-treated mice. There was no significant difference in the percentage of macrophages, CD4^+^ T cells, or CD8^+^ T cells that were present in the skin of bleomycin-challenged SfA-treated mice compared with bleomycin-challenged vehicle-treated mice (Figure 4E). Together, these results suggest that SfA is beneficial in treating established skin fibrosis by reducing collagen levels and inhibiting monocyte infiltration.

### SfA reduces established lung fibrosis in the bleomycin mouse model.

**Figure 5:**
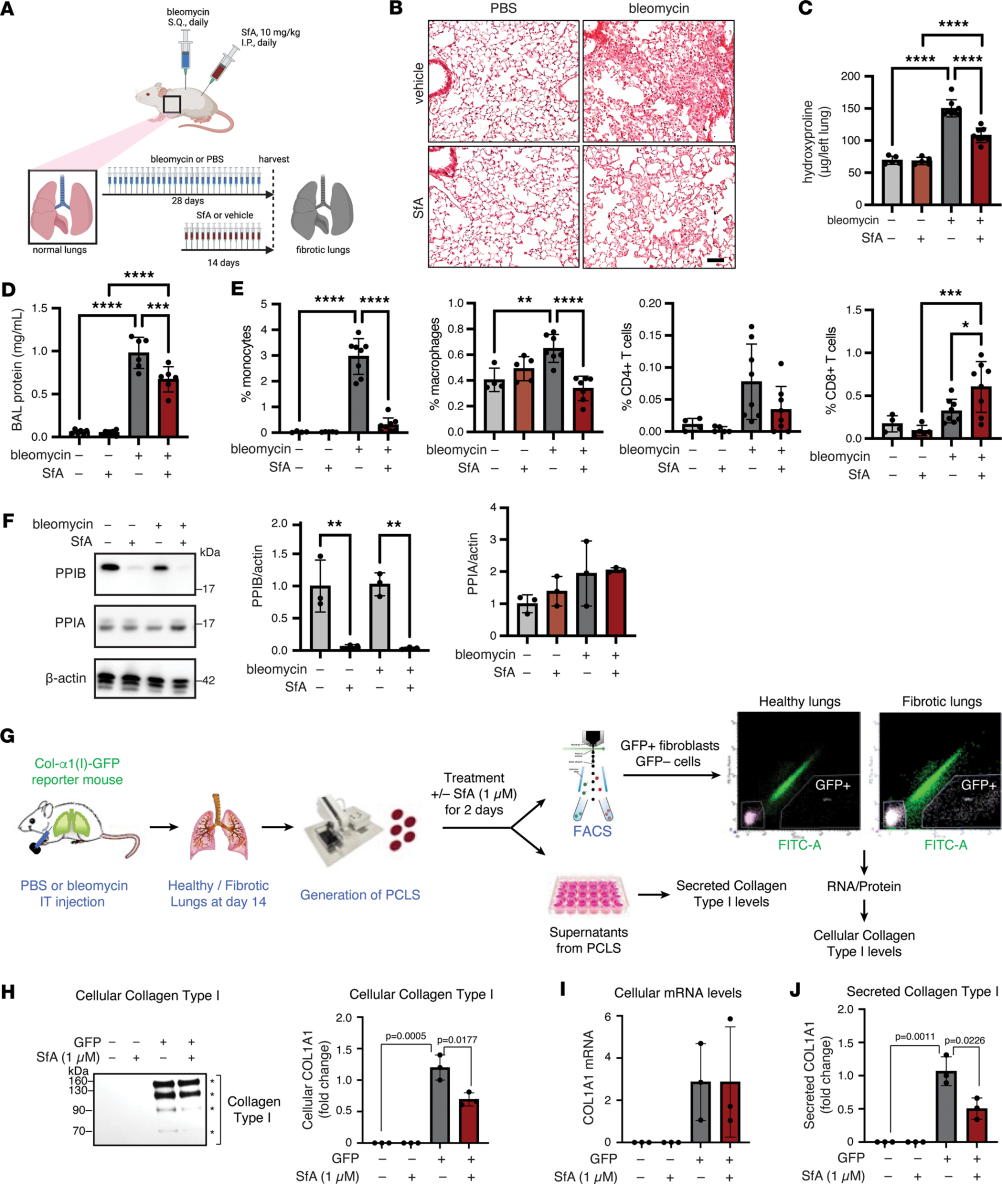
SfA reduces fibrosis and immune activation in a mouse model of bleomycin-induced lung fibrosis. (**A**) Schematic of experimental procedure. (**B**) Representative images of lung sections stained with Picrosirius red to visualize collagen. Scale bar: 100 μm. (**C**) Collagen content in left lungs, as determined by hydroxyproline assay (*n* = 5–8). (**D**) Vascular leak assay, as determined by BCA assay for protein content in bronchioalveolar lavage (BAL) supernatant (*n* = 6). (**E**) Characterization of immune cells in BAL (*n* = 4–8). (**F**) Western blot for PPIA and PPIB in lung cell lysates. Representative Western blot and quantification of PPIA and PPIB in lung cell lysates (*n* = 3). (**G**) Generation of fibrotic precision cut lung slices (PCLS) from transgenic collagen-GFP reporter mice (Col-GFP) at day 14 after bleomycin challenge. PCLS were treated with or without SfA (1 μm) for 2 days (*n* = 3). (**H** and **I**) Collagen type I protein and mRNA levels were assessed in GFP^–^ cells and GFP^+^ fibroblasts sorted by FACS from PCLS by Western blot (**H**) and real time PCR (**I**), respectively (*n* = 3). Secreted collagen type I was assessed in PCLS supernatants by sircol assay (**J**) (*n* = 3). All graphed data represent mean ± SD. **C**, **D**, **F**, and **H**–**J** were analyzed using 1-way ANOVA followed by pairwise comparisons corrected for multiple comparisons using the Šidák correction. **E** was analyzed using a 2-way ANOVA followed by pairwise comparisons corrected for multiple comparisons using the Tukey correction. *P < 0.05, **P < 0.01, ***P < 0.001, ****P < 0.0001.

Since s.c. injection of bleomycin leads to concomitant pulmonary fibrosis in mice (Figure 5A) (44), we next examined the lungs of mice treated with or without therapeutic SfA at the same time points used for the study of skin fibrosis. Therapeutic administration of SfA significantly reduced histological measures of fibrosis in the lungs when compared with vehicle control at day 28 after bleomycin challenge, as assessed by Picrosirius red staining (Figure 5B). In addition, hydroxyproline levels in lung tissue, indicative of collagen type I content, were reduced in bleomycin-challenged SfA-treated mice compared with bleomycin-challenged vehicle-treated mice (Figure 5C). Notably, SfA treatment also reduced the increased alveolar–capillary barrier permeability induced after bleomycin challenge, as determined by reduction in total protein levels in the bronchoalveolar lavage (BAL) fluid (Figure 5D). In addition, SfA treatment also reduced the number of inflammatory monocytes and macrophages in the BAL induced by bleomycin injury, validating the antiinflammatory effects of SfA (Figure 5E and Supplemental Figure 9B). We further leveraged multiplexed immunofluorescence to demonstrate that SfA treatment decreased the number of profibrotic macrophages (Supplemental Figure 10). Of note, SfA did not affect the percentage of CD4^+^ T cells, although it did increase the number of CD8^+^ T cells in fibrotic lungs. SfA treatment resulted in near-complete loss of PPIB from lung tissue with no changes in PPIA levels (Figure 5F). These results indicate that SfA treats established lung fibrosis by reducing collagen levels and inhibiting monocyte infiltration; SfA treatment was also associated with reduced tissue PPIB levels. We next generated fibrotic precision-cut lung slices (PCLS) from transgenic collagen-GFP reporter mice (Col-GFP) subjected to our bleomycin lung fibrosis model (Figure 5G). In this 3D ex vivo model of lung fibrosis, thin slices freshly prepared from fibrotic tissues represent a powerful tool to study fibrosis mechanism and testing antifibrotic responses to drug compounds (45, 46). Our results indicate that treatment of murine fibrotic PCLS with SfA for 2 days reduced collagen type I levels in GFP^+^ fibrotic fibroblasts directly isolated by FACS from PCLS (Figure 5, G and H). Of note, COL1A1 mRNA levels were not modulated by SfA treatment (Figure 5I), indicating that SfA prevents collagen synthesis without affecting collagen mRNA transcription. In addition, SfA treatment resulted in reduced soluble collagen type I secretion from PCLS, as assessed in PCLS-derived supernatant (Figure 5J). Taken together, our in vivo data support that SfA modulates PPIB, which is a therapeutic target for the treatment of skin and lung fibrosis.

### SfA inhibits collagen type I secretion from lung tissue of patients with IPF.

**Figure 6:**
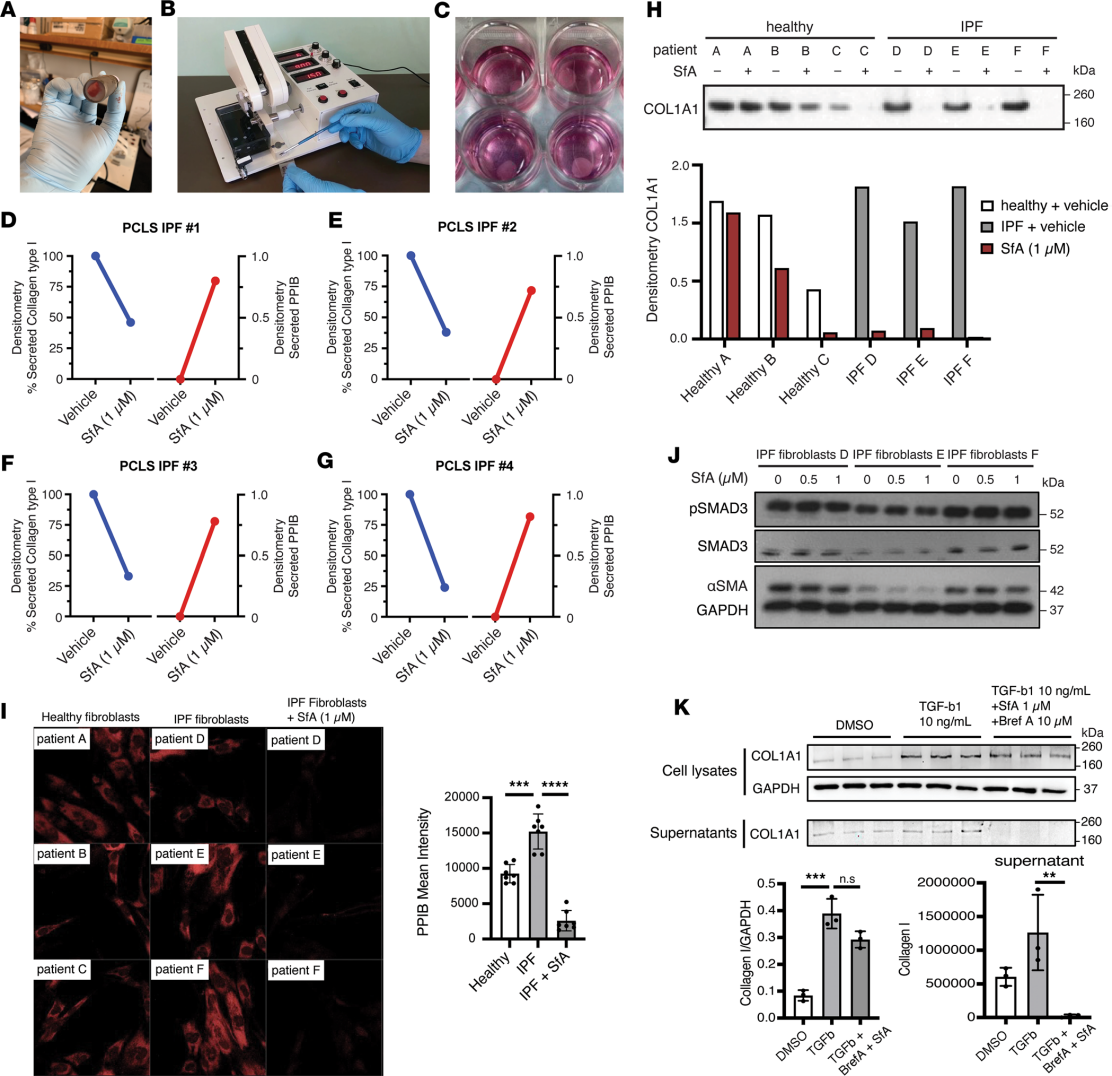
SfA reduces collagen secretion from primary fibrotic fibroblasts. (**A** and **B**) Generation of precision cut lung slices (PCLS) from explanted lung tissue isolated from patients with idiopathic pulmonary fibrosis (IPF). Lung tissue was obtained in 1 cm blocks (**A**) before being sliced (200–300 μm thick) using a Compresstome VF-310-0Z (**B**). (**C**) PCLS were then treated with or without SfA (1 μM) for 72 hours in culture (*n* = 4) . (**D**–**G**) Analysis of collagen and PPIB secretion from PCLS prepared from IPF patient lung tissue ± 1 μM SfA in supernatants (*n* = 4). (**H**) Western blot for collagen type I secreted by primary human fibroblasts isolated from healthy controls and patients with IPF ± 1 μM SfA over 96 hours in cell supernatants. (**I**) Intracellular PPIB levels in healthy or IPF fibroblasts with or without 1 μM SfA treatment by immunofluorescence. (**J**) Western blot for α-smooth muscle actin (αSMA) and phosphoSMAD3/SMAD3 signaling in IPF fibroblasts ± SfA over 96 hours in cell lysates. GAPDH was used as loading control. (**K**) COL1A1 levels in cell lysates and supernatants of TGF-β–activated fibroblasts treated with or without 1 μM SfA and 10 μM brefeldin A. **D**–**G** were analyzed with a 2-tailed *t* test against a hypothetical value of 1. Graphed data in **I** and **K** represent mean ± SD.

To determine the relevance of this mechanism in human disease, we assessed collagen type I secretion from PCLS prepared from explanted lung tissue from patients with IPF (*n* = 4 individual patients; *n* ≥ 3 slices per patient/treatment) treated with SfA (1 μM) or vehicle for 3 days (Figure 6, A–C). Consistent with results observed in vitro and in the preclinical mouse model, SfA treatment reduced collagen type I protein secretion compared with vehicle control, an effect associated with PPIB secretion (Figure 6, D–G). To investigate antifibrotic effects of SfA in IPF, we next assessed the effects of SfA on primary human fibroblasts isolated from patients with IPF (*n* = 3) and healthy controls (*n* = 3). The fibrotic fibroblasts isolated from patients with IPF secreted slightly higher levels of collagen type I into culture medium and showed upregulation of PPIB compared with normal lung fibroblasts in vitro using our cultured conditions (Figure 6, H and I). Treatment with SfA (1 μM) significantly reduced collagen type I levels in the supernatant of IPF fibroblasts and induced secretion of intracellular PPIB, while treatment of healthy control fibroblasts with SfA did not have a significant effect on collagen type I levels (Figure 6, H and I). SfA (1 μM) treatment did not modulate IPF fibroblast contractility — assessed by αSMA protein levels — or TGF-β1/SMAD signaling (Figure 6, J and K, and Supplemental Figure 11). Taken together, these studies support a mechanism through which SfA inhibits collagen type I secretion through PPIB in IPF fibroblasts without affecting other fibroblast functions or fibrogenic TGF-β1 signaling.

## Discussion

We applied PAL and chemical proteomics to connect PPIB, an ER-resident chaperone involved in collagen type I folding and maturation, as a mechanistic target for SfA in fibrosis. Previous studies have connected cyclophilin inhibitors or SfA analog NV556 to inhibition of liver fibrosis (47, 48), although the specific cyclophilin and precise cellular mechanism were not further evaluated. In addition, separate studies with SfA have demonstrated inhibition of multiple members of the cyclophilin family and made specific mechanistic connections through PPIA (25) and PPIF (49) or were agnostic to the cyclophilin isoform (22–24, 29). In vivo studies with SfA in mouse models of disease have demonstrated antiinflammatory activities and prevention of epithelial cell damaged via modulation of the mitochondrial permeability transition pore, which were connected to inhibition of PPIA or PPIF in vivo. While these activities have been associated with inhibition of fibrosis, direct antifibrotic effects of SfA have not been demonstrated in vivo. Our results now demonstrate that treatment of cells with SfA induces the secretion of PPIB into the extracellular space in multiple cell lines and primary fibroblasts, which affects collagen synthesis given the importance of PPIB in collagen type I folding and maturation. Our in vitro studies demonstrate that SfA targets PPIB and promotes its secretion into the extracellular space, resulting in depletion of intracellular PPIB and reduction in collagen type I levels in TGF-β1–activated myofibroblasts. Additional studies to separate the inhibition and secretion of PPIB that appear to occur simultaneously on treatment with SfA and characterize the effect on collagen processing will fully illuminate the contribution of each step. Nonetheless, protein regulation as a therapeutic approach has been achieved by inhibiting protein expression with siRNA and by targeted protein degradation and is here suggested by a third mechanism of secretion.

These results translated to a mouse model of bleomycin-induced skin and lung fibrosis, where therapeutic SfA treatment resulted in reduced fibrosis, as determined by histological and biochemical measures. Importantly, PPIB levels in lung tissue were drastically reduced with SfA treatment, supporting in vivo translation of the mechanism observed in vitro. SfA additionally dampened the innate immune response, as indicated by reduced monocyte infiltration, suggesting a dual mechanism acting both on collagen maturation and separately on the innate immune response. Furthermore, in PCLS and fibroblasts isolated from patients with IPF, SfA treatment reduced collagen secretion, suggesting that the effects observed in the mouse model are translatable to IPF, a human disease sorely in need of additional treatments.

From a translational perspective, an important future direction is to evaluate whether nonimmunosuppressive SfA and CsA analogs (50) are preferable for antifibrotic applications. Our data show that SfA has both antiinflammatory and antifibrotic effects in a preclinical mouse model. These effects may be separable. Although limited quantities of SfA-mc prevented further exploration of the separation of these effects, therapeutic targeting of cyclophilins by nonimmunosuppressive analogs derived from SfA and CsA, previously developed as antivirals (50), has been shown to have antifibrotic effects in the CCl_4_ model of liver fibrosis and in a mouse model of nonalcoholic steatohepatitis (NASH) (47, 48, 51). Comparison of SfA and SfA-mc, which similarly inhibit and induce PPIB secretion, indicates that targeting PPIB may be separable from the immunosuppressive effects of SfA, and further structure-activity relationship studies may yield optimized molecules for treatment of fibrosis.

Additionally, SfA is a pancyclophilin inhibitor, and therefore a role for other PPIases in the overall response to SfA cannot be completely excluded without the development of more selective PPIB ligands. Nonetheless, this mechanism is partly supported by genetic studies demonstrating reduced collagen type I crosslinking in PPIB-deficient mice, which also develop features of osteogenesis imperfecta (33, 34, 52, 53). Notably, though excessive TGF-β1 signaling is a common mechanism in these mouse models of osteogenesis imperfecta, these mice do not develop organ fibrosis (54), consistent with the notion that PPIB may be required for TGF-β1 signaling to initiate the development of fibrosis. By contrast, studies with the SfA-derived pancyclophilin inhibitor GS-642362, which targets PPIA, PPIB, and PPIF, in the unilateral ureteric obstruction (UUO) mouse model showed inhibition of renal fibrosis by preventing tubular epithelial cell death and neutrophil infiltration (55). PPIB-deficient mice are viable and partially protected from inflammation in the UUO model at day 7, although fibrosis was not assessed at later time points (56), while efforts with PPIA-deficient mice show reduced inflammation in the bilateral renal ischemia/reperfusion injury (IRI) model but are not protected from renal fibrosis in the UUO model, suggesting that PPIA regulates inflammation but not fibrosis (57). Furthermore, PPIF-deficient mice showed protection from renal fibrosis in the UUO model due to reduction in tubular epithelial cell apoptosis (58), and TGF-β1–induced collagen type I expression in fibroblasts isolated from these mice was comparable with that observed in WT fibroblasts, suggesting that PPIF does not play a direct role in fibrogenic responses. Further studies aided by the development of more selective PPIB ligands are needed to elucidate the molecular underpinnings linking PPIB, TGF-β1, and organ fibrosis in vivo, such as via a strategy to generate isoform-selective cyclophilin inhibitors by engineering macrocycle scaffolds (59). Our studies indicate that PPIB is an antifibrotic target and motivates future optimization of compounds to selectively target it.

In summary, our work shows that the natural product SfA exerts antifibrotic effects by targeting PPIB, interfering with collagen maturation, and reversing bleomycin-induced skin and lung fibrosis in a mouse model. This mechanism represents a strategy for treating organ fibrosis by interfering with collagen type I maturation in myofibroblasts. We uncovered this mechanism through profiling of SfA by chemical proteomics, which demonstrated that PPIB is a major cellular target of SfA. Incorporation of compounds acting through PPIB into the currently paltry arsenal of antifibrotic therapeutics has the potential to usher in an era of improved outcomes for patients suffering from fibrosis.

## Methods

### Sex as a biological variable.

Sex was not considered as a biological variable.

### Study design.

Throughout this study, replicates represent measurements of distinct samples.

Initial in vitro studies were performed to map the interactome of SfA in Jurkat and K562 cells in an unbiased manner by PAL and proteomics. Based on the results of this analysis, we hypothesized that SfA might have similar effects on PPIB secretion compared with those previously reported for CsA; experiments were initiated to test this hypothesis. Since experiments supported this hypothesis, we evaluated the role of induced PPIB secretion in the potency of SfA by employing an SfA analog that we expected to maintain cyclophilin binding and PPIB secretion but have reduced potency; these hypotheses were supported by our data. Given literature discussion of the role of PPIB in collagen production, we moved to test whether SfA reduced collagen secretion in a myofibroblast model of fibrosis. To distinguish a PPIB secretion–dependent mechanism from a mechanism targeting upstream profibrotic pathways, we evaluated intracellular and extracellular PPIB levels and markers of upstream profibrotic pathways. For in vitro studies, experiments were routinely performed in biological duplicate or triplicate. Cell viability (MTT) assay data were fit to a 4-parameter logistic (4PL) regression to exclude outliers with Q = 1, but no outliers were identified in the presented data. Unless otherwise noted, data sets obtained from Western blots and sircol assays were evaluated with 1-way ANOVA followed by multiple comparisons across –TGF-β1/–SfA versus +TGF-β1/–SfA and +TGF-β1/–SfA versus +TGF-β1/+SfA (repeated for each SfA concentration if multiple were tested) with a Šidák correction. Data from the cell viability assay were evaluated with 1-way ANOVA followed by multiple comparisons across control versus each SfA/CsA concentration tested with a Šidák correction.

Based on the results of our in vitro studies, we hypothesized that SfA would be beneficial in a mouse model of bleomycin-induced skin and lung fibrosis. This study was conducted with predetermined sample sizes and scheduling. Analyses conducted included routine histology and biochemical assays to measure fibrosis and flow cytometry profiling of immune cell populations. Additional analyses of PPIB levels in BAL fluid and lung tissue were also conducted to evaluate the proposed mechanism of SfA action. Data sets with equal numbers of points for each condition were evaluated using a 2-way ANOVA followed by multiple comparisons with Tukey correction. All combinations were tested for significance, but only significant differences among–bleomycin/–SfA versus +bleomycin/–SfA, –bleomycin/–SfA versus –bleomycin/+SfA, +bleomycin/–SfA versus +bleomycin/+SfA, and –bleomycin/+SfA versus +bleomycin/+SfA are plotted. Data sets with unequal numbers of points across sets were evaluated with a 1-way ANOVA followed by multiple comparisons across –bleomycin/–SfA versus +bleomycin/–SfA, –bleomycin/–SfA versus–bleomycin/+SfA, +bleomycin/–SfA versus +bleomycin/+SfA, and –bleomycin/+SfA versus +bleomycin/+SfA with a Šidák correction. Given the promising results obtained in the mouse study, we proceeded to obtain PCLS from patients with IPF and samples of fibroblasts isolated from healthy patients and patients with IPF (3 patients per group), which were cultured to test the effect of SfA on secreted collagen. The ratio of collagen secreted with SfA versus without SfA was calculated for each fibroblast sample. These data were evaluated using a 2-tailed *t* test against a hypothetical value of 1.

### Statistics.

Data were analyzed in GraphPad Prism 9 using the tests indicated. A *P* < 0.05 was considered significant.

### Study approval.

All animal experiments were performed in accordance with *Guide for the Care and Use of Laboratory Animals* (National Academies Press, 2011) and protocols approved by the Massachusetts General Hospital Subcommittee on Research Animal Care. All mice were maintained in a specific pathogen–free (SPF) environment certified by the American Association for Accreditation of Laboratory Animal Care (AAALAC).

All human experiments were performed under protocols approved by the Institutional Ethics Committee approved by the Massachusetts General Hospital. Patients with IPF were identified from those receiving care at the Massachusetts General Hospital. For study inclusion, patients with IPF had to satisfy IPF diagnostic criteria based on the 2011 joint consensus statement of the American Thoracic Society (ATS), European Respiratory Society (ERS), Japanese Respiratory Society, and Latin American Thoracic Association as determined by 2 investigators.

### Data availability.

Detailed methods and synthetic procedures are provided in the Supplemental Methods. Proteomics data have been deposited in the PRIDE repository under identifiers PXD029540, PXD029541, and PXD031010. Values associated with the main manuscript and supplement material are found in the Supporting Data Values file and Supplemental Tables 1–3.

## Author contributions

HAF, MC, HH, FK, RTL, RY, AW, RS, AEZ, and APS performed experiments and analyzed data. CC synthesized compounds. KEB provided samples of primary tissue. CMW and DL supervised the study. HAF, CMW, and DL wrote the manuscript with input from other authors.

## Supplementary Material

Supplemental data

Unedited blot and gel images

Supplemental tables 1-3

Supporting data values

## Figures and Tables

**Table 1:**
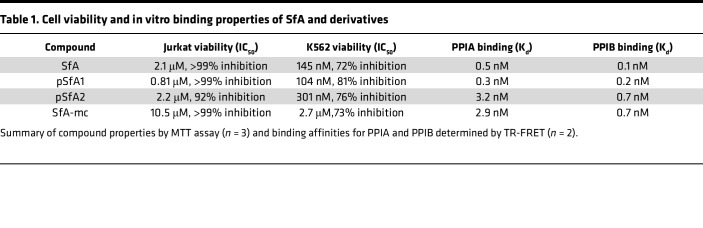
Cell viability and in vitro binding properties of SfA and derivatives

## Version 1 06/20/2024

In-Press Preview

## Version 2 08/08/2024

Electronic publication
