# Supplementary material for: Sanglifehrin A mitigates multiorgan fibrosis by targeting the collagen chaperone cyclophilin B
Source: JCI Insight. 2024 Jun 20;9(15):e171162. doi: 10.1172/jci.insight.171162 (PMC11383833; doi:10.1172/jci.insight.171162)
Supplement: Supplemental data [file jciinsight-9-171162-s122.pdf]

Supplementary Information for

Sanglifehrin A mitigates multi-organ fibrosis by targeting the collagen chaperone cyclophilin B

Hope A. Flaxman[1], Maria-Anna Chrysovergi[2], Hongwei Han[2], Farah Kabir[1], Rachael T. Lister[2], Chia-Fu Chang[1], Robert Yvon,[1] Katharine E. Black[2], Andreas Weigert[3,4], Rajkumar Savai[4,5,6,7], Alejandro Egea-Zorrilla [6,7], Ana Pardo-Saganta [6,7], David Lagares[2,*], Christina M. Woo[1,*]

[1] Department of Chemistry and Chemical Biology, Harvard University, Cambridge, MA

[2] Fibrosis Research Center, Center for Immunology and Inflammatory Diseases, Division of Pulmonary and Critical Care Medicine, Massachusetts General Hospital, Harvard Medical School, Boston, MA, USA

[3] Goethe-University Frankfurt, Faculty of Medicine, Institute of Biochemistry I, Germany

[4] Frankfurt Cancer Institute (FCI), Goethe University, and German Cancer Consortium (DKTK), Germany

[5] Max Planck Institute for Heart and Lung Research, Member of the German Center for Lung Research (DZL), Bad Nauheim, 61231, Germany.

[6] Institute for Lung Health (ILH), Department of Internal Medicine, Justus-Liebig University, Universities of Giessen and Marburg Lung Center (UGMLC), German Center for Lung Research (DZL), 35392 Giessen, Germany.

[7] Cardio-Pulmonary Institute (CPI), Department of Internal Medicine, Justus Liebig University, 35392 Giessen, Germany.

* Corresponding authors. Emails: Christina M. Woo, 12 Oxford St, Cambridge, MA 02138, 617-495-3858, cwoo@chemistry.harvard.edu; David Lagares, 857-264-8078, davidlagares@sloan.mit.edu

Chemical materials

Commercial solvents and reagents were used as received with the following exceptions. Toluene ($PhCH_3$), dichloromethane (DCM), dimethylformamide (DMF), diethyl ether (ether) and tetrahydrofuran (THF) were purified according to the method of Pangborn and co-workers.(1) Stabilizer-free solvents were prepared by distillation and stored with a compatible drying agent. $PhCH_3$, DCM, THF, and ether were stored over calcium hydride (CaH), while DMF was stored over activated 4Å molecular sieve (MS). *N,N*-diisopropylethylamine (DIPEA) was distilled over CaH under an atmosphere of nitrogen and stored over a pinch of CaH (100 mg/L) shielded from light. Anhydrous acetonitrile (CH_3CN) was prepared by allowing the solvents (100 mL) to sit with 4Å MS (5 g) for 24 h before use.

Biological materials

Plasmids

pGEX 2TK-PPIA and pcDNA3.1-PPIB were obtained from Genscript. pET41a was a gift from Emily Balskus (Harvard University, Cambridge, MA).

Bacterial cell lines

Rosetta 2 (DE3) cells were obtained from EMD Millipore (#71397-3). Mach1 cells were obtained from ThermoFisher (#C862003). NEB Stable Competent E. coli (High Efficiency) were obtained from New England Biolabs (NEB, #C3040I). Competent cells were prepared in-house with Mix & Go! E coli Transformation Buffer Set from Zymo Research (#T3002).

Cloning supplies

Primers were obtained from Sigma Aldrich. Restriction enzymes Xho1 and Spe1 were obtained from NEB (#R0146S and #R0133S). The quick ligation kit was obtained from NEB (#M2200S). T4 ligation buffer packaged with T4 DNA ligase was obtained from NEB (#M0202L). T4 PNK was obtained from

NEB (#M0236S). Plasmid DNA was prepared with the QuickDNA MiniPrep kit (Zymo, #D4068) and the ZymoPURE II Plasmid Midiprep Kit (Zymo, #D4201).

Mammalian cell lines

Jurkat, K562, and HeLa cells were a gift from the Bertozzi lab (Stanford University, Palo Alto, CA). A549 cells were obtained from ATCC (#CCL-185). IMR-90 cells were obtained from ATCC (#CCL-186).

Tissue culture

RPMI 1640 with *L*-glutamine was obtained from Corning (#10-040-CV). DMEM with glucose and *L*-glutamine was obtained from Lonza (#12-604-F). Pen/Strep 100× solution was obtained from VWR (#97063-708). Fetal bovine serum was obtained from Peak Serum (#PS-FB2). Methylthialazole Tetrazolium (MTT) was obtained from Cayman Chemical (#21795). Regular mycoplasma testing was performed using the MycoAlert PLUS detection kit (Lonza, #LT07-703). Tryphan blue 0.4% solution was obtained from VWR (#97063-702). DMEM with 4.5 g/L glucose without *L*-glutamine, with phenol red was obtained from Lonza (#12-614F). *L*-ascorbic acid was obtained from Sigma Aldrich (#A4403).

Antibodies

PPIA rabbit mAb was obtained from Cell Signaling Technologies (CST, #51418S — though this antibody is listed recognizing mouse PPIA, we observed that it also reacts with human PPIA). PPIB rabbit mAb was obtained from CST (#43603S). PPIB rabbit polyclonal antibody for immunofluorescence was obtained from Proteintech (#11607-1-AP). PDI mouse mAb was obtained from Novus Biologicals (#NB300-517). IMPDH2 rabbit polyclonal antibody was obtained from Proteintech (#12948-1-AP). Anti-Collagen I antibody (#ab138492) was obtained from Abcam. αSMA mouse monoclonal antibody was obtained from Sigma-Aldrich (#A5228). Smad3 (phospho S423 + S425) antibody was obtained from

Abcam (#ab52903). FAK antibody was obtained from CST (#3285), Phospho-FAK Tyr397 antibody was obtained from CST (#3283). N-Pro Collagen I antibody was obtained from R&D (#MAB6220). Antibodies against ER stress marker proteins were obtained from CST (#9956). GAPDH Rabbit mAb was obtained from CST (#2118L). Actin mouse mAb was obtained from Santa Cruz (#sc-47778). Goat anti-rabbit HRP secondary antibody was obtained from Rockland Immunochemicals (#611-1302) or Bio-Rad (#1706515).). Goat anti-mouse HRP was obtained from Rockland Immunochemicals (#610-1302) or Bio-Rad (#1706516). Goat anti-rabbit 680 secondary antibody was obtained from LI-COR Biosciences (#925-68071). Goat anti-mouse 680 secondary antibody was obtained from LI-COR (#925-68070). Goat anti-rabbit 800 secondary antibody was obtained from LI-COR (#925-3221). Goat anti-Mouse 800 secondary antibody was obtained from LI-COR Biosciences (#925-32210).

SDS-PAGE gel electrophoresis and Western blotting

SDS-PAGE gels were prepared in-house and were run in buffer diluted from 10× Tris-Glycine-SDS running buffer (Research Products International (RPI), #T3208010000.0). Gels were stained for total protein with GelCode Blue, obtained from Pierce (#24592). Gels were transferred to membranes with iBlot 2 nitrocellullose transfer stacks (ThermoFisher, #IB23001 and #IB23002). Ponceau S for total protein staining on membranes was obtained from Sigma Aldrich (#P3504-10G). BSA, heat shock fraction, was obtained from Sigma Aldrich (#A9647-1KG). Radiance Q chemiluminescence reagent was obtained from Azure Biosciences (#AC2101).

For detecting collagen by blot, LDS sample buffer (ThermoFisher, #NP0007) and NuPAGE 4–12% Bis-Tris gels (ThermoFisher, #NP0321BOX) were used. After SDS-PAGE, proteins were transferred to 0.45 μm PVDF membrane (ThermoFisher, #LC2005). BSA (Sigma, #A9430-100G) was used for blocking. ECL Prime Western Blotting Detection Reagent (Cytiva, #RPN2236) was used for detection.

Immunofluorescence imaging

Superfrost plus micro slides were obtained from VWR (#48311-703). Collagen coated coverslips (22 mm diameter, #1.5) were obtained from Neuvitro (#GG-22-1.5-Collagen). Prolong Diamond Anti-Fade Mountant was obtained from ThermoFisher (#P36961). Molecular Probes NucBlue Fixed Cell ReadyProbes Reagent was obtained from ThermoFisher (#R37606). AlexaFluor488 azide was obtained from Click Chemistry Tools (#1275-1).

Mass spectrometry sample preparation

S-Trap mini columns were obtained from Protifi (#C01-mini-80). Trypsin was obtained from Promega (#V5111). High pH fractionation was performed using either the Pierce high pH reversed-phase peptide fractionation kit (ThermoFisher #84868) or C18 spin columns (G-Biosciences, #786-930).

FPLC and protein purification

GSTrap FF columns were obtained from GE Healthcare (#17-5130-01).

Mouse study

C57BL/6N mice at 6 to 8 weeks of age were purchased from the National Cancer Institute Frederick Mouse Repository (Frederick, MD). Collagen-GFP reporter mice: Transgenic reporter mouse expressing GFP under the control of collagen type I promoter (col-GFP mice) were obtained from Jackson Laboratories, (Col1a1*2.3-GFP)1Rowe/J)). Kolliphor EL was obtained from Sigma-Aldrich (#C5135-500G). The hydroxyproline assay kit was purchased from Sigma-Aldrich (#MAK008). A bicinchoninic acid (BCA) protein assay kit was obtained from Pierce (#23209).

Liberase was obtained from Roche (#05401127001). DNase I was obtained from Sigma-Aldrich (#10104159001). CD16/CD32 antibody was obtained from BioLegend (#101320). The fluorophore-conjugated antibodies used included CD11b-BUV737 (Biolegend, #612800), Ly6G-FITC (Biolegend,

#551460), Ly6C-PerCP-Cy5.5 (Biolegend, #128012), CCR2-PE (R&D, #FAB5538P), CD11c-BV605 (Biolegend, #117334), MHCII-Pe-Cy7 (Biolegend, #107630), F4/80-PE (Biolegend, #123110), MerTK-APC (Biolegend, #151507), CD3-BUV395 (Biolegend, #563565), CD4-BV786 (Biolegend, #100453), CD8-FITC (Biolegend, #553031). eFluor 780 fixable viability dye was obtained from eBioscience (#1977884).

Other supplies

SfA was a generous gift from Novartis, Cambridge, MA. CsA was obtained from LC Laboratories (#C-6000). Vivaspin spin concentrators were obtained from GE Healthcare in various sizes and molecular weight cutoffs. Mini bio-spin columns were obtained from Bio-Rad (#7326207). EDTA-free protease inhibitor cocktail tablets were obtained from Sigma Aldrich (#11873580001). Brefeldin A was obtained from Adipogen (#AGCN20018M005). MLN4924 was obtained from SelleckChem (#S7109). MG132 was obtained from SelleckChem (#S2619). E64d was obtained from SelleckChem (#S7393). EZCell protein transport inhibitor cocktail was obtained from Biovision (#K932-500). Cycloheximide was obtained from VWR (#97064-724). The sircol collagen assay kit was obtained from Biocolor (#S1000).

Instrumentation

Proton nuclear magnetic resonance spectra (^{1}H NMR) were recorded at 400, 500, or 600 MHz at 24 °C. Chemical shifts are expressed in parts per million (ppm, δ scale) downfield from tetramethylsilane and are referenced to residual protium in the NMR solvent [$CHCl_3$, δ 7.26]. Data are represented as follows: chemical shift, multiplicity (s = singlet, d = doublet, t = triplet, q = quartet, quin = quintet, m = multiplet and/or multiple resonances, br = broad, app = apparent), integration, coupling constant in Hertz, and assignment. Proton-decoupled carbon nuclear magnetic resonance spectra (^{13}C NMR) were recorded at 100 or 125 MHz at 24 °C. Chemical shifts are expressed in parts per million (ppm, δ scale) downfield from tetramethylsilane and are referenced to the carbon resonances of the solvent ($CDCl_3$, δ 77.0). ^{13}C NMR data

are represented as follows: chemical shift, carbon type. Chemical shifts are expressed in parts per million (ppm, δ scale) downfield from tetramethylsilane. Infrared (IR) spectra were obtained using a Shimadzu 8400S FT-IR spectrometer referenced to a polystyrene standard. Data are represented as follows: frequency of absorption (cm^{-1}), intensity of absorption (s = strong, m = medium, w = weak, br = broad). Small molecule high-resolution mass spectrometry (HRMS) measurements were obtained using a Thermo Q-Exactive Plus. Low-resolution mass spectrometry (LRMS) measurements were obtained on a Waters ACQUITY UPLC equipped with SQ Detector 2 mass spectrometer. Ultrapure water was obtained using a GenPure UV/UF xCAD plus system (Thermo Scientific). All UV irradiation was performed using a Dymax ECE 5000 UV Light-Curing Flood Lamp system (#41060). The lamp was warmed up for at least 10 minutes prior to use. Irradiation was performed in a 4 ºC cold room for 60 s unless otherwise noted. For irradiation, samples were placed in 100 μL or 500 μL PPE PCR tubes or in a well plate with the lid removed. Bacterial growth by OD600, DNA concentrations by A260, and protein concentrations by A280 were measured using a NanodropOne (ThermoFisher). An E1-ClipTip electronic multichannel pipette (ThermoFisher) was used for set up of assays in 96 well plates. Transfer of proteins from SDS-PAGE gels to membranes was performed using a iBlot 2 blotting system (Invitrogen). In-gel fluorescence, GelCode Blue staining, IR blots, and Ponceau were imaged by an Azure Imager C600 (Azure Biosystems). Protein purification was performed using an ÄKTA pure 25 equipped with a F9-R fraction collector, a C9n conductivity monitor, and computer running UNICORN v6.3.2.89 (GE Healthcare). Peptides were dried using a Vacufuge plus (Eppendorf). Confocal imaging was performed using an Olympus Fluoview 3000 microscope. Proteomics mass spectrometry instrumentation is described in the "Proteomics Mass Spectrometry" section. Histology images were acquired with a Zeiss LSM780 confocal microscope (Zeiss). Flow cytometry was performed using a BD LSRFortessa X-20 cell analyzer. Precision-cut lung slices were prepared using a Compresstome® VF-310-0Z (Precisionary Instruments).

Software

Data was analyzed and visualized using Microsoft Excel (v16.55) and GraphPad Prism (v9.3.0). DNA and protein sequences were analyzed using Geneious (v11.0.3) or Genious Prime (v2021.2.2). Proteomics data was analyzed using Proteome Discoverer (v2.4.1.15) and quantified using Shiny App, an in-house quantification software (https://github.com/christinawoo/SHINYApp). Images were made using FIJI (NIH, v.2.0.0-rc-69/1.52p), Adobe Photoshop (v23.0.1), Adobe Illustrator (v26.0.1), and Biorender.

General synthetic procedures

All reactions were performed in single-neck, flame-dried, round-bottomed flasks fitted with rubber septa under a positive pressure of argon, unless otherwise noted. Air- and moisture-sensitive liquids were transferred *via* syringe or stainless steel cannula, or were handled in a nitrogen-filled glove box (working oxygen level <1 ppm). Organic solutions were concentrated by rotary evaporation at 24 °C. Normal and reverse phase flash-column chromatography was performed as described by Still and co-workers.(2) Normal phase purifications were performed using silica gel (60 Å, 40–63 µm particle size) purchased from Sorbent Technologies (Atlanta, GA). Reverse phase purifications were performed using C18-coupled silica gel (125 Å, 55–105 µm particle size) purchased from Waters Corporation (Milford, MA). Analytical thin-layered chromatography (TLC) was performed using glass plates pre-coated with silica gel (0.25 mm, 60 Å pore size) impregnated with a fluorescent indicator (254 nm). Preparative TLC (PTLC) was performed using glass plates precoated with silica gel (1.0 mm, 60 Å pore size) impregnated with a fluorescent indicator (254 nm). TLC plates were visualized by exposure to ultraviolet light (UV) and/or submersion in aqueous ceric ammonium molybdate solution (CAM), acidic *p*-anisaldehyde solution (PAA), or aqueous potassium permanganate solution ($KMnO_4$), followed by brief heating by heat gun (120 °C, 10–15 s).

Biological procedures

Mammalian cell culture

Jurkat, K562, A549, HeLa, and IMR-90 cells were cultured in standard media (DMEM or RPMI) supplemented with 10% fetal bovine serum and 1× Pen/Strep (100 U/mL penicillin, 100 μg/mL streptomycin). For IMR-90 cells, *L*-glutamine was added to DMEM without *L*-glutamine to a final concentration of 2 mM. Cells were incubated at 37 ˚C in humidified incubators with CO_2 maintained at 5%. Cells were routinely checked for mycoplasma contamination.

SDS-PAGE and Western blotting

SDS-PAGE was routinely performed on 6% stacking/12% separating gels cast in-house. Samples were prepared in Laemmli buffer (For 5× stock: 5% (v/v) β-mercaptoethanol, 0.02% (w/v) bromophenol blue, 30% (v/v) glycerol, 10% (w/v) sodium dodecyl sulfate (SDS)/250 mM Tris pH 6.8) and heated to 95 ˚C for 5–10 min to denature proteins. Samples were collected in the bottom of tubes by brief centrifugation. Gels were run in 1× Tris-Glycine-SDS running buffer for 1 h at 150 V. For blotting, proteins were transferred to nitrocellulose membranes using an iBlot 2 system with the P0 program (1 min at 20 V, 4 min at 23 V, 2 min at 25 V). Protein loading and transfer was visualized by brief Ponceau staining (0.2% (w/v) Ponceau S, 3% (v/v) acetic acid/H_2O) followed by washing with deionized water. Membranes were blocked in 5% BSA/TBST for 1 h, then incubated in primary antibody(s) diluted 1:1,000 in 5% BSA/TBST for 1 h at 24 ˚C or overnight at 4 ˚C. Overnight incubation was required for lower abundance samples (*i.e.*, enriched proteins). Primary antibody was collected for re-use and membranes were washed 3× with TBST. Membranes were then incubated in secondary antibody(s) diluted 1:10,000 in 5% BSA/TBST and incubated 45 min. The membranes were washed 3× with 15 mL TBST prior to imaging with IR or chemiluminescence.

For detecting collagen by blot, samples were mixed with LDS sample buffer, boiled at 95 ˚C for 10 min, and separated on a NuPAGE 4–12% Bis-Tris gel. After SDS-PAGE, proteins were transferred to a 0.45 μm PVDF membrane overnight at 25 V, 4 ˚C. The membrane was then blocked with 3% BSA/TBST,

followed by primary collagen antibody incubation overnight at 4 ˚C and subsequent secondary antibody incubation for 1 h at 24 ˚C. Chemiluminescence was imaged using film.

Cell viability assay (Jurkat, K562, and A549)

Cells were seeded in normal growth media in TC-treated 96 well plates at 10,000 cells/well (Jurkat) or 8,000 cells/well (K562 or A549). Wells bordering the plate edges were filled with 100 μL media. A549 cells were incubated overnight to allow for adhesion. Stock solutions (100×) were prepared in 50% DMSO/PBS by serial dilution. Cells were dosed with 1 μL stock solution per well with each condition in triplicate, then were incubated for 72 h at 37 ˚C, 5% CO_2. MTT solution (10 μL, 3 mg/mL/PBS) was added to each well, including several media-only wells, and allowed to incubate at 37 ˚C, 5% CO_2 for 3 h. Dissolving solution (100 μL, 10% (w/v) SDS, 0.01 M HCl/H_2O) was added to each well and allowed to incubate 18 h at 37 ˚C, 5% CO_2. Absorbance was then measured at 570 nm.

Overexpression and purification of recombinant proteins

pET41a-PPIB was constructed from pET41a and the PPIB ORF in pcDNA3.1-PPIB as previously described by Frick and coworkers(3) using forward primer GCGCGCACTAGTGATGAGAAGAAGAAGGGGC and reverse primer GCGCGCCTCGAGCTACTCCTTGGCGATGGC. pGEX 2TK-PPIA was used as received. Rosetta 2 (DE3) cells were transformed with pGEX-2TK-PPIA or pET41a-PPIB. From a glycerol stocks or agar plate colony, a 50 mL culture of LB media + 100 μg/mL ampicillin in a 250 mL baffled flask was inoculated and allowed to grow for 18 h at 30 ˚C, shaking at 200 rpm. For each overexpression culture, 750 mL of LB media was autoclaved in a 2 L baffled flask and allowed to cool, then warmed for 18 h at 30 ˚C, shaking at 200 rpm. The next day, ampicillin (100 μg/mL) was added to each overexpression culture, and each culture was inoculated with 7.5 mL of overnight culture. Cultures were allowed to grow until the OD_{600} was approximately 0.5–0.6. Protein expression was then induced with addition of IPTG to a final concentration

of 1 mM and the temperature was lowered to 24 ˚C. Expression was allowed to proceed for 21 h. Cells were then collected by centrifugation (15 min, 3,200 × g, 4 ˚C). Cell pellets were flash frozen with liquid nitrogen and stored at –80 ˚C.

Samples were taken throughout the purification process for monitoring by SDS-PAGE. All steps were performed on ice or in the 4 ˚C cold room if possible. To purify GST-PPIA or GST-PPIB, a cell pellet from a 750 mL overexpression was thawed and lysed by sonication (on ice, 30 s on, 10 s off, 25% amplitude, 5 min total) in 10 mL of 1 mM DTT, 1% Triton-X 100, 1× protease inhibitor cocktail/PBS. Lysates were clarified by centrifugation (10 min, 20,000 × g, 4˚ C) and filtration through a 0.45 μm syringe filter. Crude purification was performed used a GSTrap column with gradient elution (equilibration/wash buffer: 1 mM DTT/PBS, elution buffer: 1 mM DTT, 10 mM reduced glutathione/50 mM Tris pH 8.0). Protein-containing fractions were pooled and concentrated in a 3 kDa MWCO spin concentrator. Further purification and buffer exchange was performed by SEC on a Superdex 75 10/300 GL column with PBS as the running buffer. Protein was concentrated in a 3 kDa MWCO spin concentrator. The concentration was determined by A280 prior to aliquoting, flash freezing with liquid nitrogen, and storing at –80 ˚C.

In-gel fluorescence of photo-affinity labeled proteins

Solutions of GST-PPIA or GST-PPIB (10 μM, 20 μL) were prepared in 0.1% Triton-X 100/PBS and 10 μM pSfA or DMSO (final DMSO concentration 2.5%) was added. Samples were incubated at 4 ˚C for 30 min, then were irradiated for 30 s. AlexaFluor488 azide was appended to labeled proteins by a click reaction (final concentrations: 25 μM AlexaFluor488 azide, 100 μM THPTA, 1 mM CuSO4, 2 mM sodium ascorbate, 1% SDS). The reaction was incubated for 1 h at 24 ˚C. Cold acetone (100 μL) was added to each sample, and samples were incubated at –20 ˚C for 20 min prior to collecting protein by centrifugation (10 min, 21,000 × g, 4 ˚C). The supernatant was removed and the protein pellets were allowed to air dry before being dissolved in 20 μL of 1× SDS-PAGE loading buffer. Samples were heated to 95 ˚C for 5 min prior to running on a 6/12% acrylamide gel, which was fixed for 5 min with 40% MeOH, 10% acetic acid/H_2O

prior to imaging fluorescence. The gel was then stained with GelCode Blue and the total protein was imaged.

Binding affinity determination by TR-FRET

Cyclophilin binding affinity assays were performed by Eurofins Discovery. A 384 well plate-based TR-FRET assay was used to determine the ability of the supplied test inhibitors (SfA, pSfA1, pSfA2, and SfA-mc) to compete for cyclosporin A (CsA) binding to the respective cyclophilin enzymes (PPIA and PPIB). The assay determines the ability of the inhibitors to compete for CsA binding to the cyclophilin enzyme: the cyclophilin enzyme, tagged with a polyhistidine sequence, forms a complex with an anti-His_6 antibody labelled with a fluorescent donor, F(d), and the enzyme ligand CsA tagged with a fluorescent acceptor, F(a).

An 8 point dilution series of the supplied compounds was performed over a final concentration range of 0.01 nM–10 μM. Unlabeled CsA was used as a control over the concentration range 0.02 nM–20 μM. The inhibitor was added to the master mix in the assay plate containing the enzyme–antibody–ligand complex, with a final concentration of detergent of 0.005%. This was incubated for 30 min at room temperature and the plate read on a SpectraMax iD5 at 2 wavelengths (A and B) to detect emission from F(d) and F(a), respectively. The ratio B/A was calculated, blank subtracted, plotted against the inhibitor concentration in $\log_{10}$, and fitted using one site K_i nonlinear regression to determine the K_d.

Compound K_d values were determined from duplicate data points, as per the standard protocol at Eurofins. Each assay plate contained ‘no enzyme’ blanks and ‘no inhibitor’ controls, which were used to determine the Z’ value for each plate. CsA was used as a control. Plotted data from this assay is shown in Figure S2.

Photo-affinity labeling for target identification

Jurkat and K562 cells were grown to saturation. In each experiment, three treatments (minimalist tag, photo-SfA, and parent competition) were each tested in triplicate. For each set of samples, cell culture (50 mL per set of three samples, approximately 1.7×10^7 cells per sample) was collected by centrifugation (3 min, 500 × g, 24 ˚C) and the media was aspirated. Each cell pellet was washed twice with 25 mL PBS, centrifuging (3 min, 500 × g, 24 ˚C) and aspirating each wash. Each washed cell pellet was resuspended in 1.5 mL serum-free, antibiotic-free RPMI and aliquoted (0.5 mL/sample) into a 12 well plate. In each set of wells seeded from one cell suspension, one well was dosed with 5 μL 1 mM minimalist tag/DMSO, one well was dosed with 2.5 μL DMSO + 2.5 μL 2 mM photo-SfA/DMSO, and the final well was dosed with 2.5 μL 10 mM SfA/DMSO + 2.5 μL 2 mM photo-SfA/DMSO. Samples were incubated 30 min at 37 ˚C, 5% CO_2, then were irradiated for 60 s with the plate lid removed. Samples were transferred to 1.7 mL tubes, washing each well with 0.5 mL PBS. Cells were collected by centrifugation (3 min, 500 × g, 24 ˚C) and washed with 1 mL PBS.

Each sample was lysed by sonication (2 s on, 5 s off, 10% amplitude, 10 s total) in 250 μL 1% SDS, 1× protease inhibitor cocktail/PBS. Lysates were clarified by centrifugation (10 min, 21,330 × g, 4 ˚C), retaining only the soluble portion. Protein concentration was determined by BCA assay. Sample volumes and protein concentrations were normalized such that each 250 μL sample had an equal protein concentration (maximum 2.5 mg/mL). Biotin was attached to labeled proteins using a click reaction (final concentrations: 100 μM biotin-PEG_3-azide, 1 mM $CuSO_4$, 100 μM THPTA, 2 mM sodium ascorbate, incubated 1 h at 24 ˚C with inversion). Excess reagent was removed by precipitation, adding 1 mL MeOH to each sample, incubating at –80 ˚C for 1 h, and collecting precipitated protein by centrifugation (10 min, 21,330 × g, 4 ˚C). The resulting supernatants were carefully removed and the pellets were allowed to air dry for 5 min.

Each sample was resuspended in 1 mL of 1% SDS/PBS by sonication (2 s on, 5 s off, 15% amplitude, 20 s total). Washed streptavidin bead slurry (100 μL, 50% beads/PBS) was added to each sample

and incubated overnight at 24 ˚C with inversion. Samples were transferred to mini bio-spin columns on a vacuum manifold and beads were washed with 1 mL 1% SDS/PBS, 5× 1 mL 8 M urea/PBS, and 5× 1 mL PBS.

For MS analysis, each sample was resuspended in 175 μL PBS, then reduced (10 mM DTT, 24 ˚C, 30 min with inversion) and alkylated (20 mM iodoacetamide, 24 ˚C, 30 min with inversion). Beads were washed with 1 mL PBS and 1 mL 50 mM HEPES pH 8.5. In plugged columns, beads were resuspended in 250 μL 0.5 GdnHCl/50 mM HEPES pH 8.5 with 1.5 μg trypsin and incubated 16 h at 37 ˚C with inversion. The digested peptides were collected by centrifugation in 2 mL tubes, combining each digest with washes (200 μL water, 200 μL 50% acetonitrile/H_2O). Samples were dried by vacufuge. Each sample was resuspended in 100 μL 200 mM HEPES pH 8.5 and labeled with 5 μL TMT 10plex reagent. Labeling reactions were incubated at 24 ˚C and, after 1 h, were quenched with 6 μL 5% (v/v) hydroxylamine/H_2O for 15 min at 24 ˚C. Samples were combined and dried by vacufuge. Samples were desalted and separated by high pH reversed-phase fractionation according to kit instructions to 5 fractions (5%, 10%, 15%, 20%, and 50% acetonitrile, 0.1% triethylamine (TEA)/ H_2O) prior to LC-MS/MS analysis. In pSfA2 experiments, the 5% sample was excluded from analysis.

For gel analysis, washed beads were resuspended as a 50% slurry in 1.5× loading buffer, then heated at 95 ˚C and collected by brief centrifugation prior to SDS-PAGE. A small amount of each load sample (*i.e.*, proteins + beads prior to overnight incubation) was also collected for analysis.

Analysis of intra- and extracellular cyclophilin B and secretomics profiling in Jurkat cells

To analyze intracellular PPIB levels by blotting, Jurkat cells were seeded in a 12 well plate (1 × 10^6 cells/mL, 1 mL/well). Cells were treated with 200× solutions of the compounds of interest in DMSO and were incubated 4 h at 37 ˚C, 5% CO_2. Cells were collected by centrifugation. Cell pellets were washed with 1 mL PBS, then lysed in 100 μL 1× SDS-PAGE loading buffer by brief sonication (5 s, 10% amplitude).

To analyze extracellular PPIB levels, Jurkat cells were seeded at 9×10^5 cells/mL in 8 mL serum-free media. Cells were treated with 200× solutions of the compounds of interest in DMSO and incubated 4 h at 37 °C, 5% CO_2. Media was collected by centrifugation and remaining cells were removed by filtration through a 0.45 μm syringe filter. An equal volume of media from each sample was concentrated to < 320 μL with a 3 kDa MWCO spin filter. Volumes were normalized to 320 μL with PBS based on sample mass, and SDS (10% (w/v) stock in water) was added to a final concentration of 2%. For analysis by Western blotting, 80 μL of each sample was combined with 20 μL 5× loading buffer.

For secretomics, the remainder of each extracellular protein sample was reduced by the addition of DTT to a final concentration of 20 mM and heated at 95 °C for 10 min. Samples were allowed to cool to 24 °C prior to being alkylated by the addition of iodoacetamide to a final concentration of 40 mM. Alkylation was allowed to proceed for 30 min at 24 °C. Samples were prepared for S-Trap digestion by addition of 0.1 volume 12% phosphoric acid and 7 volumes S-Trap buffer (100 mM TEAB pH 7.1/90% MeOH/$H_2 0$). Each sample was loaded onto an S-Trap mini and washed 3 times with 400 μL S-Trap buffer. Trypsin (10.5 μg) in 125 μL 50 mM TEAB pH 8.5 was added to each sample, and digestion was allowed to proceed for 1 h at 47 °C. The digested peptides were eluted with 80 μL 50 mM TEAB pH 8.5, 80 μL 0.2% formic acid/H_2O, and 80 μL 50% acetonitrile/H_2O. Eluted peptides were dried by vacufuge and resuspended in 25 μL water. Samples were TMT labeled by addition of 10 μL TMT reagent to each sample and incubated 1 h at 24 °C. Labeling reactions were quenched by the addition of 6 μL 5% hydroxylamine/ H_2O and incubated 15 min at 24 °C. Samples were combined, dried by vacufuge, and separated by high pH reversed-phase fractionation (5%, 10%, 15%, 20%, and 50% acetonitrile, 0.1% TEA/H_2O), excluding the 5% fraction from analysis.

Proteomics mass spectrometry

Data was collected using an Orbitrap Lumos Tribrid and an Orbitrap Elite Hybrid Ion Trap-Orbitrap (both ThermoFisher). For both instruments, buffer A was 0.1% formic acid/H_2O and buffer B was 0.1% formic acid/acetonitrile.

The Orbitrap Lumos Tribrid was equipped with a Thermo UltiMate 3000 RSLCnano system. Peptides were separated using a C18 trap column (3 cm, 3 μm particle size C10 Dr. Maisch 150 μm ID) and an analytical column (Thermo Scientific Acclaim PepMap 100, 2 μm particle size, 250 mm length, 75 μm ID), maintained at 35 ˚C. In an example method, following sample injection, peptides were separated using a linear gradient from 7% to 37% B over 49 min with a flow rate of 200 nL/min. A gradient to 95% B was then applied over 15 min. Electrospray ionization was performed by applying a voltage of 2.2 kV. Survey scans were performed at a resolution of 120K over a range of 410–1,800 *m/z* with an AGC setting of 100,000 and a maximum injection time of 50 msec. HCD fragmentation with first mass 110 *m/z* was performed on the 10 most abundant precursors exhibiting a charge state of 2–5 with an isolation window of 0.8 kDa, a resolving power setting of 60K, a fragmentation energy of 38%, an AGC setting of 100,000, and a maximum injection time 150 msec in the Orbitrap. CID fragmentation was performed on the same ions and additional ions to use all available time over a range of 150–2000 *m/z* with an isolation window of 2 *m/z*, a fragmentation energy of 35%, an AGC target of 20,000, and a maximum injection time of 50 msec in the ion trap. Ions within a 10 ppm window of ions selected for MS2 fragmentation were excluded from further selection for fragmentation for 150 s.

The Orbitrap Elite was equipped with a Waters NanoAcquity HPLC pump and peptides were separated on a 100 μm inner diameter microcapillary trapping column packed first with approximately 5 cm of C18 Reprosil resin (5 μm, 100 Å, Dr. Maisch GmbH, Germany) followed by an analytical column of approximately 20 cm of Reprosil resin (1.8 μm, 200 Å, Dr. Maisch GmbH, Germany), maintained at 60 ˚C. Electrospray ionization was performed by applying a voltage of 1.8 kV. In an example method, following sample injection, a gradient of 4% to 24% B was applied over 90 min, then a gradient to 98% B

was applied over 14 min with a flow rate of 200 nL/min. Survey scans were performed over a range of 395–1,600 *m/z* at a resolution of 60K. CID fragmentation was performed over a range 110–2000 *m/z* on the 10 most abundant precursors exhibiting charge state 2–3 using a precursor isolation width window of 2 *m/z* and a normalized collision energy of 35 in the ion trap. The same ions were subjected to HCD fragmentation with first mass 100 *m/z* in the Orbitrap with a fragment ion isolation width of 1 *m/z*, a resolution of 60K, and a normalized collision energy of 37. Ions within a 5 ppm window of ions selected for MS2 fragmentation were excluded from further selection for fragmentation for 90 s.

Immunofluorescence imaging for PPIB

HeLa cells were grown to ~70% confluency on collagen-coated coverslips in a 6 well plate.

Cells were treated with SfA or CsA from 1000× stocks in DMSO and incubated for 4 h at 37 ˚C, 5% CO_2. Media was removed and each well was washed with 1 mL PBS. Samples were fixed in –20 ˚C EtOH (190 proof) for 10 min at 24 ˚C with shaking, then were washed 3× with 1 mL PBS. Samples were permeabilized in 0.2% Triton-X 100/PBS for 5 min at 24 ˚C with shaking, then were washed with PBS (3 ×, 1 mL, 3 min). Samples were blocked in 5% BSA/PBS for 1 h at 24 ˚C with shaking. Samples were incubated in anti-PPIB (1:100) and anti-PDI (1:250) primary antibodies in 1% BSA/TBST overnight at 4 ˚C with shaking. Primary antibody solution was removed and each sample was washed with PBS (3 ×, 1 mL, 3 min). Samples were incubated in goat anti-rabbit 488 (1:600) and goat anti-mouse 647 (1:600) secondary antibodies in 1% BSA/TBST for 1 h at 24 ˚C with shaking, protected from light. Secondary antibody solution was removed and each sample was washed 3× with 1 mL PBS. Nuclei were stained with NucBlue Fixed Cell ReadyProbes Reagent and coverslips were mounted on slides with Prolong Diamond Anti-Fade Mountant. After curing, slides were sealed with nail polish and imaged.

After imaging, slides were stored at –20 ˚C.

Immunofluorescence imaging for pSfA2

1.0 mL of HeLa cells at 0.5 million cells per mL were seeded onto poly lysine-coated coverslips (Electron Microscopy Sciences, Cat#72292) in a 6 well plate. Cells were treated with pSfA2 from 100× stocks in DMSO and incubated for 30 min at 37 °C, 5% CO2 in DMEM. Cells were UV irradiated for 60 seconds at 4 °C. Media was removed and each well was washed with 1 mL PBS. Samples were fixed with PBS containing 4% formaldehyde for 10 min at 24 °C. Samples were permeabilized in 0.1% Triton-X 100/PBS for 15 min at 24 °C, with shaking, then were washed with PBS (3 × 1 mL, 3 min). The samples were then tagged with AZDye 488 Azide probe (Alexa488 azide, Click Chemistry Tools, Cat#1275) by copper-click chemistry for 2 h at 37 °C. The clicked samples were washed with PBS (3 × 1 mL, 3 min) and blocked in 1% BSA/PBST and 22.5 mg/mL of glycine for 30 min at 24 °C with shaking. Samples were incubated in anti-calnexin (1:100) primary antibodies in 1% BSA/PBST for 1 h at 24 °C, with shaking. Primary antibody solution was removed, and each sample was washed with PBS (3 ×, 1 mL, 3 min). Samples were incubated in goat anti-rabbit 488 (1:500) and goat anti-mouse 568 (1:500) (Invitrogen, Cat# A-11031), secondary antibodies in 1% BSA/PBST for 1 h at 24 °C with shaking, protected from light. Secondary antibody solution was removed, and each sample was washed 3 × 1 mL PBS. Nuclei were stained and cells mounted on slides with ProLong™ Glass Antifade Mountant with NucBlue™ Stain (Invitrogen, P26985). After curing, slides were sealed with clear nail polish and imaged. After imaging, slides were stored at –20 °C. Pearson's Correlation was evaluated using Olympus cellSens software using minimum intensity thresholds for each channel on multiple images within each condition.

Fibroblast treatment

IMR-90 cells were seeded in a 6 well plate at 150,000 cells/well. Cells were incubated overnight at 37 ˚C, 5% CO_2 to allow for adhesion. The next day, the cells were washed with PBS and starved by incubating with serum-free media for 6 h at 37 ˚C. A stock solution of SfA (10 mM) was diluted to a final

working concentration of 1 μM using serum-free DMEM supplemented with 50 μg/ml *L*-ascorbic acid and was added to the appropriate wells. After 2 h of incubation at 37˚C, 10 ng/mL TGF-β1 was added to the appropriate wells and allowed to incubate at 37 ˚C, 5% CO_2 for a total of 96 h. For the experiments with Brefeldin A, a dose of 10.6 μM of Brefeldin A was added together with 1 μM SfA and 10 ng/mL TGFβ for 12 h. Cell supernatants and cell lysates were harvested and stored at –80 ˚C for downstream assays.

Fibroblast secretomics

Conditioned media from IMR-90 fibroblasts was collected following SfA treatment and TGF-β1 activation, and cells were removed using a cell strainer.

For each sample, 1.8 mL media was concentrated to ~250 μL in a 3 kDa MWCO spin concentrator. Volumes were adjusted based on sample weight to 250 μL per sample. SDS was added a final concentration of 1%, and 50 μL was taken blotting.

The remaining sample was prepared for MS analysis as described above for Jurkat cells.

Sircol collagen assay

For each sample, 1 mL of culture medium collected after indicated treatments was incubated with 200 μL of Isolation & Concentration reagent at 4 ˚C for 16 h. After centrifugation at (10 min, 12,000 rpm), the supernatant was removed, 1 mL of Sircol dye reagent was added, and samples were incubated for 30 min at 24 ˚C with shaking. After centrifugation (10 min, 12,000 rpm), the pellets of collagen–dye complex were washed twice with Acid-Salt wash reagent and centrifuged again (10 min, 12,000 rpm). Then, 250 μL Alkali reagent was added and the samples were incubated for 5 min. The contents of tubes were transferred to a 96 well plate and absorbance was measured at 555 nm on a plate reader. Collagen content was calculated based on a collagen type I standard curve.

Picrosirius red staining

After indicated treatments, cells were fixed by incubating with 4% paraformaldehyde at room temperature for 10 min. After one wash with PBS, cells were exposed to phosphomolybdic acid for 2 min, rinsed with distilled water, and stained in picrosirius red for 60 min at 24 ˚C. The cells were then washed with 0.1 N hydrochloric acid for 2 minutes, followed by a wash with 70% ethanol. A cover glass was applied, and the cells were imaged under brightfield and a polarized light field.

Fibroblast viability assay (crystal violet)

IMR-90 cells were seeded in 96 well plates at 5,000 cells/well. Cells were incubated for 18–24 h at 37˚C to allow for adhesion. Cells were then treated with 1 µM, 3 µM, or 5 µM of SfA or CsA in serum-free medium for 96 h. Then, the cells were washed twice with PBS and 50 µL of 0.5% crystal violet staining solution was added to each well and incubated for 20 min at 24 ˚C. Next, each well was washed 4× with PBS and allowed to air dry for 4 h at 24 ˚C. Methanol (200 µL) was added to each well and the plate was incubated for 20 min at 24 ˚C on a rocker. Absorbance was then measured at 570 nm.

Scratch assay

Human primary fibroblasts from healthy donors treated with or without TGF-beta were cultured until reaching confluency. A vertical scratch was then performed with a 200µL pipette tip. Immediately after, the medium was changed and the cells were treated with DMSO (control) or with SfA 1 µM in triplicate. Wells were monitored until all wounds were closed at 24/36h and wound closure was quantified using ImageJ.

Mouse study methods

C57BL/6N mice purchased at 6 to 8 weeks of age were used throughout this study. All experiments were performed in accordance with National Institutes of Health guidelines and protocols approved by the

Massachusetts General Hospital Subcommittee on Research Animal Care, and all mice were maintained in a specific pathogen–free environment certified by the Association for Assessment and Accreditation of Laboratory Animal Care (AAALAC). Skin and lung fibrosis was induced by daily subcutaneous injection of bleomycin (2 × 100 μL, 0.5 mg/mL) for 28 d. Sterile saline was used as control. For the in vivo drug studies, SfA was dissolved in 5% ethanol, 5% Kolliphor EL/sterile saline and mice were administered 10 mg/kg body weight once daily by intraperitoneal injection in a total volume of 100 μL. Drug dosing began 14 d after the bleomycin or saline treatment started and was maintained daily throughout the end of the study. At the end of experiments, mice were sacrificed, and full-thickness 6 mm punch biopsies and lungs were obtained for histologic and immunohistochemical studies as well as hydroxyproline analysis.

Picrosirius red staining for histology

Harvested 6 mm punch skin and left lobe lung biopsies were fixed in 10% buffered formalin for 72 h and were embedded in paraffin blocks. After deparaffinization, 5 μm sections of the lung punch tissue and cross sections of the skin were stained with picrosirius red according to the standard protocols previously described.

Dermal thickness

Dermal thickness was determined using whole scans of picrosirius red–stained skin cross sections, measuring the distance between the epidermal–dermal junction and the dermal–fat junction at ten randomly selected sites per high-power field. All investigations were performed in a blinded fashion.

Hydroxyproline measurement of skin and lung tissue

Right lung lobes and 6 mm punch skin biopsies were harvested and homogenized in 100 μL or 1 mL of distilled water respectively. Then, 100 μL of each sample was hydrolyzed in 100 μL of 12 M HCl at 120°C for 3 h. After hydrolysis, the volumes were measured to account for evaporation. Then, 5 μL of

each hydrolyzed lung and 100 μL of each hydrolyzed skin sample was lyophilized using a vacufuge at 45 °C for 90 min. Collagen content was then measured using the hydroxyproline assay kit according the prescribed protocol.

Bronchoalveolar lavage

To obtain bronchoalveolar lavage (BAL) samples, lungs were lavaged six times with 0.5 mL PBS. BAL samples were clarified by centrifugation (1,500 rpm, 5 min, 4 ˚C) and the supernatants and pelleted cells were transferred to siliconized low-binding Eppendorf tubes for subsequent analysis. The total number of cells in BAL samples was determined using an automated cell counter (Corning).

Vascular leak assay

Total protein concentration in BAL samples was determined using a commercially available bicinchoninic acid (BCA) protein assay kit per manufacturer's protocol.

Flow cytometry

Single-cell suspensions were isolated from 6 mm skin biopsies by incubating in Liberase (final concentration, 0.1 mg/mL) and DNase I (final concentration, 60 mg/mL) on a shaker for 45 min at 37 °C, followed by filtration through a 40 μm cell strainer. Cells from BAL samples were used directly without DNAse digestion. The number of cells from each sample was counted with a hemocytometer. Cells were incubated with CD16/CD32 antibody for 10 min at 4 °C followed by staining with fluorophore-conjugated antibodies. eFluor 780 fixable viability dye staining was performed to exclude dead cells. Flow cytometry was performed using a BDLSRFortessa X-20 cell analyzer, and FlowJo software was used for analysis.

Multiplexed immunofluorescence for the analysis of profibrotic macrophages

Lung tissue sections (7 μm thick) were stained with Opal 7-Color Automation IHC Kits (Akoya Bioscience) in the BOND-RX Multiplex IHC Stainer (Leica). Each section was put through 5 sequential rounds of staining, which included blocking in 5% BSA followed by incubation with primary antibodies (SPP1, Cell Signaling, 88742; FABP5, Cell Signaling, 39926; CD9, Novus, NBP2-67310; CD45, Cell Signaling, 70257; F4/80, Cell Signaling, 700765), corresponding secondary HRP-conjugated antibodies and Opal fluorophores as described (Strack et al., Clin Transl Med, 2020). Nuclei were counterstained with 4′,6-diamidino-2-phenylindole (DAPI) contained in the Opal 7-Color Automation IHC Kits, and slides were mounted with Fluoromount-G (SouthernBiotech). Imaging was performed with the VectraPolaris imaging system (Akoya Bioscience), and images were analyzed by using the phenotyping application of the inForm software V2.4.10 (Akoya Bioscience).

Primary cell culture methods

Primary fibroblasts

Subjects with IPF were identified from those receiving care at the Massachusetts General Hospital. IPF subjects had to satisfy IPF diagnostic criteria based on the 2011 recent joint consensus statement of the American Thoracic Society (ATS), European Respiratory Society (ERS), Japanese Respiratory Society, and Latin American Thoracic Association as determined by two investigators. Fresh lung specimens and lung fibroblasts were obtained from 3 IPF patients that underwent lung transplantation. Healthy lung fibroblast cultures were derived from adjacent normal tumor-free lung tissues from patients with non-small cell lung cancer tumors. These studies were approved by the MGH Institutional Review Board (Partners IRB 2013P002332), and informed consent was obtained from all participants.

Precision-cut lung slice experiments

Upon receiving lung tissue from patients with IPF, the tissue was inflated by injecting warm (37°C) 2% low-melting agarose/HBSS solution via a catheter cannulated in the bronchus. After complete solidification of agarose in the inflated lobes on ice, tissue blocks of approximately 1 cm in each dimension were prepared and mounted for slicing into 300 μm slices using a Compresstome® VF-310-0Z.

Lung slices were cut perpendicularly to the visible airway with a vibratome at room temperature. After overnight incubation at 37 °C in 5% CO_2 and DMEM/ F-12 supplemented with antibiotics, the collected lung slices were ready for downstream experiments. Cultures were treated for 3 days prior to collection for analysis.

Lung fibroblast treatment

Lung fibroblasts isolated from individuals with IPF and healthy controls were maintained in DMEM + 10% FBS in a humidified incubator at 5% CO_2. For assays, cells at passage 2–6 were seeded in 6 well plates at 150,000 cells/well. After cell adhesion, a stock solution of SfA (10 mM) was diluted to final concentration of 1 μM in serum-free DMEM supplemented with 50 μg/ml *L*-ascorbic acid and was added to the appropriate wells for 96 h at 37 ˚C. Cell supernatants and cell lysates were harvested and stored at –80 ˚C for downstream assays.

Quantification and statistical analysis

Mass spectrometry

Mass spectrometry data was analyzed in Proteome Discoverer 2.4.1.15. Spectra were searched against the Uniprot 2016 Swissprot database and a database of common contaminant proteins with a fully specific tryptic digest, up to two missed cleavages, minimum peptide length six, and maximum peptide length 120. A precursor tolerance of 10 ppm was used; fragment tolerances were 0.6 Da for CID spectra and 0.02 Da for HCD spectra. Modifications included oxidation (+15.995 Da on methionine, variable),

TMT6plex (+229.163 Da, static on peptide N termini and lysine) or TMTpro (+304.207, static on peptide N termini and lysine), and carbamidomethylation (+57.021 Da on cysteine, static). Matches were validated using the Percolator node. Reporter ions were quantified in HCD spectra. For PAL data, quantification and analysis was performed in Proteome Discoverer. Unique and razor peptides were used to determine protein abundance, abundance was normalized based to the total peptide signal in each channel, and p-values were determined by t-test. For secretomics data, peptide-level data from Proteome Discoverer was analyzed with an in-house algorithm utilizing a random tree model to impute missing quantification values. For both PAL and secretomics data, proteins were filtered to include only non-contaminant master proteins identified with high confidence and at least 2 unique peptides.

Cell viability

Cell viability was determined by taking the absorbance in vehicle-treated wells to be 100% and the absorbance in media-only wells to be 0%. Data was fit to a Sigmoidal, 4PL, X is $\log_{10}$(concentration) curve using the least squares method, removing outliers with Q = 1 and constraining the maximum equal to 100% and the minimum greater than 0%.

Synthetic procedures

*Synthesis of TBS-protected sanglifehrin A (**S1**)*

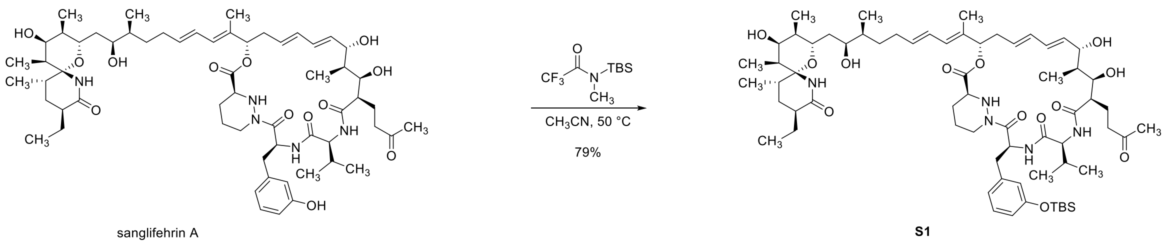

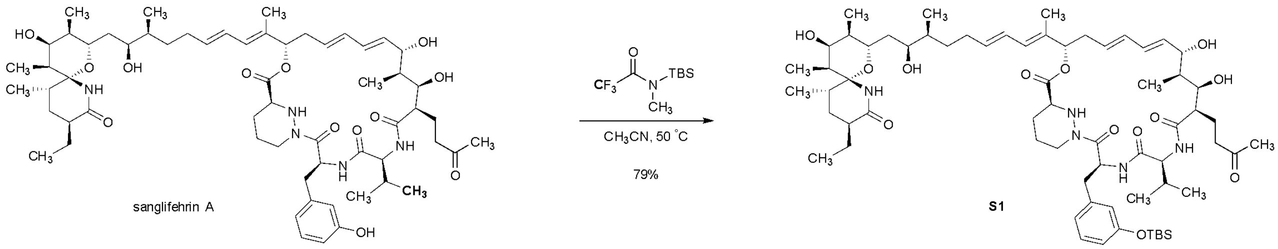

N-*tert*-butyldimethylsilyl-*N*-methyltrifluoroacetamide (2.2 mg, 9.2 μmol, 2.0 equiv) was added to a solution of sanglifehrin A (5.0 mg, 4.6 μmol, 1.0 equiv) in anhydrous acetonitrile (50 μL) at 24 ˚C. The mixture was warmed in an oil bath at 50 ˚C for 2 h. After cooling to 24 ˚C, ethyl acetate (5.0 mL) and water (5.0 mL) were sequentially added to the reaction mixture. After stirring for 5 min, the mixture was poured into a separatory funnel, and the layers were separated. The aqueous layer was extracted with ethyl acetate (5 mL × 2). The organic layers were combined and dried over anhydrous sodium sulfate. The mixture was filtered through a plug of cotton, and the resulting filtrate was concentrated under vacuum. The crude product was purified by column chromatography (SiO_2, 83% ethyl acetate–hexanes) to afford the TBS-protected sanglifehrin A **S1** (4.4 mg, 79%) as colorless oil.

R_f=0.1 (100% ethyl acetate, anisaldehyde). ^{1}H (500 Hz, $CDCl_3$): 8.46 (s, 1H), 7.19 (dd, *J*=8.0, 8.0 Hz, 1H), 7.19 (dd, *J*=8.0, 8.0 Hz, 1H), 7.00 (brs, 1H), 6.86 (dd, *J*=8.0 Hz, 1H), 6.74 (d, *J*=8.0 Hz, 1H), 6.73 (dd, *J*=8.0, 2.2 Hz, 1H), 6.68 (brs, 1H), 6.27 (dd, *J*=12.8, 4.4 Hz, 1H), 6.21 (dd, *J*=15.5, 11.2 Hz, 1H), 6.12 (dd, *J*=15.1, 10.6 Hz, 1H), 5.99 (d, *J*=10.6 Hz, 1H), 5.80 (ddd, *J*=8.5, 8.5, 6.4 Hz, 1H), 5.71 (dt, *J*=15.1, 7.0 Hz, 1H), 5.62 (dd, *J*=15.1, 11.2 Hz, 1H), 5.59 (dt, *J*=15.1, 7.1 Hz, 1H), 5.35 (dd, *J*=15.5, 2.5 Hz, 1H), 5.31

(m, 1H), 5.13 (m, 1H), 4.42 (brs, 1H), 4.08 (br, 1H), 3.93 (m, 1H), 3.83 (m, 2H), 3.76 (m, 2H), 3.59 (m, 1H), 2.89 (m, 2H), 2.53 (ddd, *J*=18.0, 7.5, 7.5 Hz, 1H), 2.45 (dd, *J*=10.5, 5.4 Hz, 1H), 2.43 (m, 1H), 2.35 (m, 1H), 2.21 (m, 2H), 2.13 (s, 3H), 2.09 (s, 1H), 2.03 (m, 1H), 2.00 (m, 3H), 1.94 (m, 2H), 1.85 (m, 1H), 1.79 (m, 1H), 1.73 (s, 3H), 1.70 (m, 4H), 1.59 (dd, *J*=14.8, 5.7 Hz, 1H), 1.59 (m, 1H), 1.50 (m, 2H), 1.45 (m, 4H), 1.43 (m, 1H), 1.30-1.20 (m, 4H), 1.21 (m, 1H), 0.82 (m, 1H), 1.01 (d, *J*=7.1 Hz, 3H), 0.98 (d, *J*=7.2 Hz, 3H), 0.96 (d, *J*=7.3 Hz, 3H), 0.96 (s, 9H), 0.96 (d, *J*=6.5 Hz, 3H), 0.96 (d, *J*=6.5 Hz, 3H), 0.93 (d, *J*=6.7 Hz, 3H), 0.91 (d, *J*=6.9 Hz, 3H), 0.18 (s, 6H). ^{13}C (500 Hz, $CDCl_3$): 207.7, 177.2, 175.6, 171.7, 171.6, 170.9, 155.9, 137.3, 136.6, 132.4, 132.2, 132.1, 130.9, 130.1, 129.7, 127.3, 125.5, 123.1, 122.0, 118.6, 88.5, 78.5, 78.1, 74.2, 73.8, 71.2, 68.6, 60.6, 58.5, 48.7, 47.5, 41.6, 41.2, 40.7, 39.4, 38.8, 38.3, 37.7, 37.2, 34.8, 32.2, 30.8, 30.1, 29.8, 29.7, 29.5, 28.0, 27.7, 26.8, 25.7, 22.3, 19.3, 18.9, 18.2, 14.9, 14.4, 14.1, 13.8, 13.0, 12.7, 12.1, -4.4, -4.4. IR: 3485.6 (br), 3307.5 (br), 3009.0 (w), 2958.3 (s), 2924.6 (s), 2854.3 (s), 1720.4 (w), 1641.7 (s), 1584.0 (s), 1553.1 (m), 1485.0 (m), 1461.8 (m), 1390.8 (m), 1250.7 (s), 1161.5 (s), 1085.4 (s), 1030.7 (m), 1004.6 (s). HRMS-ESI (m/z): $[M+NH_4]^+$ calculated for $C_{66}H_{109}N_6O_{13}Si$, 1221.7822; found 1221.7822.

*Synthesis of pSfA1 (**1**)*

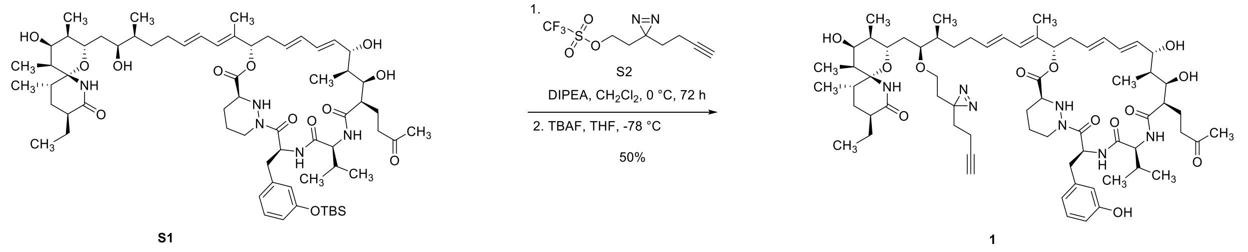

N,N-diisopropylethylamine (3.3 mg, 26 μmol, 6.0 equiv) and minimalist triflate **S2** (5.7 mg, 21 μmol, 5.0 equiv, prepared as previously reported(4)) were added to a solution of TBS-protected sanglifehrin A **S1** (5.0 mg, 4.3 μmol, 1.0 equiv) in anhydrous dichloromethane (0.5 mL) at 0 ˚C. The mixture was kept at 4 ˚C for 72 h. The reaction was quenched by addition of saturated aqueous sodium bicarbonate (1.0 mL), and the resulting mixture was stirred for 5 min at 24 ˚C. The mixture was poured into a separatory funnel, and the layers were separated. The aqueous layer was extracted with ethyl acetate (5 mL × 2). The organic layers were combined and dried over anhydrous sodium sulfate. The dried solution was filtered through a plug of cotton, and the resulting filtrate was concentrated under vacuum. The crude product was subjected to the next step without further purification.

The crude product was redissolved in anhydrous tetrahydrofuran (0.5 mL). The mixture was cooled to −78 ˚C and tetrabutylammonium fluoride (0.005 mL, 5.0 μmol, 1.2 equiv) was added. The reaction was stirred for an additional 10 min and was quenched by addition of saturated ammonium chloride (0.5 mL). After the mixture was warmed to 24 ˚C, it was poured into a separatory funnel. The layers were separated, and the aqueous layer was extracted with ethyl acetate (2 mL × 2). The organic layers were combined and dried over anhydrous sodium sulfate. The dried solution was filtered through a plug of cotton, and the resulting filtrate was concentrated. The crude product was purified by column chromatography (SiO_2, 100% ethyl acetate) to afford minimalist-tagged pSfA1 **1** (2.6 mg, 50%).

R_f=0.16 (100% ethyl acetate, anisaldehyde). ^{1}H (500 Hz, $CDCl_3$): 8.25 (brs, 1H), 7.38 (m, 1H), 7.15 (td, *J*=7.6, 7.6 Hz, 1H), 7.09 (s, 1H), 6.76 (dd, *J*=7.6, 2.4 Hz, 1H), 6.65 (d, *J*=7.6 Hz, 1H), 6.42 (d, *J*=3.5

Hz, 1H), 6.25 (dd, *J*=15.5, 11.4 Hz, 1H), 6.06 (d, *J*=11.4 Hz, 1H), 5.99 (dd, *J*=14.2, 10.2 Hz, 1H), 5.85 (m, 1H), 5.76 (dt, *J*=15.5, 6.95 Hz, 1H), 5.69 (brs, 1H), 5.56 (m, 1H), 5.46 (dd, *J*=15.5, 10.8 Hz, 1H), 5.40 (dd, *J*=14.2, 3.9 Hz, 1H), 5.32 (d, *J*=0.6 Hz, 1H), 5.26 (dd, *J*=15.5, 5.8 Hz, 1H), 5.13 (brs, 1H), 5.12 (brs, 1H), 4.51 (d, *J*=10.6 Hz, 1H), 4.12 (m, 1H), 4.07 (m, 1H), 3.85 (m, 1H), 3.82 (m, 1H), 3.80 (m, 1H), 3.73 (m, 1H), 3.69 (m, 1H), 3.67 (m, 1H), 3.17 (m, 1H), 3.08 (m, 1H), 2.93 (dd, *J*=14.5, 8.1 Hz, 1H), 2.77 (dd, *J*=14.5, 5.0 Hz, 1H), 2.68 (m, 1H), 2.58 (ddd, *J*=17.4, 9.0, 8.3 Hz, 1H), 2.47 (m, 1H), 2.44 (m, 1H), 2.41 (m, 1H), 2.23 (m, 1H), 2.17 (s, 3H), 2.15 (m, 1H), 2.02 (m, 1H), 1.94 (dd, *J*=7.1, 2.6 Hz, 1H), 1.89 (m, 1H), 1.82 (m, 2H), 1.77 (s, 3H), 1.75 (m, 1H), 1.70 (dd, *J*=7.6, 7.6 Hz, 1H), 1.50 (m, 2H), 1.30-1.24 (m, 12H), 1.03 (d, *J*=6.6 Hz, 3H), 1.01 (d, *J*=7.2 Hz, 3H), 0.99 (d, *J*=6.0 Hz, 3H), 0.96 (d, *J*=7.3 Hz, 3H), 0.94 (d, *J*=6.5 Hz, 3H), 0.94 (d, *J*=6.5 Hz, 3H), 0.85 (d, *J*=7.1 Hz, 3H). ^{13}C (500 Hz, $CDCl_3$): 207.4, 176.7, 172.1, 171.9, 171.2, 170.7, 157.1, 137.3, 136.8, 132.3, 132.0, 132.0, 130.8, 129.9, 129.8, 127.3, 125.5, 120.8, 117.0, 114.7, 89.5, 82.6, 78.9, 78.4, 75.6, 73.9, 71.5, 69.3, 69.3, 60.6, 60.4, 60.1, 56.0, 49.4, 47.2, 41.7, 40.7, 40.7, 39.5, 38.6, 38.2, 37.7, 37.5, 36.8, 34.7, 32.5, 32.5, 32.0, 30.8, 30.1, 29.9, 29.7, 28.4, 27.6, 26.6, 25.0, 22.7, 21.1, 19.3, 19.1, 15.4, 14.6, 14.2, 14.1, 14.0, 13.3, 12.9, 12.2. IR: 3326.7 (br), 2961.0 (s), 2926.2 (s), 2854.9 (m), 1719.9 (m), 1642.2 (m), 1599.9 (m), 1553.7 (m), 1378.4 (br), 1262.0 (s), 1163.0 (s), 1066.3 (s), 1050.0 (s), 1050.0 (s), 1032.6 (s), 1018.6 (s). HRMS-ESI (m/z): $[M+NH_4]^+$ calculated for $C_{67}H_{103}N_8O_{13}$, 1227.7645; found 1227.7645.

*Synthesis of pSfA2 (**2**)*

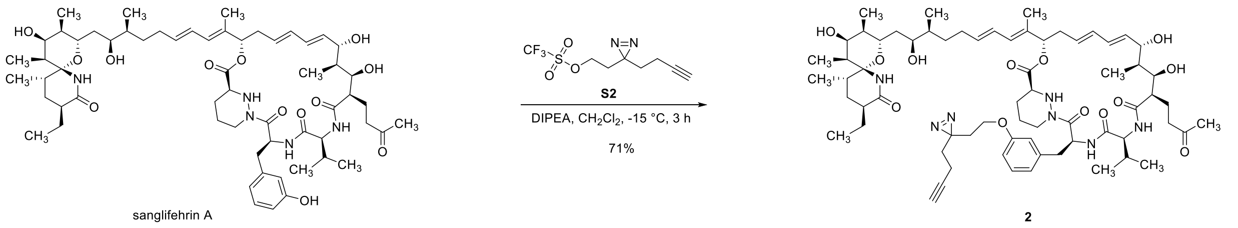

N,N-diisopropylethylamine (1.1 mg, 8.7 μmol, 2.0 equiv) and minimalist triflate **S2** (1.1 mg, 4.2 μmol, 1.0 equiv, prepared as previously reported (*6*)) were added to a solution of sanglifehrin A (5.0 mg, 4.6 μmol, 1.0 equiv) at −15 ˚C. The mixture was kept at −15 ˚C for 24 h. The reaction was quenched by addition of saturated aqueous sodium bicarbonate (0.5 mL), and the resulting mixture was stirred for 5 min at 24 ˚C. The mixture was poured into a separatory funnel, and the layers were separated. The aqueous layer was extracted with ethyl acetate (1 mL × 2). The organic layers were combined and dried over anhydrous sodium sulfate. The dried solution was filtered through a plug of cotton, and the resulting filtrate was concentrated under vacuum. The crude product was purified by column chromatography (SiO_2, 100% ethyl acetate) to afford pSfA2 **2** (3.9 mg, 71%) as colorless oil.

R_f=0.25 (100% ethyl acetate, anisaldehyde). ^{1}H (500 Hz, $CDCl_3$): 8.10 (brs, 1H), 7.38 (brs, 1H), 7.14 (dd, *J*=7.9, 7.9 Hz, 1H), 7.08 (brs, 1H), 6.80 (brs, 1H), 6.76 (d, *J*=7.9 Hz, 1H), 6.64 (d, *J*=7.9 Hz, 1H), 6.39 (m, 1H), 6.25 (dd, *J*=12.8, 4.4 Hz, 1H), 6.22 (dd, *J*=15.1, 10.6 Hz, 1H), 6.04 (d, *J*=10.6 Hz, 1H), 5.96 (m, 1H), 5.82 (m, 1H), 5.76 (m, 1H), 5.63 (m, 1H), 5.62 (m, 1H), 5.52 (m, 1H), 5.43 (m, 1H), 5.39 (m, 1H), 5.21 (m, 1H), 4.50 (m, 1H), 4.07 (m, 2H), 3.83 (m, 4H), 3.63 (m, 1H), 2.93 (m, 1H), 2.56 (m, 2H), 2.44 (m, 4H), 2.19 (m, 1H), 2.17 (s, 3H), 2.10-1.10 (m, 33H), 1.77 (s, 3H), 1.07-0.89 (m, 21H), 0.83 (d, *J*=7.7 Hz, 3H). ^{13}C (500 Hz, $CDCl_3$): 207.4, 176.8, 172.0, 171.9, 170.7, 168.4, 157.1, 137.4, 136.8, 132.3, 132.0, 131.9, 130.9, 129.9, 129.8, 127.3, 125.5, 120.8, 117.0, 114.7, 89.5, 82.6, 78.9, 78.4, 75.6, 73.9, 71.5, 69.3, 69.3, 60.6, 60.0, 59.6, 57.4, 49.4, 47.2, 41.7, 40.7, 40.7, 39.5, 38.2, 38.2, 37.7, 37.5, 35.5, 34.7, 32.6, 32.5, 32.2, 32.0, 30.8, 30.1, 29.9, 29.7, 28.4, 27.6, 26.6, 25.0, 22.7, 19.3, 19.1, 15.4, 14.6, 14.1, 14.0, 13.3, 13.2,

12.9, 12.2. IR: 3297.3 (br), 2961.5 (m), 2923.1 (m), 2852.2 (m), 1711.0 (m), 1640.2 (m), 1512.4 (m), 1454.3 (m), 1393.8 (m), 1258.3 (s), 1159.3 (s), 1085.8 (s), 1030.6 (s). HRMS-ESI (m/z): $[M+NH_4]^+$ calculated for $C_{67}H_{103}N_8O_{13}$, 1227.7645; found 1227.7645.

*Synthesis of (3S,6S,9R,10R,11S,12S,13E,15E,18S,21S)-10,12-bis((tert-butyldimethylsilyl)oxy)-3-(3-hydroxybenzyl)-18-((2E,4E)-9-hydroxynona-2,4-dien-2-yl)-6-isopropyl-11-methyl-9-(2-(2-methyl-1,3-dioxolan-2-yl)ethyl)-19-oxa-1,4,7,25-tetraazabicyclo[19.3.1]pentacosa-13,15-diene-2,5,8,20-tetraone (**S5**).*

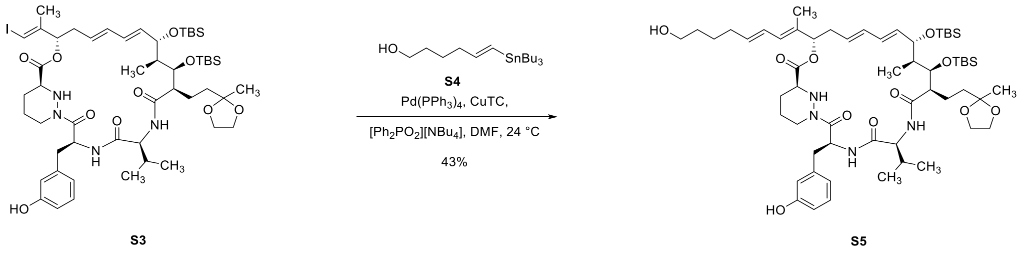

The iodide macrocycle **S3** (16.6 mg, 15.0 μmol, 1.00 equiv, prepared as previously described(5) and the stannane **S4** (5.84 mg, 15.0 μmol, 1.00 equiv) were concentrated together from dichloromethane in a pear-shaped flask (10. mL) and dried under vacuum for 8 h at 0 °C. The flask was replenished with argon and dimethylformamide (0.3 mL) was injected. The mixture was cooled to 0 °C and a solution of tetrabutylammonium diphenylphosphinate (13.8 mg, 30.0 μmol, 2.00 equiv) in anhydrous dimethylformamide (50.0 μL) was added. Tetrakis(triphenylphosphine)palladium(0) (1.73 mg, 6.68 mmol, 0.100 equiv) and copper(I) thiophene-2-carboxylate (5.70 mg, 30 μmol, 2.00 equiv) were added consecutively to the mixture. The mixture was degassed by three freeze-thaw cycles. The reaction was stirred at 24 °C for an additional 8 h under argon pressure. The reaction was quenched by addition of water (5 mL) and ethyl acetate (25 mL). The mixture was transferred into a separatory funnel, and the layers were separated. The aqueous layer was extracted with ethyl acetate (3 × 10 mL). The organic layers were combined and dried over anhydrous sodium sulfate. The solution was dried over a plug of cotton, and the filtrate was concentrated. The residue was dried azeotropically with anhydrous toluene (10 mL) twice. The crude product was purified by column chromatography (SiO_2, 100% ethyl acetate) to afford **S5** as a colorless oil (38.1 mg, 43%).

R_f=0.10 (83%, ethyl acetate/hexane).^{1}H NMR (400 MHz, $(CD_3)_2SO$): 8.00 (brs, 1H), 7.51 (brs, 1H), 2.20 (dd, *J*=7.7, 7.7 Hz, 1H), 6.96 (brs, 1H), 6.80 (dd, *J*=7.7 Hz, 1H), 6.64 (d, *J*=7.7, 2.0 Hz, 1H), 6.43 (brs, 1H), 6.21 (dd, *J*=15.1, 10.8 Hz, 1H), 6.00 (d, *J*=10.8 Hz, 1H), 5.87 (m, 1H), 5.84 (dd, *J*=9.7, 9.7 Hz, 1H), 5.73 (dt, *J*=15.1, 7.0 Hz, 1H), 5.55 (m, 1H), 5.30 (m, 1H), 5.28 (m, 1H), 5.00 (dd, *J*=14.7, 8.0 Hz, 1H), 4.55 (m, 1H), 3.92 (m, 5H), 3.74 (m, 1H), 3.66 (t, *J*=6.5 Hz, 2H), 3.63 (m, 1H). 2.95 (dd, *J*=14.0, 6.8 Hz, 1H), 2.95 (brs, 1H), 2.69 (dd, *J*=14.4, 6.7 Hz, 1H), 2.57 (m, 1H), 2.52 (m, 1H), 2.35 (m, 2H), 2.16 (dd, *J*=-7.0, 7.0 Hz, 1H), 2.15 (dd, *J*=7.0, 7.0 Hz, 1H), 2.02 (m, 1H), 1.90-1.35 (m, 15H), 1.75 (s, 3H), 1.29 (s, 3H), 0.98 (s, 9H, *t*-Bu;TBS), 0.97 (d, *J*=7.0 Hz, 3H), 0.94 (d, *J*=7.0 Hz, 3H), 0.89 (s, 9H, *t*-Bu;TBS), 0.64 (d, *J*=7.0 Hz, 3H), 0.13 (s, 3H, CH_3;TBS), 0.11 (s, 3H, CH_3;TBS), 0.04 (s, 3H, CH_3;TBS), 0.00 (s, 3H, CH_3;TBS). ^{13}C (100 MHz, $(CD_3)_2SO$): 176.4, 171.9, 170.8, 170.0, 157.4, 137.4, 136.3, 134.8, 132.7, 131.9, 130.9, 130.0, 129.8, 127.2, 125.8, 120.5, 117.2, 114.7, 109.7, 79.2, 78.2, 73.4, 64.6, 64.6, 62.8, 59.3, 55.7, 50.2, 44.4, 41.7, 37.9, 37.4, 36.8, 32.7, 32.2, 29.7, 28.6, 28.2, 26.2, 25.9, 25.4, 23.9, 23.0, 19.7, 18.6, 18.2, 18.0, 14.5, 13.6, 12.8, 3.3, 3.5, -4.0, -4.9. IR (ATR-FTIR), cm^{-1}: 3337.9 (br), 3021.3 (w), 2954.6 (s), 2928.4 (s), 2856.0 (s), 1740.6 (s), 1644.6 (s), 1599.8 (s), 1542.4 (s), 1514.6 (s), 1459.0 (s), 1418.6 (s), 1374.7 (s), 1300.7 (s), 1255.4 (s), 1233.7 (s), 1201.5 (s), 1161.0 (s), 1114.5 (s), 1057.3 (s), 1029.9 (s), 1002.3 (s). HRMS-ESI (m/z): $[M+NH_4]^+$ calculated for $C_{57}H_{97}N_5O_{11}Si_2$, 1083.6718; found 1083.6709.

*Synthesis of (3S,6S,9R,10R,11S,12S,13E,15E,18S,21S)-10,12-dihydroxy-3-(3-hydroxybenzyl)-18-((2E,4E)-9-hydroxynona-2,4-dien-2-yl)-6-isopropyl-11-methyl-9-(2-(2-methyl-1,3-dioxolan-2-yl)ethyl)-19-oxa-1,4,7,25-tetraazabicyclo[19.3.1]pentacosa-13,15-diene-2,5,8,20-tetraone (**S6**).*

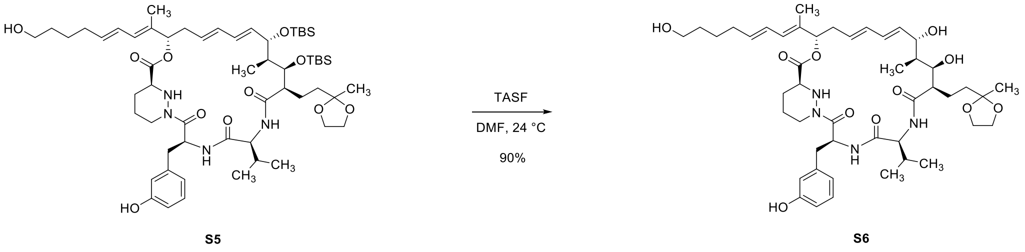

Trisulfonium difluorotrimethylsilicate (TASF) (0.041 mL, 1M, 41.4 μmol, 6.00 equiv) was added to a solution of **S5** (7.46 mg, 6.90 μmol, 1.00 equiv) in anhydrous dimethylformamide (1.0 mL) at 24 °C. The reaction was stirred for 8 h under argon pressure. The reaction was quenched by addition of ethyl acetate (10. mL) and water (10. mL). After stirring for 10 min, the mixture was transferred into a separatory funnel. The layers were separated, and the aqueous layer was extracted with ethyl acetate (2 × 10. mL). The organic layers were combined and dried over anhydrous sodium sulfate. The mixture was filtered through a plug of cotton, and the filtrate was concentrated under reduced pressure. The resulting residue was dried azeotropically with toluene twice. The crude product was purified by column chromatography (SiO_2, 100% ethyl acetate) and recrystallization in ether to afford **S6** (5.30 mg, 90%) as a white amorphous solid.

R_f=0.15 (100%, ethyl acetate).^{1}H NMR (400 MHz, $(CD_3)_2SO$): 9.25 (s, 1H), 8.15 (d, *J*=7.0, 1H), 7.44 (d, *J*=7.9 Hz, 1H), 7.07 (dd, *J*=7.9 Hz, 1H), 6.59 (ddd, *J*=7.9, 2.0 Hz, 1H), 6.57 (dd, *J*=7.9 Hz, 1H), 6.50 (d, *J*=2.0 Hz, 1H), 6.23 (dd, *J*=15.0, 10.9 Hz, 1H), 6.14 (dd, *J*=14.8, 10.5 Hz, 1H), 6.02 (dd, *J*=14.8, 10.5 Hz, 1H), 6.00 (d, *J*=10.9 Hz, 1H), 5.68 (dt, *J*=14.8, 7.1 Hz, 1H), 5.58 (m, 1H), 5.55 (dd, *J*=14.8, 7.0 Hz, 1H), 5.43 (d, *J*=5.1 Hz, 1H), 5.38 (m, 1H), 5.24 (dd, *J*=8.0, 2.2 Hz, 1H), 5.02 (brs, 2H), 4.78 (d, *J*=4.9 Hz, 1H), 4.49 (d, *J*=11.8 Hz, 1H), 4.35 (dd, *J*=5.1, 5.1 Hz, 1H), 4.16 (d, *J*=11.8 Hz, 1H), 3.89 (m, 1H), 3.86-3.73 (m, 6H), 3.37 (t, *J*=6.0 Hz, 2H), 3.24 (brs, 1H), 3.22 (m, 1H), 2.74 (dd, *J*=12.9, 9.24 Hz, 1H), 2.63 (m, 1H), 2.59 (dd, *J*=12.8, 4.0 Hz, 1H), 2.49 (m, 1H), 2.41 (m, 1H), 2.08 (m, 3H), 2.00-1.15 (m, 12H),

1.70 (s, 3H), 1.23 (s, 3H), 0.82 (d, *J*=7.0 Hz, 3H), 0.80 (d, *J*=7.0 Hz, 3H), 0.60 (d, *J*=7.1 Hz, 3H). ^{13}C NMR ((100 MHz, $(CD_3)_2SO$): 175.2, 172.3, 171.2, 170.6, 157.7, 138.7, 136.2, 134.7, 133.3, 131.6, 130.9, 130.3, 130.0, 126.4, 126.2, 120.5, 117.0, 113.6, 109.5, 77.5, 77.0, 74.7, 72.0, 64.5, 61.0, 58.3, 57.8, 55.8, 52.8, 49.3, 44.0, 37.2, 36.7, 32.6, 32.6, 29.8, 27.4, 25.7, 24.1, 22.8, 19.8, 18.7, 14.5, 13.2, 11.3. IR (ATR-FTIR), cm^{-1}: 3295.0 (br), 2955.8 (s), 2925.2 (s), 2872.5 (s), 2854.7 (s), 1732.15 (s), 1636.3 (s), 1599.6 (s), 1588.5 (m), 1553.2 (s), 1514.8 (m), 1485.6 (m), 1458.1 (s), 1379.0 (m), 1333.6 (m), 1308.3 (m), 1255.9 (m), 1236.0 (m), 1208.3 (m), 1163.1 (m), 1099.7(s), 1071.8 (s), 1022.5 (s). HRMS-ESI (m/z): $[M+NH_4]^+$ calculated for $C_{45}H_{69}N_5O_{11}$, 855.4988; found 855.5002.

Synthesis of ***SfA-mc***.

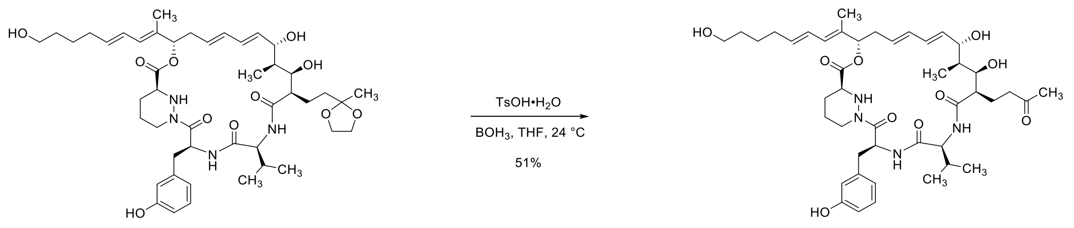

S6 **SfA-mc**

Boric acid (1.92 mg, 31.1 μmol, 5.00 equiv) was added to a solution of **S6** (5.30 mg, 6.21 μmol, 1.00 equiv) in tetrahydrofuran (0.15 mL) at 24 °C. After stirring for 30 min, a solution *p*-toluenesulfonic acid monohydrate (118 μg, 0.621 μmol, 0.100 equiv) in tetrahydrofuran (0.030 mL) was added at 24 °C. The reaction was stirred for 8 h at 24 °C. The reaction was quenched by addition of saturated aqueous sodium bicarbonate (5.0 mL). The mixture was transferred into a separatory funnel containing ethyl acetate (5.0 mL), rinsing with additional ethyl acetate (2 × 5.0 mL). The funnel was swirled gently, and the layers were separated. The aqueous layer was extracted with ethyl acetate (2 × 5.0 mL). The organic layers were combined and dried over anhydrous sodium sulfate. The dried solution was filtered through a plug of cotton, and the filtrate was concentrated. The crude product was purified by column chromatography (SiO_2, 100% ethyl acetate) to afford **SfA-mc** as a white amorphous solid (2.56 mg, 51%).

R_f=0.10 (100%, ethyl acetate). ^1H NMR (400 MHz, $(CD_3)_2SO$): 9.25 (s, 1H), 8.15 (bd, 1H), 7.45 (bd, 1H), 7.07 (dd, *J*=7.6, 7.6 Hz, 1H), 6.60 (dd, *J*=7.6, 2.0 Hz, 1H), 6.57 (d, *J*=7.6 Hz, 1H), 6.51 (s, 1H), 6.23 (dd, *J*=15.0, 10.9 Hz, 1H), 6.14 (dd, *J*=14.2, 10.6 Hz, 1H), 6.04 (dt, *J*=14.7, 5.4 Hz, 1H), 5.96 (d, *J*=10.6, 1H), 5.68 (dt, *J*=15.3, 6.9 Hz, 1H), 5.62 (dd, *J*=14.6, 8.0 Hz, 1H), 5.56 (dd, *J*=15.0, 10.9 Hz, 1H), 5.45 (dd, *J*=13.3, 5.6 Hz, 1H), 5.38 (m, 1H), 5.32 (bt, *J*=5.0 Hz, 1H), 5.24 (m, 1H), 5.02 (brs, 1H), 4.78 (bd, *J*=4.8 Hz, 1H), 4.49 (m, 1H), 4.35 (t, *J*=5.0 Hz, 2H), 4.26-4.00 (m, 2H), 3.94-3.73 (m, 2H), 2.80-2.30 (m, 7H), 2.15-1.93 (m, 4H), 2.06 (s, 3H), 1.70 (s, 3H, H_{32}), 1.84-1.11 (m, 11H), 0.90-0.75 (m, 6H), 0.60 (d, *J*=8.0 Hz, 3H). ^{13}C NMR ((100 MHz, $(CD_3)_2SO$): 208.5, 175.2, 172.3, 171.2, 170.6, 157.7, 138.7, 136.2, 134.7, 133.3, 131.6, 130.9, 130.3, 130.0, 126.4, 126.2, 120.5, 117.0, 113.6, 77.5, 77.0, 74.7, 72.0, 61.0,

58.2, 57.8, 55.8, 52.8, 49.2, 44.1, 37.1, 36.7, 32.6, 31.8, 29.5, 27.0, 25.7, 24.1, 22.6, 19.8, 18.8, 14.4, 13.3, 11.3. IR (ATR-FTIR), cm^{-1}: 3291.0 (br), 3025.0 (w), 2956.8 (s), 2923.4 (s), 2853.4 (s), 1732.5 (s), 1637.0 (s), 1599.3 (m), 1588.2 (m), 1523.2 (m), 1485.9 (m), 1456.5 (m), 1377.3 (m), 1334.6 (m), 1308.7 (m), 1253.2 (m), 1234.8 (m), 1200.6 (m), 1161.6 (m), 1119.7 (m), 1100.1 (m), 1019.7 (s). HRMS-ESI (m/z): $[M+Na]^+$ calculated for $C_{43}H_{61}N_4O_{10}Na$, 816.4280; found 816.4246.

References

1. Pangborn AB, Giardello MA, Grubbs RH, Rosen RK, and Timmers FJ. Safe and Convenient Procedure for Solvent Purification. *Organometallics*. 1996;15(5):1518-20.
2. Still WC, Kahn M, and Mitra A. Rapid chromatographic technique for preparative separations with moderate resolution. *The Journal of Organic Chemistry*. 1978;43(14):2923-5.
3. Heck JA, Meng X, and Frick DN. Cyclophilin B stimulates RNA synthesis by the HCV RNA dependent RNA polymerase. *Biochem Pharmacol*. 2009;77(7):1173-80.
4. Flaxman HA, Chang CF, Wu HY, Nakamoto CH, and Woo CM. A Binding Site Hotspot Map of the FKBP12-Rapamycin-FRB Ternary Complex by Photoaffinity Labeling and Mass Spectrometry-Based Proteomics. *J Am Chem Soc*. 2019;141(30):11759-64.
5. Chang CF, Flaxman HA, and Woo CM. Enantioselective Synthesis and Biological Evaluation of Sanglifehrin A and B and Analogs. *Angew Chem Int Ed Engl*. 2021;60(31):17045-52.

Supplemental Figures for

Sanglifehrin A mitigates multi-organ fibrosis by targeting the collagen chaperone cyclophilin B

Hope A. Flaxman[1], Maria-Anna Chrysovergi[2], Hongwei Han[2], Farah Kabir[1], Rachael T. Lister[2], Chia-Fu Chang[1], Robert Yvon,[1] Katharine E. Black[2], Andreas Weigert[3,4], Rajkumar Savai[4,5,6,7], Alejandro Egea-Zorrilla [6,7], Ana Pardo-Saganta [6,7], David Lagares[2,*], Christina M. Woo[1,*]

[1] Department of Chemistry and Chemical Biology, Harvard University, Cambridge, MA

[2] Fibrosis Research Center, Center for Immunology and Inflammatory Diseases, Division of Pulmonary and Critical Care Medicine, Massachusetts General Hospital, Harvard Medical School, Boston, MA, USA

[3] Goethe-University Frankfurt, Faculty of Medicine, Institute of Biochemistry I, Germany

[4] Frankfurt Cancer Institute (FCI), Goethe University, and German Cancer Consortium (DKTK), Germany

[5] Max Planck Institute for Heart and Lung Research, Member of the German Center for Lung Research (DZL), Bad Nauheim, 61231, Germany.

[6] Institute for Lung Health (ILH), Department of Internal Medicine, Justus-Liebig University, Universities of Giessen and Marburg Lung Center (UGMLC), German Center for Lung Research (DZL), 35392 Giessen, Germany.

[7] Cardio-Pulmonary Institute (CPI), Department of Internal Medicine, Justus Liebig University, 35392 Giessen, Germany.

* Corresponding authors. Emails: Christina M. Woo, 12 Oxford St, Cambridge, MA 02138, 617-495-3858, cwoo@chemistry.harvard.edu; David Lagares, 857-264-8078, davidlagares@sloan.mit.edu

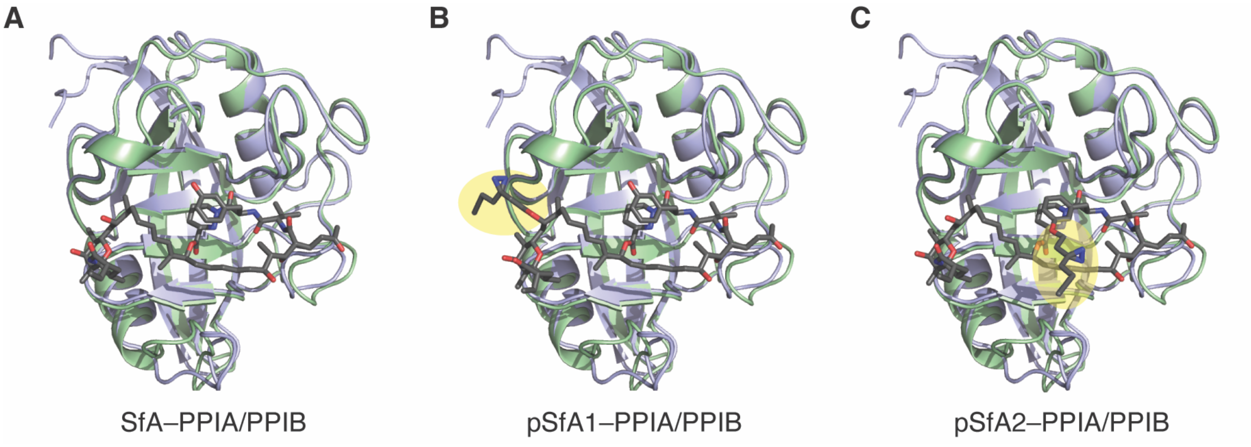

Supplemental Figure 1: Model of SfA and analogs pSFA1 or pSFA2 overlaid on PPIA and PPIB. A, Crystal structure of the SfA–PPIA complex (green, PDB: 1YND) aligned with PPIB (blue, PDB: 3ICI). **B**, Model of pSfA1 overlaid on PPIA and PPIB. **C**, Model of pSfA2 overlaid on PPIA (green) and PPIB (blue). Photo-affinity tag highlighted in yellow. Models of pSfA1 and pSfA2 were derived from SfA and the chemical modification was minimized in Molecular Operating Environment 2020.

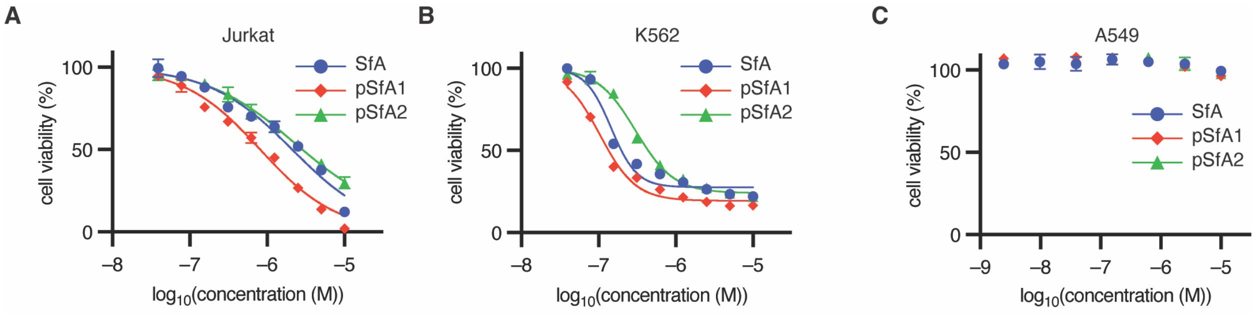

Supplemental Figure 2: Effect of indicated compounds on Jurkat (**A**), K562 (**B**), and A549 (**C**) cell viability, as determined in a 72 h MTT assay (n = 3). MTT data is shown as means ± SD, with 3 replicates per condition.

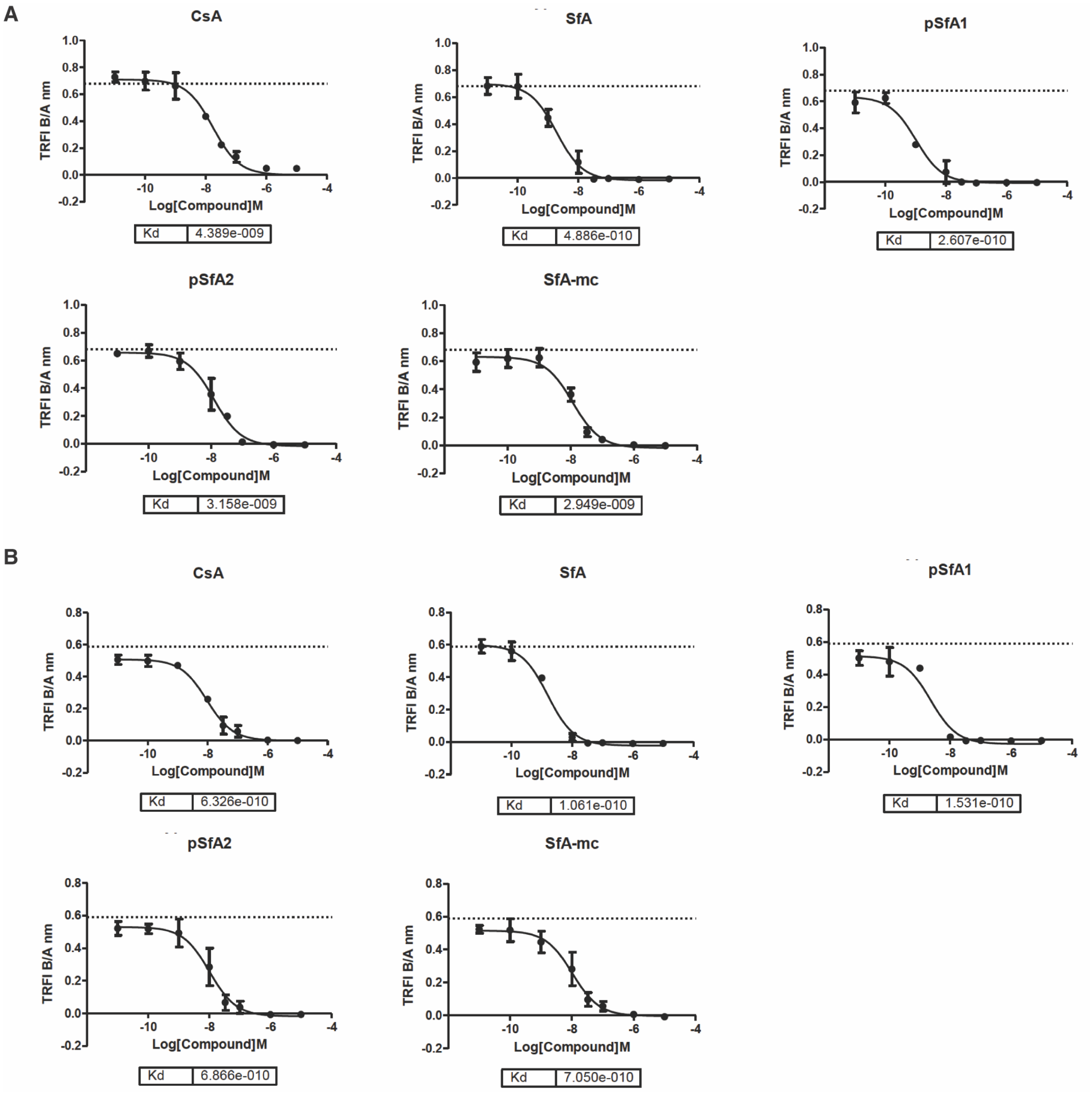

Supplemental Figure 3: Determination of dissociation constants for SfA, pSfA1, pSfA2, and SfA-mc to PPIA (**A**) and PPIB (**B**), using a standard protocol performed by Eurofins (n = 2). Dissociation constants were determined relative to CsA in a competitive TR-FRET assay, with each compound assayed over a concentration range of 0.01 nM–10 μM.

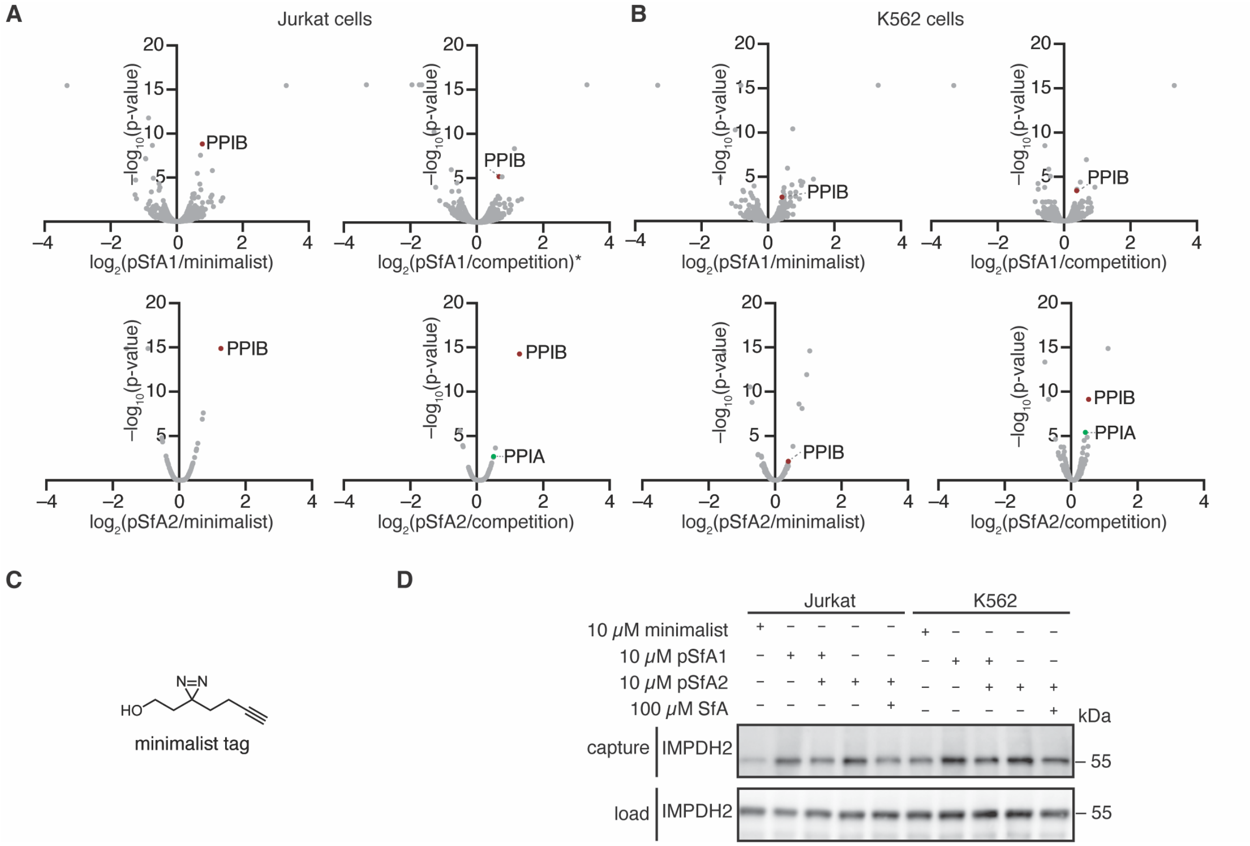

Supplemental Figure 4: Photo-affinity labeling with sanglifehrin analogs and chemical proteomics analysis. A, Volcano plots of chemoproteomics data from Jurkat cells (n = 3). PPIB is marked in red on each plot. PPIA is marked in green in ratios where it was significantly enriched. * n = 2, due to loss of one sample in the comparison of pSfA1/competition. **B,** Volcano plots of chemoproteomics data from K562 cells (n = 3). PPIB is marked in red on each plot. PPIA is marked in green in ratios where it was significantly enriched. **C,** Structure of the minimalist tag used as a negative control in chemoproteomics experiments. **D,** Western blot of enrichment of IMPDH2 with pSfA1 and pSfA2 from Jurkat and K562 cells.

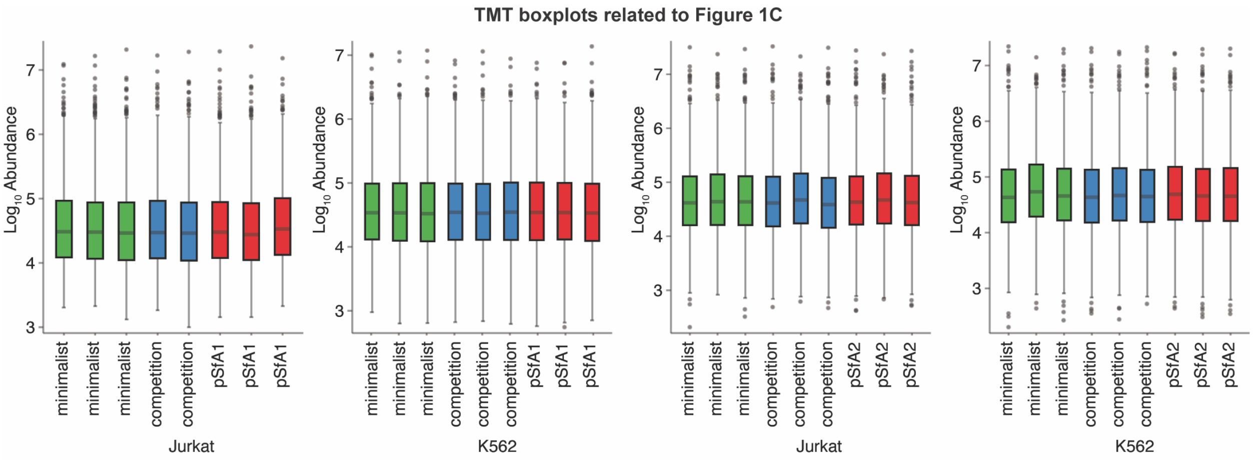

Supplemental Figure 5: Boxplot of protein level normalization using TMT. Normalization was performed against the total peptide amount in Proteome Discoverer 2.4.

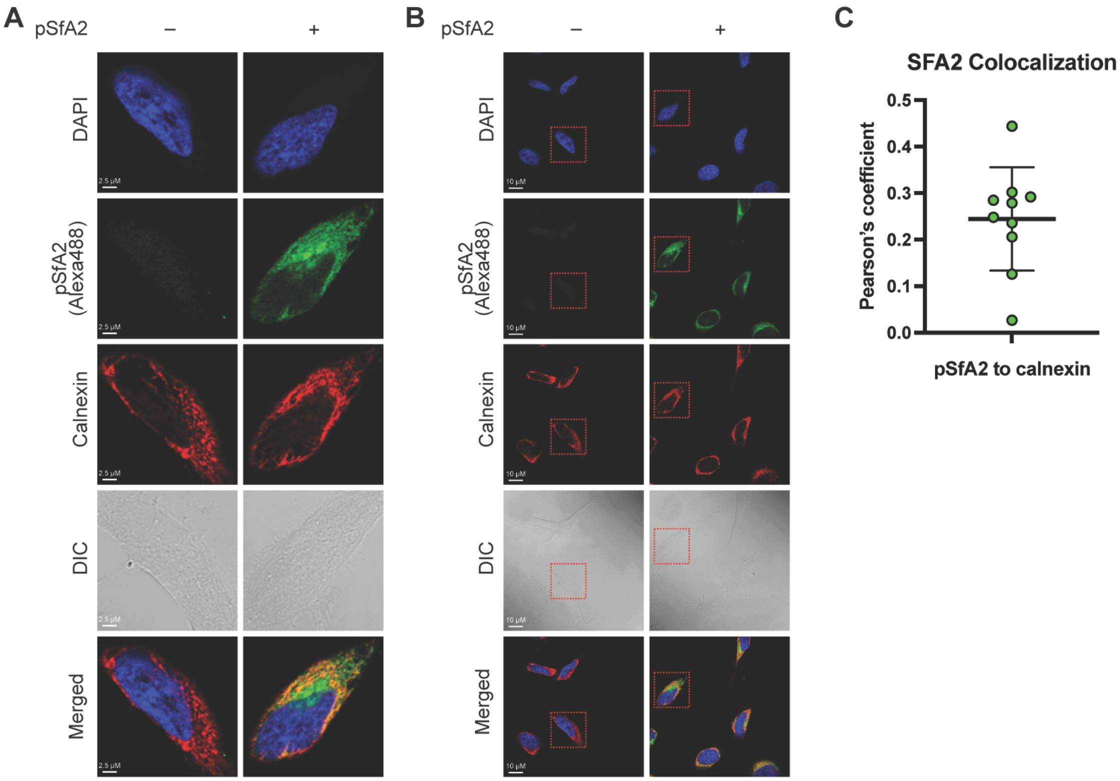

Supplemental Figure 6: Immunofluorescence of pSfA2. A–B, Immunofluorescence imaging of calnexin in HeLa cells treated with or without 100 µM pSfA2 for 30 minutes. Cells were UV irradiated, labeled with Alexa488 azide, and imaged. Nuclei were stained and imaged with DAPI. Zoomed images shown in **A**, scale bars shown (2.5 μm). **B,** Representative full immunofluorescence images from **A**. Scale bars shown (10 μm), red squares indicate regions enlarged shown in **A** (4x). **C,** Plot of Pearson's Correlations calculated between calnexin and pSfA2. Each point represents a single image.

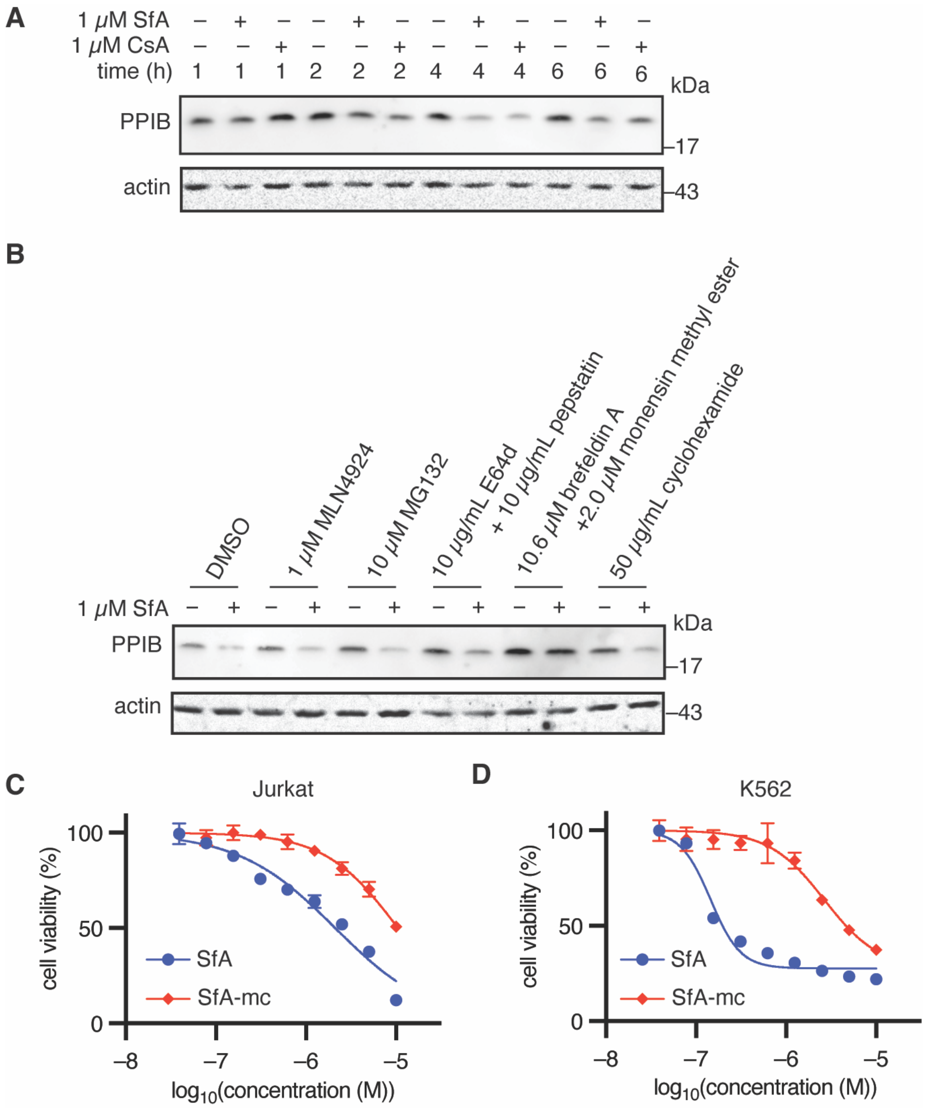

Supplemental Figure 7: Comparison of SfA, CsA, and SfA-mc on PPIB levels and cell viability. A, Western blot of intracellular PPIB in Jurkat cells treated with 1 μM SfA or 1 μM CsA for the indicated time. **B,** Western blot of intracellular PPIB in Jurkat cells pretreated with the indicated inhibitors for 1 h prior to treatment with 1 μM SfA for 4 h. **C,** Activity of SfA and SfA-mc in a 72 h MTT assay in Jurkat cells. SfA data is replicated from Supplemental Figure 2A (n = 3). **D,** Activity of SfA and SfA-mc in a 72 h MTT assay in K562 cells (n = 3). SfA data is replicated from Supplemental Figure 2B (n = 3). MTT data is shown as means ± SD, with 3 replicates per condition.

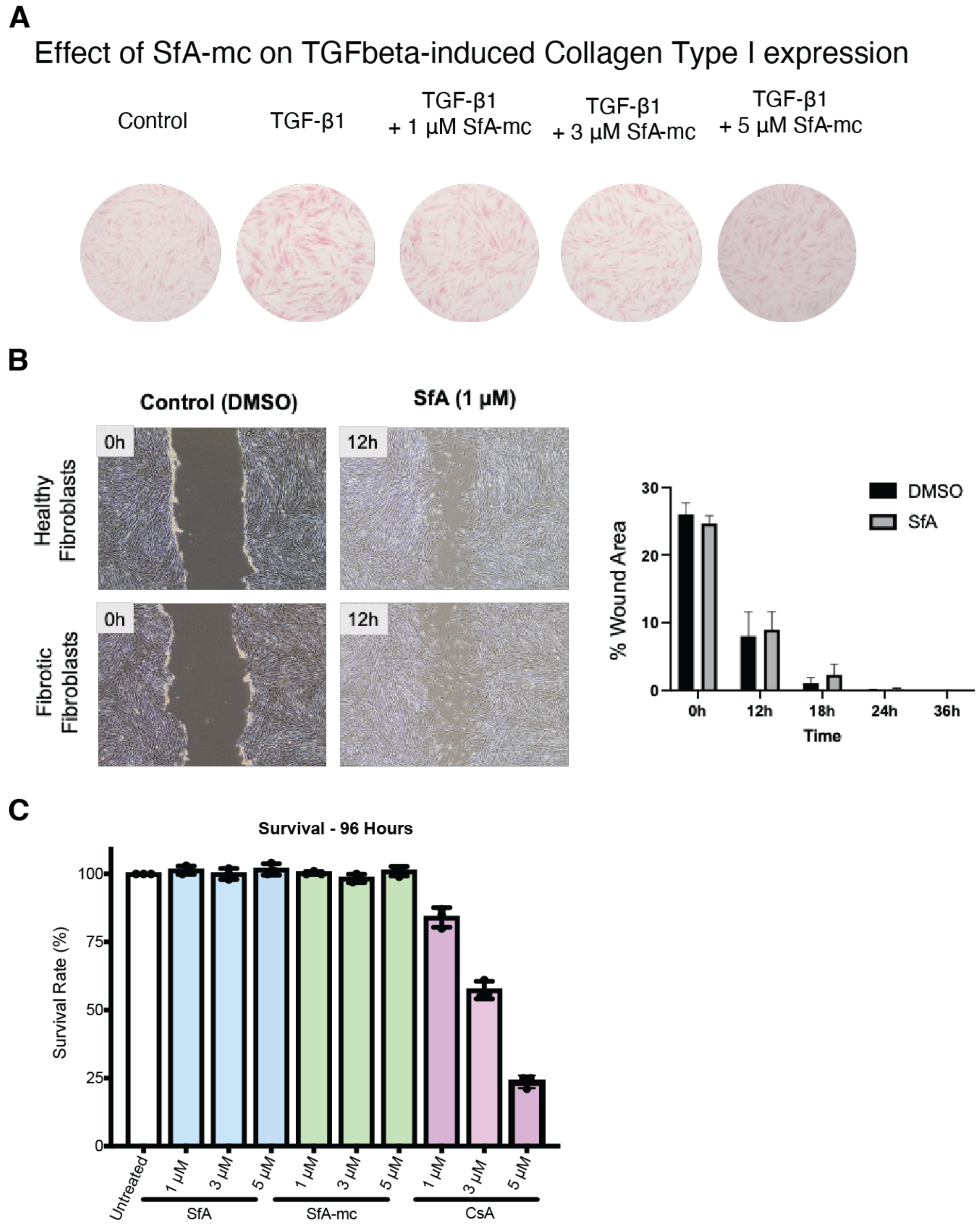

Supplemental Figure 8: Effects of SfA and SfA-mc on collagen production, wound healing assay, and cell survival. **A,** Intracellular collagen visualized by sircol staining following stimulation with 10 ng/mL TGF-β1 ± 1 μM SfA-mc for 96 h in an IMR-90 fibroblast model of fibrosis. **B,** Wound healing scratch assay with human primary fibroblast from healthy donors treated with or without 10 ng/mL TGF-beta and ± 1 μM SfA, in triplicate. Wound closure was quantified using ImageJ. **C,** Survival of IMR-90 fibroblasts as determined by trypan blue staining following treatment with the indicated compounds for 96 h in serum-free media. All graphed data represents means ± standard deviation (SD).

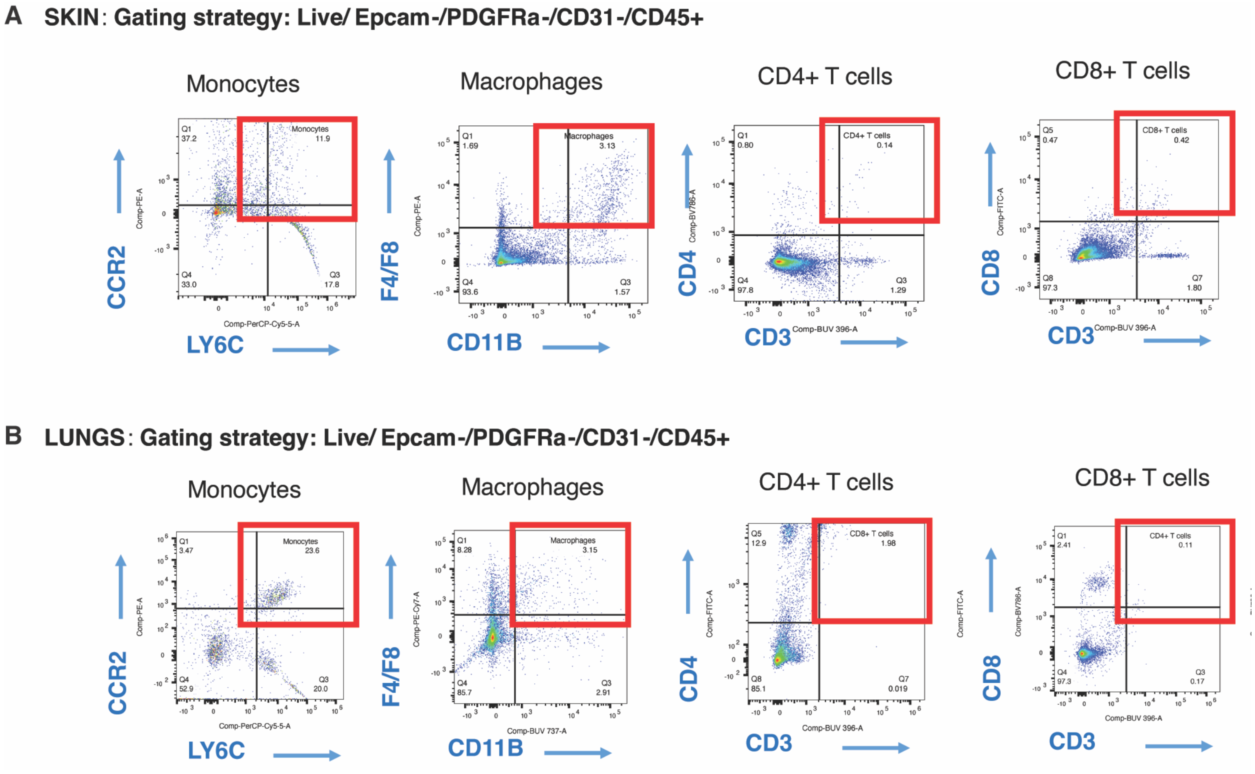

Supplemental Figure 9: Gating schemes for flow cytometry data. Gating schemes for data shown in Fig. 4E (**A**) and 5E (**B**).

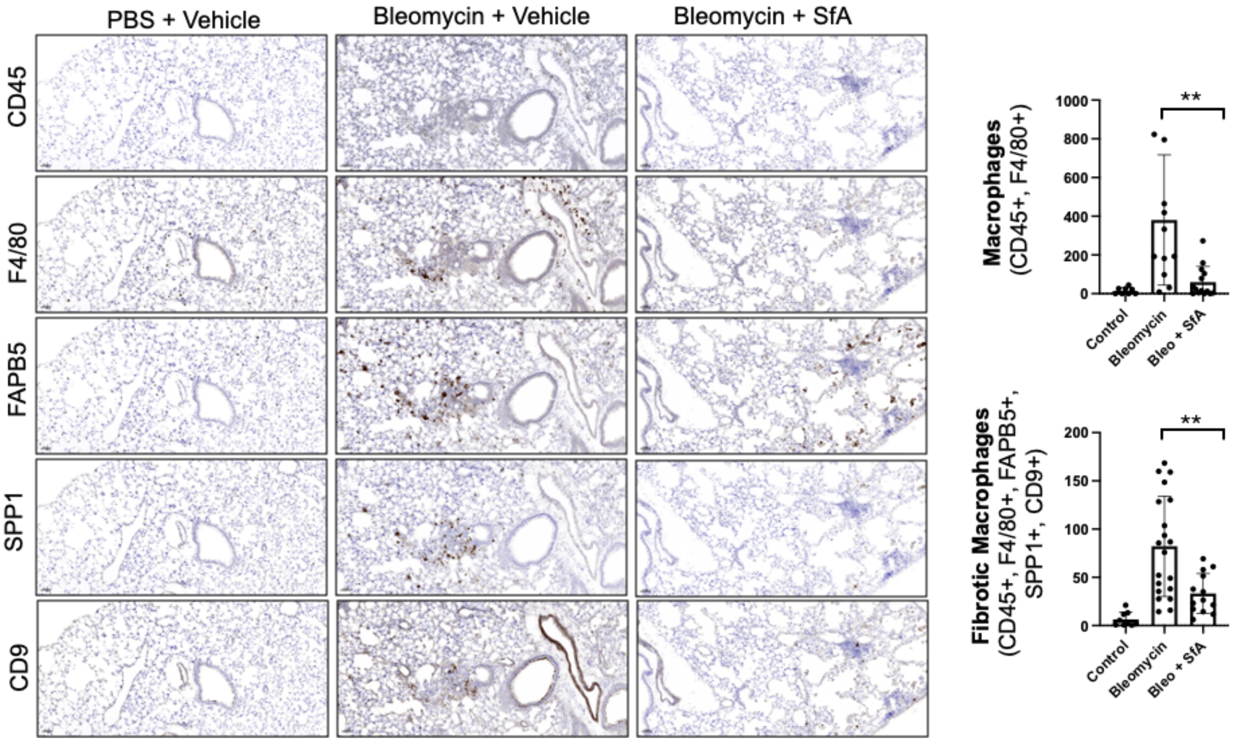

Supplemental Figure 10: Profiling of profibrotic macrophages by multiplex imaging. Profiling of macrophages (identified as CD45+, F4/80+ cells) and profibrotic macrophages (identified as CD45+, F4/80+, FAPB5+, SPP1+, CD9+ cells) from lung tissue sections after in vivo treatment with bleomycin +/– SfA treatment. Lung tissue sections were stained sequentially with the indicated antibody. Nuclei were counterstained with DAPI and imaged. Images were analyzed using the inForm software.

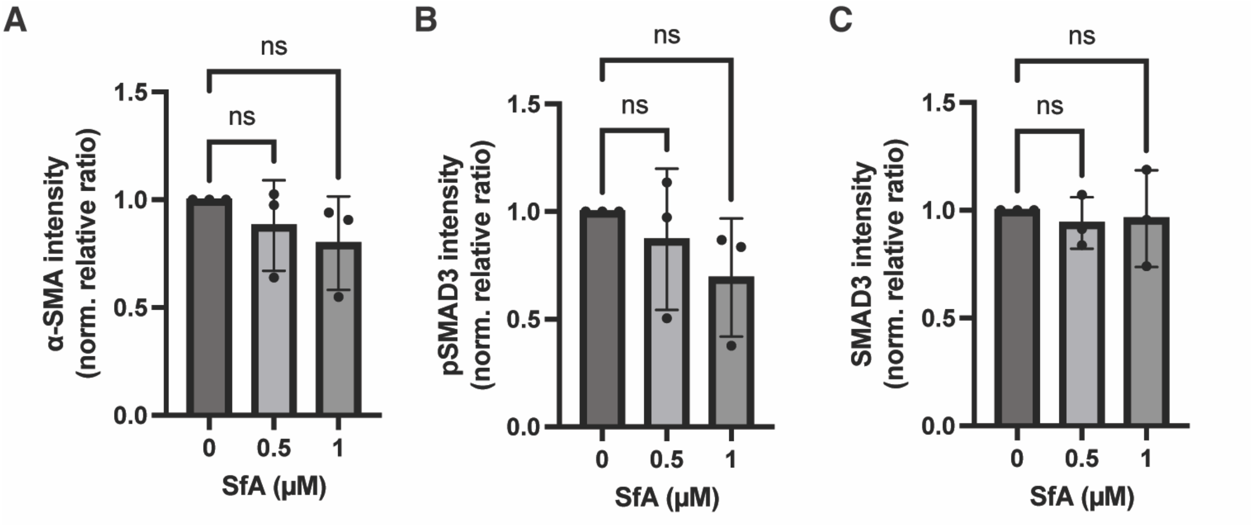

Supplemental Figure 11: Quantification of Figure 6J. Quantification of Western blot for **A,** α-smooth muscle actin (αSMA), **B,** phosphoSMAD3, and **C,** SMAD3 levels in IPF fibroblasts ± SfA over 96 h in cell lysates. GAPDH was used as loading control.

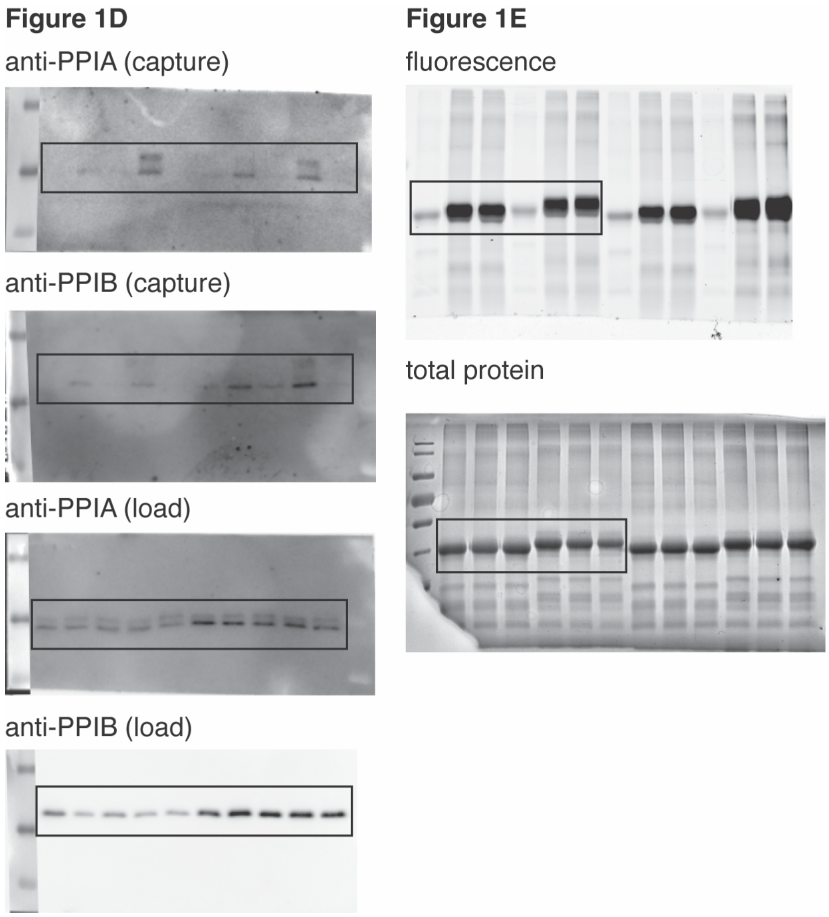

Supplemental Figure 12: Uncropped full Western Blots related to Figure 1.

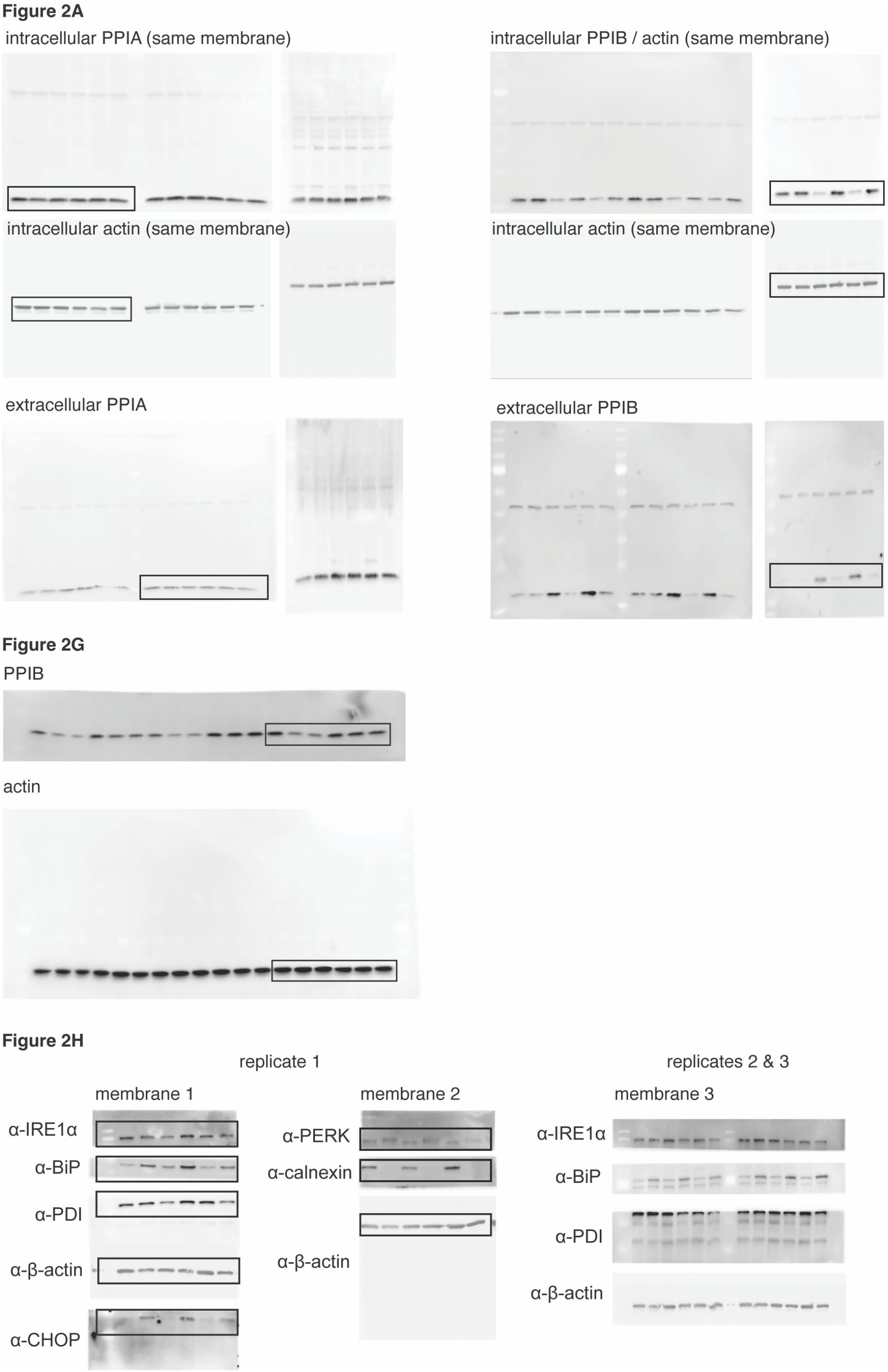

Supplemental Figure 13: Uncropped full Western Blots related to Figure 2.

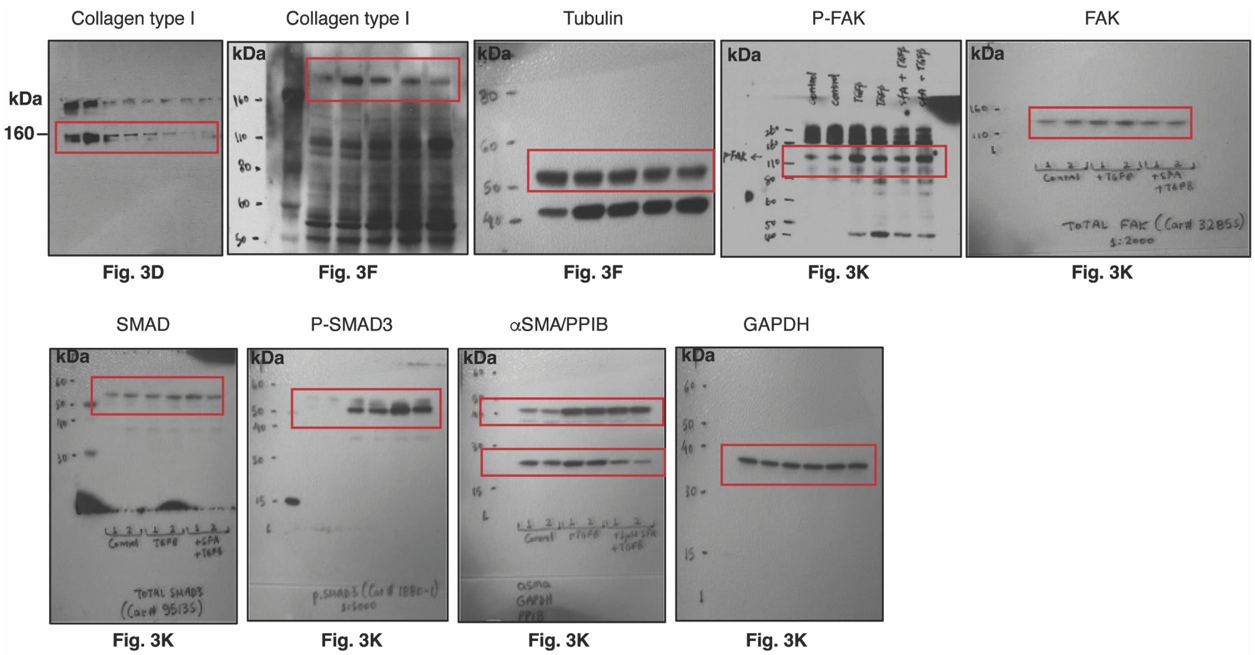

Supplemental Figure 14: Uncropped full Western Blots related to Figure 3.

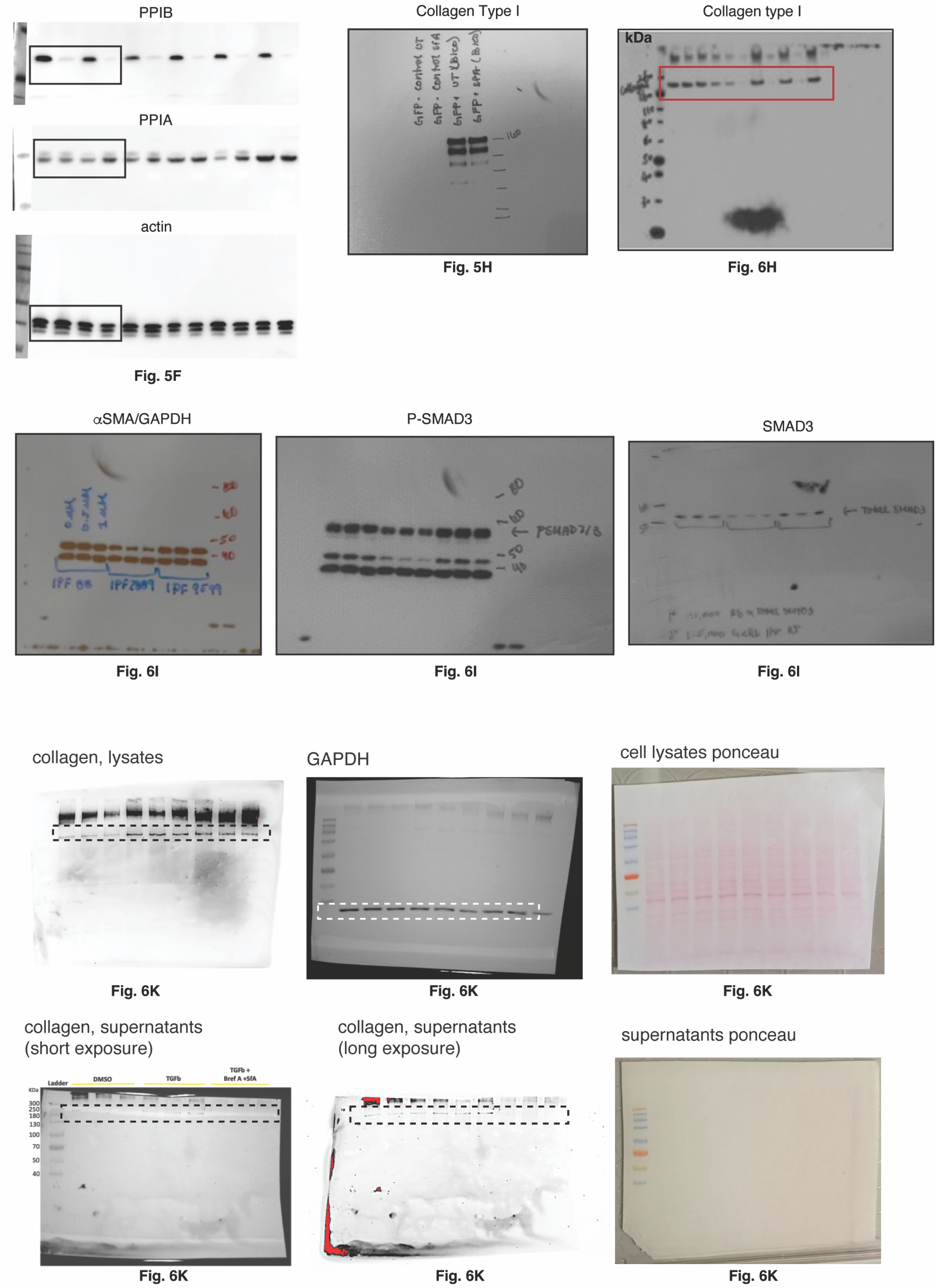

Supplemental Figure 15: Uncropped full Western Blots related to Figure 5 and Figure 6.

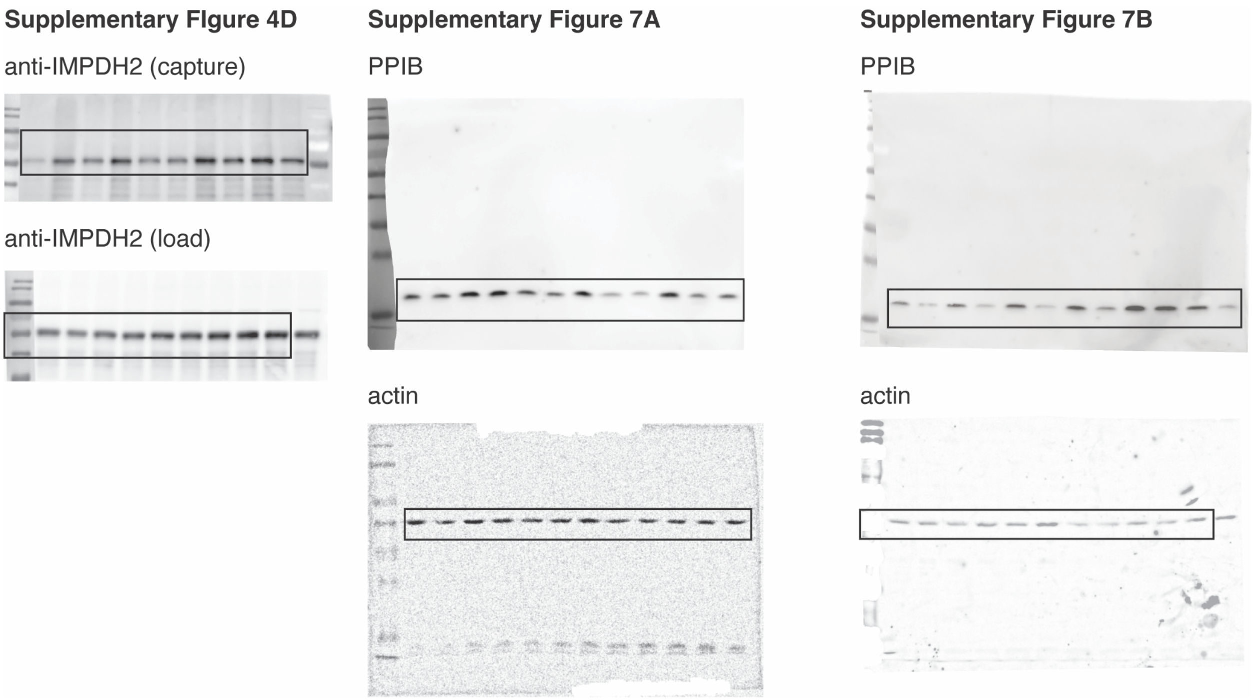

Supplemental Figure 16: Uncropped full Western Blots related to Supplemental Figure 4, Supplemental Figure 7.
