# Supplementary material for: Sanglifehrin A mitigates multiorgan fibrosis by targeting the collagen chaperone cyclophilin B
Source: JCI Insight. 2024 Jun 20;9(15):e171162. doi: 10.1172/jci.insight.171162 (PMC11383833; doi:10.1172/jci.insight.171162)
Supplement: Unedited blot and gel images [file jciinsight-9-171162-s123.pdf]

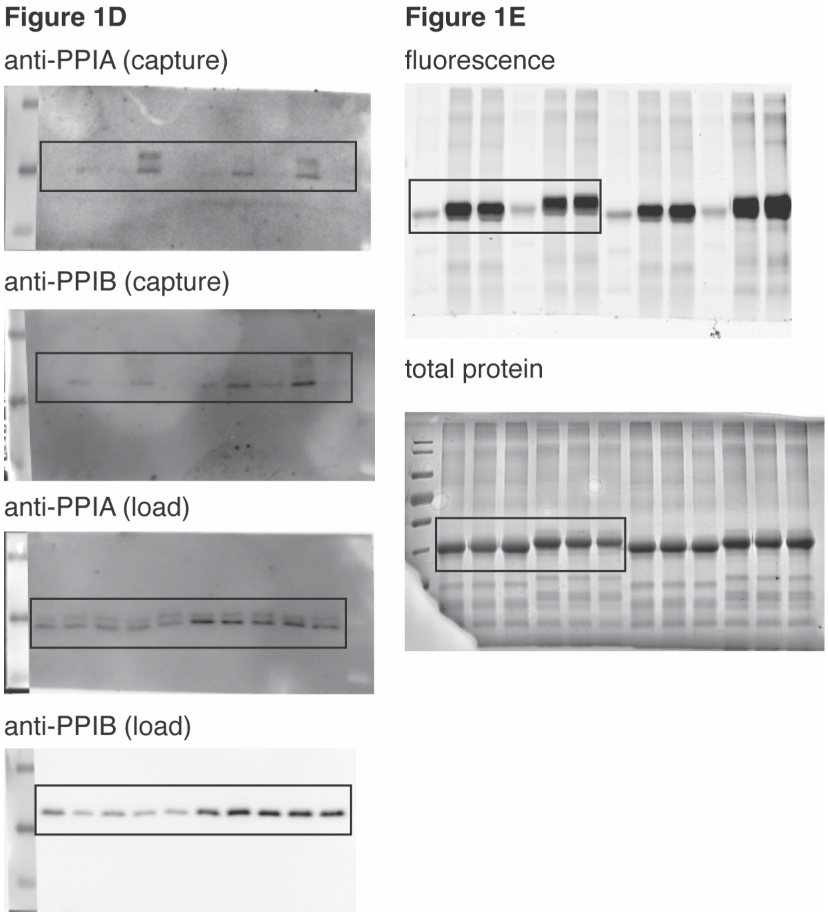

Supplementary Figure 11: Uncropped full Western Blots related to Figure 1.

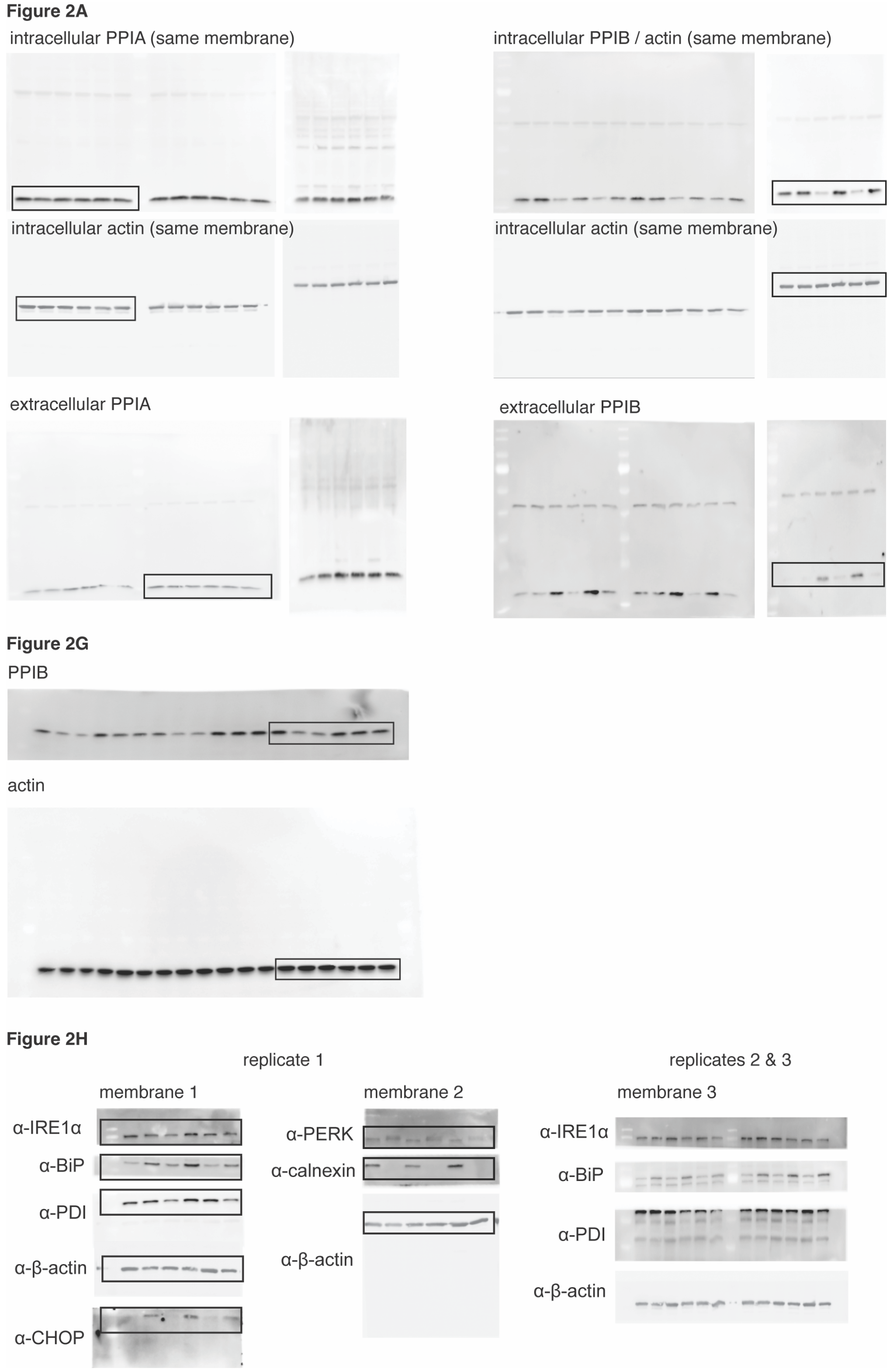

Supplementary Figure 12: Uncropped full Western Blots related to Figure 2.

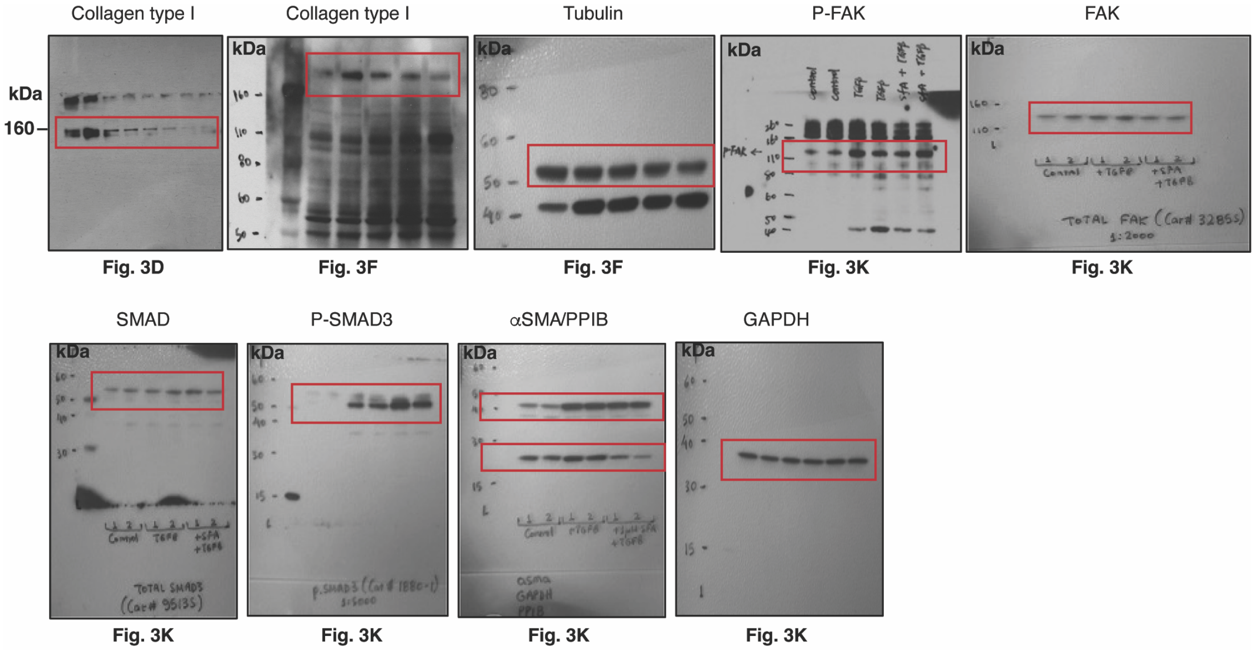

Supplementary Figure 13: Uncropped full Western Blots related to Figure 3.

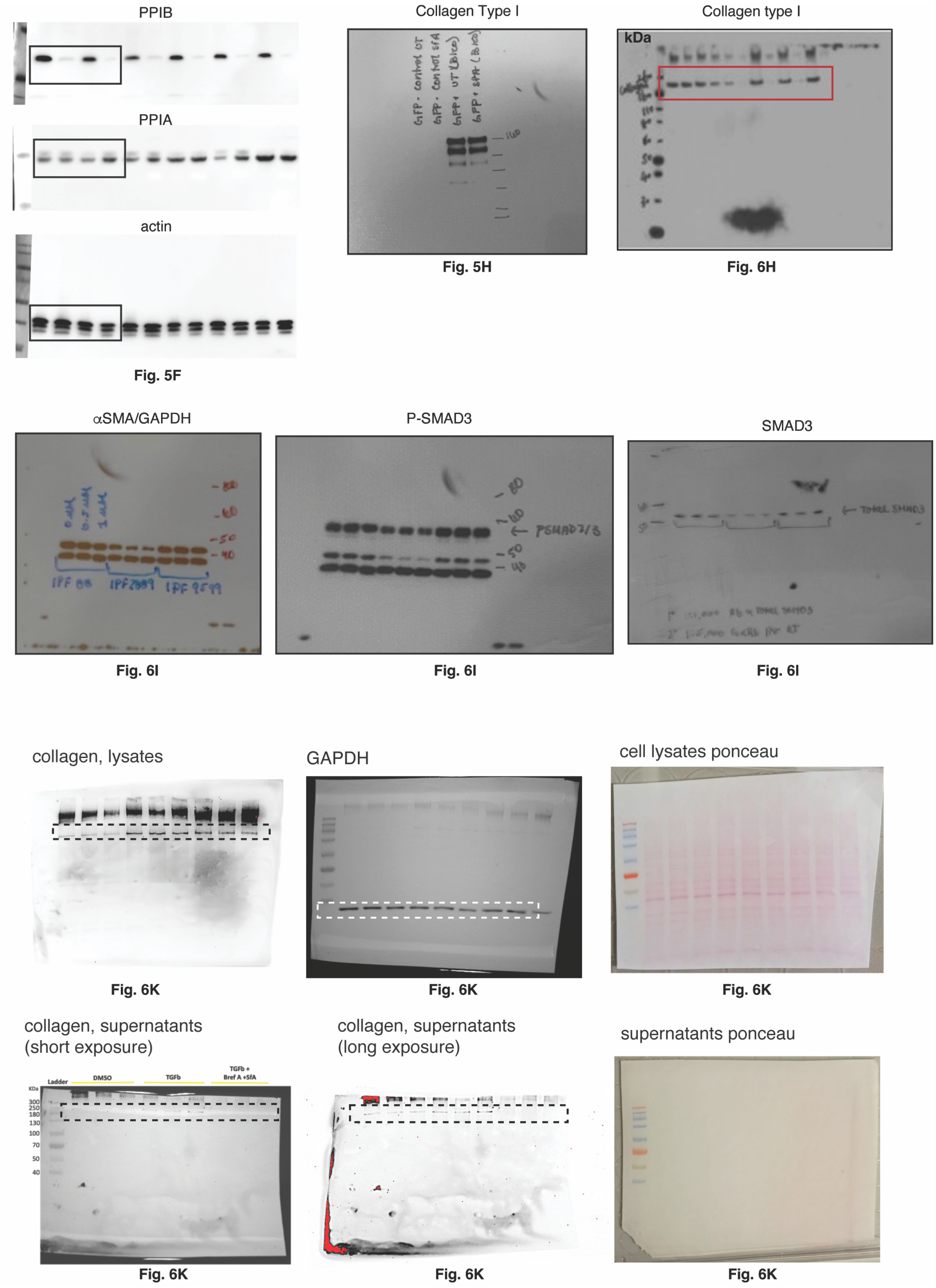

Supplementary Figure 14: Uncropped full Western Blots related to Figure 5 and Figure 6.

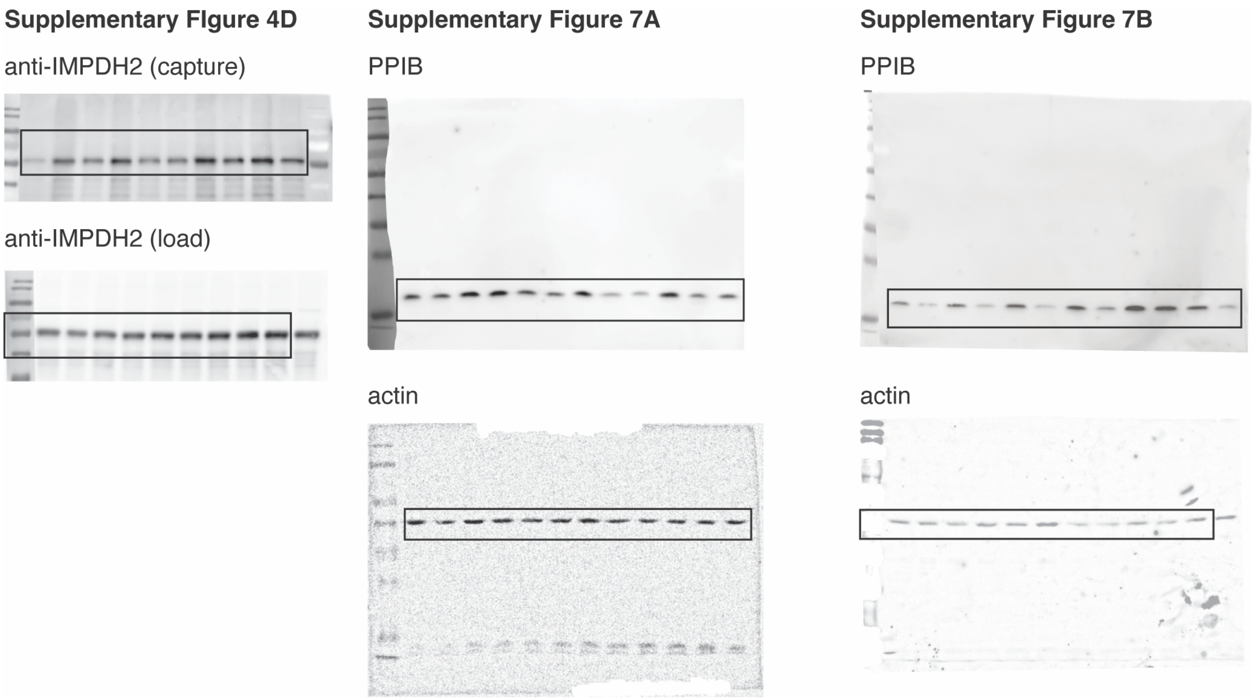

Supplementary Figure 15: Uncropped full Western Blots related to Supplementary Figure 4, Supplementary Figure 7.
